# Synthesis and Characterization of 1,10-Phenanthroline-Linked Imidazolium Salts: Investigation of Their Cytotoxicity Properties

**DOI:** 10.1021/acsomega.5c13398

**Published:** 2026-04-28

**Authors:** Aslıhan Karaer Tunçay, Suleyman Ilhan, Harika Atmaca, Hayati Türkmen

**Affiliations:** † Department of Chemistry, Faculty of Science, Ege University, Bornova, Izmir 35100, Türkiye; ‡ Department of Biology, Faculty of Engineering and Natural Sciences, 52953 Manisa Celal Bayar University, Manisa 45140, Türkiye

## Abstract

This study focuses on the synthesis, characterization, and anticancer activities of 1,10-phenanthroline-linked imidazolium salts **(3)** bearing different alkyl groups (methyl­(a), butyl­(b), octyl­(**c**), dodecyl­(**d**)) or benzyl (benzyl­(**e**), 2,4,6-trimethylbenzyl­(**f**)) groups. The synthesized salts were fully characterized using various analytical techniques, including UV–vis, ^1^H NMR, ^13^C NMR, FT-IR, and mass spectrometry. Their biological activities were evaluated against DU-145 (prostate cancer), MCF-7 (breast cancer), T98G (glioblastoma cancer), and the nontumorigenic HEK-293 (human embryonic kidney) cell lines. The most potent compound **3e** exhibited a remarkable DNA binding constant (K_*b*_ = 1.66 ± 0.1 × 10^6^ M^–1^), indicating a strong interaction with DNA. Additionally, molecular docking studies were performed to assess the interaction of **3e** with key apoptotic and cell cycle regulatory proteins (Bcl-2, Bcl-xL, CDK1, and Cyclin B1), suggesting a potential mechanism for its anticancer activity. ADME-related analyses of the lead compound **3e** were conducted, providing an initial pharmacokinetic profile to support its progression as a preclinical candidate. In cancer cell lines, mitochondrial morphology and integrity were evaluated at IC_50_ concentrations against compound **3e** using Invitrogen MitoTracker stain. These findings highlight the dual role of 1,10-phenanthroline-linked imidazolium salts as DNA-interacting agents and selective anticancer compounds, paving the way for their further development as targeted chemotherapeutic candidates.

## Graphical abstract

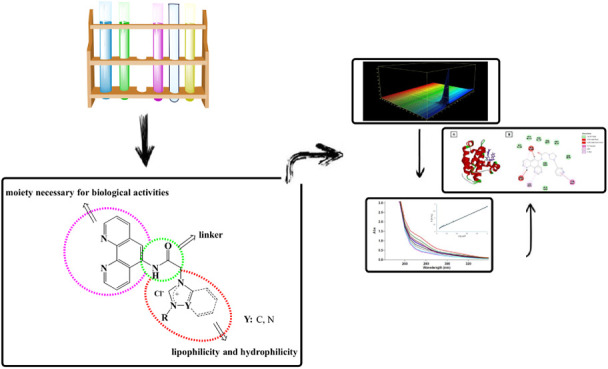

## 1 Introduction

According to the World Health Organization (WHO), cancer accounted for approximately ten million deaths globally in 2020, making it one of the foremost public health challenges of our time.^1^ Despite breakthroughs in medical science and the pharmaceutical industry, cancer remains one of the leading causes of morbidity and mortality worldwide.^2^ These factors underscore that cancer is in critical need of significant research, innovation and investment, requiring the development of new therapeutic agents with high selectivity and efficacy. Organic ligands have been investigated for their potential in drug design, particularly in the development of novel anticancer and antiviral agents. The interaction of these ligands with biological targets can lead to significant therapeutic effects, as evidenced by ongoing research on their pharmacological properties.^3^

In recent years, heterocyclic compounds, particularly those containing 1,10-phenanthroline and imidazolium cores, have garnered significant attention due to their DNA-intercalating ability and interaction with biological macromolecules such as proteins and nucleic acids. 1,10-phenanthroline molecule and its derivatives represent a significant area of interest in medicinal chemistry, particularly for their anticancer properties.^4^ The 1,10-phenanthroline moiety is well-known for its capability to form metal complexes, stabilize G-quadruplex structures, and intercalate into DNA, making it a promising scaffold for anticancer drug development.^5,6^ Another important feature of 1,10-phenanthroline is its planar structure, which allows intercalation or groove binding with DNA or RNA.^7^ Binding to DNA occurs through intercalation, a noncovalent interaction, under the influence of large aromatic ligands.^8^ Similarly, imidazolium salts exhibit cytotoxic activity against various cancer cell lines due to their structural compatibility with DNA and ability to disrupt key protein interactions involved in apoptosis and cell cycle regulation. Imidazole derivatives have garnered significant attention due to their potent therapeutic properties, prompting medicinal chemists to develop a wide array of novel chemotherapeutic agents. These imidazole-based compounds have broadened the potential for advancing various aspects of clinical medicine, offering promising improvements in treatment options.^9^ It can be considered that the electronic structure of the imidazole derivative ring exerts a remarkable influence on the physical properties and chemical reactivity tendencies of imidazole-containing materials.

The 2-aryl group of 1*H*-imidazo­[4,5-*f*]­phenanthroline has received significant attention in the context of antitumor activity. In 2013, Wei et al. investigated the interaction between 4-(1*H*-Imidazo­[4,5-*f*]-1,10-phenanthrolin-2-yl)­phenol derivative compounds and G-quadruplex DNA structures, as well as the subsequent effects of these interactions on their biological activities. The study demonstrated that these compounds effectively bind to G-quadruplex DNA structures, and this binding induces disruption of the cell cycle through the inhibition of telomerase activity. Furthermore, the compounds were found to exhibit cytotoxic effects against cancer cells, thereby inhibiting their proliferation.^10^ In 2016, Yu et al. found that L233, a newly synthesized phenanthroline derivative, damages DNA and activates apoptosis pathways in response to this damage. They also reported that this compound inhibits the G1/S transition, inhibits cell cycle progression, and suppresses the proliferation of cancer cells.^11^ In 2019, Wang et al. synthesized a series of imidazo­[4,5-*f*]­[1,10]­phenanthroline derivatives. The most potent compound effectively induced autophagy in cancer cells and initiated DNA damage and apoptosis processes.^12^ Wang et al. investigated the interaction between phenanthroimidazole derivatives and c-myc G-quadruplex DNA structures, demonstrating that this interaction leads to DNA stabilization. Furthermore, the study revealed that these compounds exhibited antitumor activity, inhibited cell proliferation in CNE-1 cancer cells, and induced cellular death processes, including apoptosis.^13^ Mei et al. studied a series of imidazole phenanthroline derivatives reporting high yield synthesis of these derivatives using microwave irradiation. One of the compounds exhibited promising inhibitory effects against cancer and HepG2 cells with an IC_50_ value of 0.68 μM.^14^ Chen et al. evaluated 1*H*-imidazo­[4,5-*f*]­[1,10]­phenanthroline (IPM713) in seven different cancer cell lines and reported that it exhibited the most significant inhibitory effect on the colorectal cancer cell line HCT116 with an IC50 value of 1.7 μM.^15^ Arias-Ṕerez et al. designed a series of 1*H*-imidazo­[4,5-*f*]­[1,10]­phenanthroline derivatives modified at the 2-position with polyhydroxy alkyl chains derived from carbohydrates. They showed relevant and different cytotoxic activities against different cultured tumor cell lines PC3, HeLa and HT-29. One of them showed significant antibacterial activity, high membrane selectivity, and low toxicity against Gram-positive bacteria and clinical MRSA isolates (MIC = 0.5–2 μg/mL).^16^ A study showing that 1,10-phenanthroline-substituted imidazolium salts have significant inhibitory activity against MDA-MB-231 cells with an IC_50_ value of 12.8 ± 1.2 μM has been previously reported by our group.^17^

Despite extensive studies on imidazole-fused 1,10-phenanthroline derivatives, there is a lack of research focusing on unfused analogs of these compounds. Therefore, this study aims to synthesize and characterize a novel series of new unfused molecules by connecting imidazole and 1,10-phenanthroline via the acetamide bridge, investigate their DNA-binding properties, and evaluate their anticancer potential against DU-145 (prostate cancer), MCF-7 (breast cancer), and T98G (glioblastoma) cell lines, as well as nontumorigenic HEK-293 cells. We also compared the effect of imidazole derivatives containing different aliphatic or benzylic groups in molecules. In addition to standard cytotoxicity assays, we conducted UV–vis spectroscopy to determine the DNA-binding constant (K_*b*_), molecular docking studies on key apoptotic and cell cycle regulatory proteins (Bcl-2, Bcl-xL, CDK1, Cyclin B1), and apoptosis assays to elucidate the potential mechanisms of action. These findings provide insights into the biological relevance of imidazolium salts as potential chemotherapeutic agents targeting DNA and apoptotic pathways.

## 2 Results and Discussion

### 2.1 Chemistry

#### 2.1.1 Synthesis of 1,10-Phenanthroline-Linked Imidazolium Salts

In this study, we aimed to investigate whether the effect of 1,10-phenanthroline and imidazole bearing alkyl or benzyl group are important in determining cytotoxic activity (Scheme 2). In the first step, 5-nitro-1,10-phenanthroline **(1)** was prepared from a mixture of 1,10-phenanthroline with concentrated H_2_SO_4_ and fuming HNO_3_. Second, 5-nitro-1,10-phenanthroline **(1)** was reduced to 5-amino-1,10-phenanthroline **(2)** with hydrazine monohydrate in an argon atmosphere. Next, 2-chloro-*N*-(1,10-phenanthrolin-5-yl)­acetamide **(3)** was synthesized by adding the appropriate acid chloride at room temperature (Scheme 1). Consequently, 1,10-phenanthroline-linked imidazolium salts **(3a–h)** were prepared by refluxing 2-chloro-*N*-(1,10-phenanthroline-5-yl)­acetamide **(3)** with excess imidazole bearing alkyl (methyl­(**a**), butyl­(**b**), octyl­(**c**), dodecyl­(**d**)) or benzyl (benzyl­(**e**) and 2,4,6-trimethylbenzyl­(**f**)) groups.

**Scheme 1:**
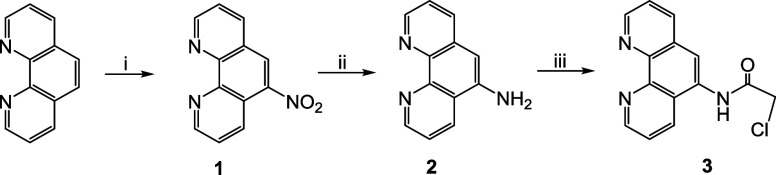
Synthesis of 1,10-Phenanthroline Acetamide, **3** ^a^

a Reaction conditions: (i) HNO_3_, H_2_SO_4_, 160 °C, reflux, 4 h; (ii) Pd/C 10%, NH_2_NH_2_.H_2_O, ethanol, 70 °C, reflux, 12 h; (iii) ClCOCH_2_Cl, Et_3_N, dichloromethane, room temperature (RT), 20 h.

To compare the effect of the imidazole group, salts **3g** and **3h** containing benzimidazole and triazole, respectively, were prepared. An attempt to synthesize 1,10-phenanthroline-linked azolium salts (**3a–h)** was performed by following the procedure used as shown in Scheme 2. The salts **(3a–h)** were obtained in 52–94% yields as an air- and moisture-stable solids with good solubility in polar solvents, such as DMSO, alcohol and H_2_O. They were characterized using elemental analysis, ^1^H-, ^13^C NMR, Fourier transform infrared (FT-IR) spectroscopies, and mass spectrometry. The purity of all of the salts **(3a–h)** was determined via elemental analysis. The purity of the compound **3e** was been evaluated using techniques like high pressure liquid chromatography (HPLC) in addition to mass spectrometry and elemental analysis (see Figures S42 and S34–41). The ^1^H, ^13^C NMR and FT-IR analysis results for the compounds are provided in the Supporting Information. The formation of ligands was consistent with the evaluation of chemical shifts and splitting in the assignment of the ^1^H NMR resonances of the ligands. The N–H resonance at δ = 11.11–11.74 ppm as a sharp singlet in the ^1^H NMR spectra indicated the formation of amide protons. N–CH–N resonance at δ = 10.37–9.12 ppm as a sharp singlet in the ^1^H NMR spectra, indicating the formation of imidazolium salts. The chemical shifts of sp^2^ carbon atoms (N–C–N) of imidazolium salts **(3a–h)** in the ^13^C NMR spectrum are 150.46, 150.34, 150.29, 150.27, 150.54, 150.11, 150.32, and 150.41 ppm, respectively (Table 1). The ^1^H, ^13^C NMR analysis results for the salts are provided in the Supporting Information (see Figures S1–S22). The FT-IR spectra of the ligand salts are similar to each other. Normally, the N–H stretching vibration bands are in the region of 3450 cm^–1^.^18^ The N–H stretching vibration band peaks in salts are in the range of 3136–3451 cm^–1^. The strength of hydrogen bonding typically leads to broader peaks and lower frequency stretching.^19^ Aromatic C–H stretching modes exhibit bands in the 3000–3100 cm^–1^ region, while aliphatic C–H stretching modes exhibit bands below 3000 cm^–1^.^20^ In the characteristic IR spectrum of the salts **(3a–h)**, ν­(C=O) and ν­(C=N) show bands in the range of 1675–1698 and 1129–1192 cm^–1^, respectively. FTIR analysis results of the salts are included in the Supporting Information (see Figures S23–S33).

**Scheme 2:**
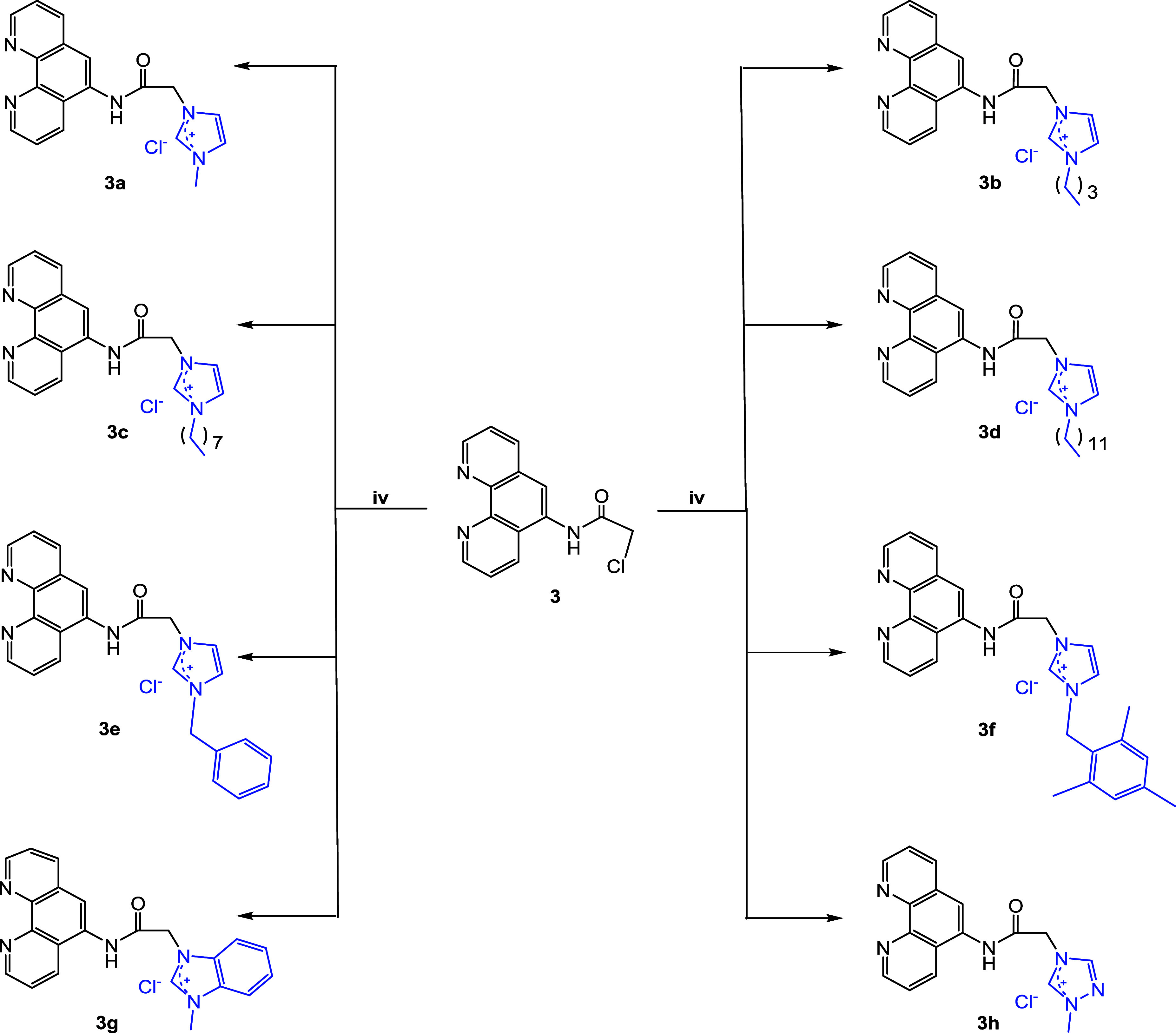
Synthesis of 1,10-Phenanthroline-Linked Imidazolium Salts[Fn sch2-fn2]

a Reaction conditions: (iv) excess of NHC, 110 °C, reflux, overnight.

**Table 1:** Log*P* Values, ^1^H–^13^C NMR and Melting Point Data of Compounds **3a–h**

			^1^H NMR (ppm)		^13^C NMR (ppm)		
Entry	**Compound**	Log*P*	N–H	N–CH–N	N–C–N	C=O	Melting Point (°C)
1	**3a**	5.45	11.36	9.31	150.46	165.90	150
2	**3b**	6.89	11.39	9.39	150.34	165.95	134
3	**3c**	8.65	11.41	9.38	150.29	165.95	130
4	**3d**	9.37	11.48	9.41	150.27	165.96	87
5	**3e**	7.05	11.37	9.49	150.32	165.93	178
6	**3f**	8.22	11.12	9.12	150.41	165.93	245
7	**3g**	6.96	11.11	9.85	150.54	165.56	315
8	**3h**	5.12	11.74	10.37	150.11	165.39	210

In drug development, poor solubility is an important factor limiting the bioavailability of lipophilic drugs.^21^ As the proportion of poorly soluble drug candidates increases, researchers are developing various strategies to increase solubility, such as formulation techniques, salt forms, amorphous forms, and liposomes and nanocarriers.^22,23^ Determining the physicochemical properties of a drug is a critical step in the early stages of drug discovery.^24^ Log*P* indicates the difference between the lipophilicity (affinity for oil) and hydrophilicity (affinity for water) of the drug, which directly affects its solubility, permeability and ultimately bioavailability.^25^ A higher Log*P* indicates that a molecule is more lipophilic (oil soluble), while a lower value indicates that it is more hydrophilic (water-soluble). Log*P*, also known as the partition coefficient, measures the distribution of a molecule between a hydrophobic (usually octanol) and hydrophilic (water) phase.^26^ The calculated Log*P* values are given in Table 1. ALOGPS 2.1 and Molinspiration, suitable for log*P* calculations, are useful computational tools for researchers in drug design, materials science, and related fields where understanding the balance between solubility and lipophilicity is critical.^27,28^ The lipophilicity properties of 1,10-Phenanthroline-linked imidazolium salts **(3a–h)** were investigated. The set represents a wide range of chemical structures and lipophilicity (5.45 < c log*P <* 9.37). In particular, the length of the alkyl chain increased the lipophilicity of the salts (**3a–d**). At the same time, the addition of a methyl group to the phenyl ring has the effect of increasing lipophilicity **(3e**, **3f)**. The presence of a methyl group typically contributes to the hydrophobic character of a molecule, increasing its lipophilicity (higher Log*P* value).^29^

### 2.2 Pharmacology/Biology

#### 2.2.1 Cytotoxic Effects of Synthesized Salts **(3a–h)** on Cancer and Normal Cells

The cytotoxicity of salts **(3a–h)** was evaluated against DU-145 prostate cancer, MCF-7 breast cancer, T98G glioblastoma, and HEK-293 nontumorigenic human embryonic kidney cells over a 72-h period, with *cis*-platin (CP) and doxorubicin (DOX) as reference compounds (Table 2). The IC_50_ values, expressed in μM, indicate the concentration required to inhibit cell viability by 50%, offering insight into the relative potency of each compound across cell types. In DU-145 cells, salts **3b**, **3e**, and **3f** demonstrated notable cytotoxicity with IC_50_ values of 25.8 ± 1.0 μM, 22.6 ± 0.8 μM, and 21.5 ± 0.6 μM, respectively, in close range to the IC_50_ of *cis*-platin (18.2 ± 1.2 μM). The lowest IC_50_ was seen with doxorubicin (3.9 ± 0.7 μM), suggesting it as the most potent agent in this cell line. **3b**, **3e**, and **3f** showed extremely low IC_50_ values of 0.1 ± 0.1 μM, 0.03 ± 0.01 μM, and 0.07 ± 0.02 μM, respectively, indicating high cytotoxic potency against MCF-7 cells. These values surpass those of both reference compounds, with *cis*-platin and doxorubicin having IC_50_ values of 16.9 ± 2.2 μM and 4.9 ± 1.0 μM, respectively. In T98G cells, the salts **3a**, **3b** and **3g** exhibited relatively moderate IC_50_ values of 34.7 ± 0.7 μM, 38.4 ± 0.8 μM and 25.7 ± 0.5 μM, respectively. Cytotoxic effects on HEK-293 cells were substantially lower across most compounds, reflecting higher IC_50_ values. Salt **3f** exhibited the lowest IC_50_ of 38.6 ± 0.9 μM in this nontumorigenic cell line, while salts such as **3c** and **3h** showed much higher IC_50_ values (246.8 ± 2.5 μM and 295.6 ± 3.1 μM, respectively). *Cis*-platin and doxorubicin had IC_50_ values of 17.7 ± 0.4 μM and 5.2 ± 1.4 μM, respectively, indicating greater toxicity toward HEK-293 cells than the synthesized compounds. Salts **3b**, **3e**, and **3f** showed the highest cytotoxicity, particularly in MCF-7 breast cancer cells, outperforming reference compounds. Overall, the results suggest that **3e** may have selective anticancer potential, especially in breast cancer, with lower toxicity toward noncancerous HEK-293 cells, which highlights their potential therapeutic window.

**Table 2:** IC_50_ Values and Selectivity Index (SI) Values of Salts **(3a–h)** and Reference Drugs (CP: *cis*-Platin, DOX: Doxorubicin) against DU-145 Prostate Cancer, MCF-7 Breast Cancer, T98G Glioblastoma, and Non-Tumorigenic HEK-293 Cells at 72 h^a^,^b^,^c^

	**IC**_**50**_ **Value (μM)**				**SI Value (μM)**		
**Salt**	DU-145	MCF-7	**T98G**	HEK-293	DU-145	MCF-7	**T98G**
**3a**	36.3 ± 1.1	19.8 ± 0.5	34.7 ± 0.7	149.1 ± 1.8	4.11	7.53	4.30
**3b**	25.8 ± 1.0	0.1 ± 0.1	38.4 ± 0.8	73.3 ± 1.2	2.84	733.0	1.91
**3c**	28.4 ± 0.7	3.1 ± 0.2	40.6 ± 0.6	246.8 ± 2.5	8.69	79.61	6.08
**3d**	45.3 ± 0.9	33.6 ± 0.4	52.4 ± 1.6	114.1 ± 1.4	2.52	3.40	2.18
**3e**	22.6 ± 0.8	0.03 ± 0.01	41.4 ± 1.5	125.4 ± 1.0	5.55	4180.0	3.03
**3f**	21.5 ± 0.6	0.07 ± 0.02	43.9 ± 3.8	38.6 ± 0.9	1.80	551.4	0.88
**3g**	36.9 ± 0.9	9.9 ± 0.8	25.7 ± 0.5	93.8 ± 1.7	2.54	9.47	3.65
**3h**	41.8 ± 1.4	18.9 ± 0.6	57.0 ± 1.3	295.6 ± 3.1	7.07	15.64	5.19
CP	18.2 ± 1.2	16.9 ± 2.2	15.7 ± 2.1	17.7 ± 0.4	0.97	1.05	1.13
DOX	3.9 ± 0.7	4.9 ± 1.0	3.2 ± 1.2	5.2 ± 1.4	1.33	1.06	1.63

a IC_50_ values are expressed in μM and represent the concentration required to reduce cell viability by 50% following a 72 h exposure.

b Values are presented as mean ± standard deviation (SD).

c Lower IC_50_ values indicate higher cytotoxic potency.

To assess the therapeutic selectivity of the synthesized imidazolium salts, the Selectivity Index (SI) was calculated for each compound using the ratio of IC_50_ values obtained in nontumorigenic HEK-293 cells to those measured in cancer cell lines (SI = IC_50_ HEK-293/IC_50_ cancer). The results are summarized in Table 2. Most compounds exhibited SI values greater than 1 across all tested cancer cell lines, indicating preferential cytotoxicity toward malignant cells over normal kidney cells. Notably, compound **3e** demonstrated exceptional selectivity against the MCF-7 breast cancer cell line, with an SI value of approximately 4180, reflecting a highly favorable therapeutic window. This pronounced selectivity significantly exceeded that of the reference compounds cisplatin (CP) and doxorubicin (DOX), which exhibited SI values close to unity. The remarkable selectivity profile of compound **3e** correlates with its strong DNA-binding affinity and favorable interaction with apoptotic regulatory proteins observed in molecular docking and apoptosis assays, supporting its nomination as a lead compound for further preclinical development.

#### 2.2.2 Cell Cycle Distribution of DU-145, MCF-7, and T98G Cells Treated with **3e**

The effects of compound **3e** on cell cycle progression were evaluated in prostate cancer (DU-145), breast cancer (MCF-7), and glioblastoma (T98G) cell lines using flow cytometry (see Figure 1). In untreated DU-145 cells, 0.2% of the cells were in the Sub-G1 phase, indicating minimal apoptosis or cell death, while the majority of cells were distributed in the G0/G1 (50.4%), S (34.6%), and G2/M (14.8%) phases. Upon treatment with compound **3e** at its IC_50_ concentration, notable changes in cell cycle distribution were observed. The Sub-G1 population decreased slightly to 0.1%, suggesting that the treatment did not significantly induce apoptosis at this stage. However, a pronounced reduction in the G0/G1 population (28.7%) was accompanied by a substantial increase in the S phase (46.6%), indicating that **3e** causes an accumulation of cells in the DNA synthesis phase. This was further supported by an increase in the G2/M phase to 26.6%, suggesting a delay in cell cycle progression.

**Figure 1:**
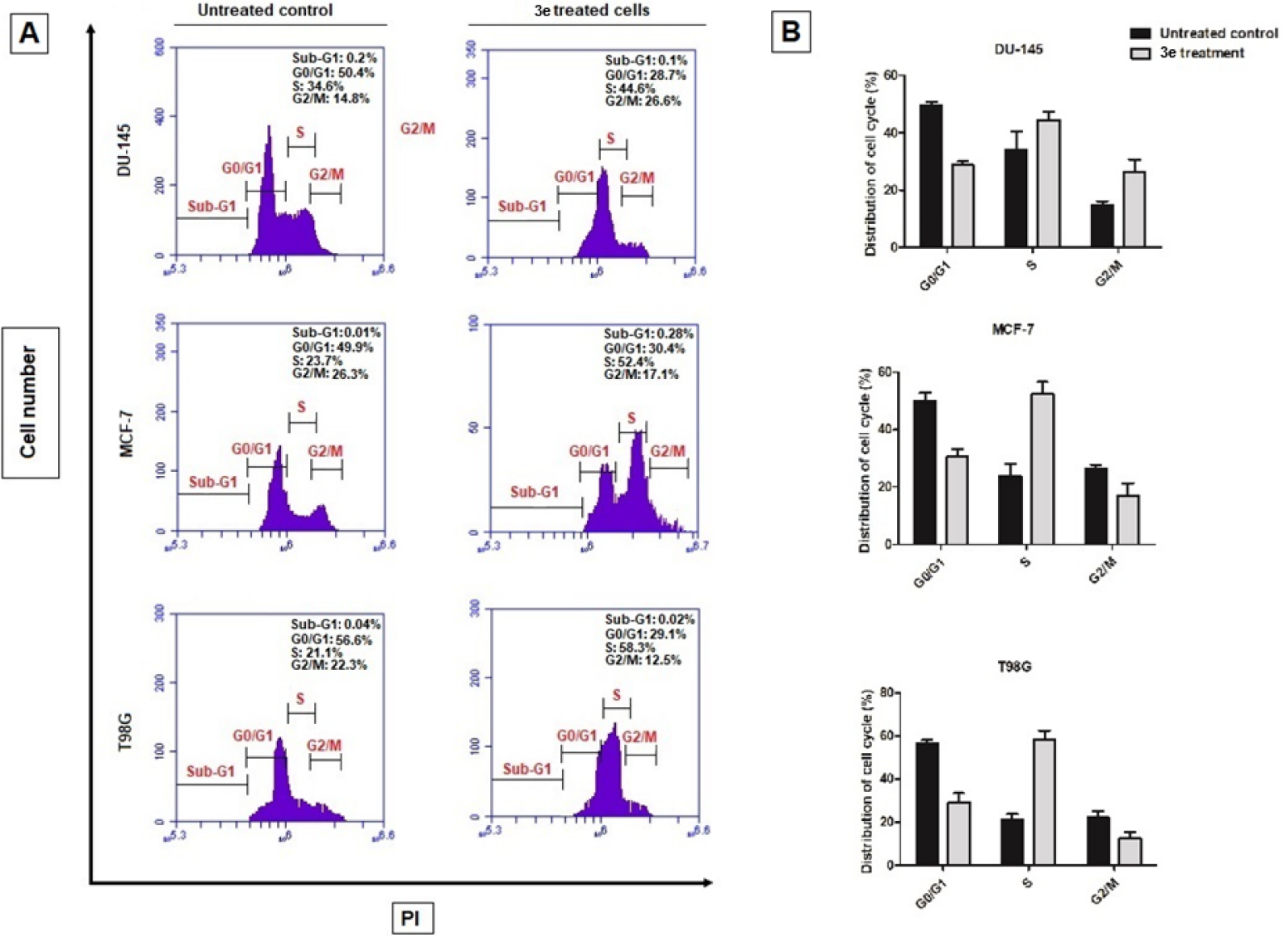
Effects of **3e** on cell cycle distribution in DU-145, MCF-7, and T98G cells. (A) Representative flow cytometry histograms showing the distribution of cells in different phases of the cell cycle (Sub-G1, G0/G1, S, and G2/M) for untreated control and **3e**-treated cells. DU-145 (prostate cancer), MCF-7 (breast cancer), and T98G (glioblastoma) cells were treated with **3e** at its IC_50_ concentration. Notable changes in cell cycle distribution were observed post-treatment, with an increase in the S phase population across all cell lines, indicating S phase arrest, and a corresponding decrease in G0/G1 and G2/M phases. Minimal induction of apoptosis was detected, as indicated by the low Sub-G1 percentages. (B) Quantitative analysis of the distribution of cells in the G0/G1, S, and G2/M phases for untreated control and **3e**-treated cells. Data represent the percentage of cells in each phase of the cell cycle. Error bars represent the standard error of the mean (SEM) for three independent experiments.

In MCF-7 breast cancer cells, the untreated control group had a low Sub-G1 phase (0.01%), with the majority of cells residing in G0/G1 (49.9%), followed by S (23.7%) and G2/M (26.3%) phases. Treatment with compound **3e** induced a slight increase in the Sub-G1 phase to 0.28%, indicating limited induction of apoptosis. Interestingly, the G0/G1 phase population decreased to 30.4%, while the S phase showed a sharp increase to 52.4%, suggesting a significant arrest of cells in the S phase. A corresponding decrease in the G2/M population to 17.1% was also observed, indicating that fewer cells were progressing to mitosis after treatment with **3e**.

In glioblastoma T98G cells, the control group displayed a similar distribution, with 0.04% in the Sub-G1 phase, 56.6% in G0/G1, 21.1% in S, and 22.3% in G2/M. After treatment with **3e**, the Sub-G1 phase remained low at 0.02%, implying that apoptosis was not a major outcome. However, the G0/G1 population significantly dropped to 29.1%, while the S phase rose dramatically to 58.3%, again indicating a strong S phase arrest induced by the compound. The G2/M phase was reduced to 12.5%, similar to the pattern observed in MCF-7 cells, where fewer cells were progressing into mitosis following treatment.

Overall, the data suggest that **3e** exerts its cytotoxic effects primarily by inducing cell cycle arrest in the S phase across all tested cancer cell lines. This S phase accumulation, along with a concurrent decrease in G0/G1 and G2/M populations, implies that the compound may inhibit DNA synthesis and disrupt cell cycle progression, contributing to its potential as an anticancer agent. However, apoptosis was only minimally induced, as indicated by the low percentages of Sub-G1 populations in all cell lines.

#### 2.2.3 Apoptotic Effects of **3e** on Cancer Cells

The pro-apoptotic effects of **3e** on DU-145 (human prostate cancer), MCF-7 (human breast cancer) and T98G (human glioblastoma cancer) cells were evaluated using flow cytometry with Annexin V-FITC and PI staining (see Figure 2). The percentages of viable, early apoptotic, and late apoptotic cells in untreated controls and **3e**-treated cells were compared. In the untreated control group, the majority of cells were viable (90% and 88%), with a minimal percentage of cells in the early apoptotic (1% and 2%) and late apoptotic (10% and 8%) stages. After treatment with **3e**, a significant reduction in the viable cell population was observed (42% and 44%), accompanied by a marked increase in early apoptotic cells (18% and 23%) and late apoptotic cells (32% and 28%). These results suggest that **3e** induces a strong apoptotic response in MCF-7 cells. Untreated DU-145 cells exhibited a high percentage of viability (90% and 88%), with 8% and 12% of cells in the early apoptotic phase and 2% and 3% in the late apoptotic phase. Upon treatment with **3e**, the viable cell population dramatically decreased (28% and 30%), while the early apoptotic (28% and 34%) and late apoptotic (38% and 44%) populations significantly increased. This indicates that **3e** is effective in promoting apoptosis in DU-145 cells. In the T98G control group, 95% and 97% of the cells were viable, while 5% and 6% were in the early apoptotic phase and 2% and 3% in the late apoptotic phase. After **3e** treatment, the viability of T98G cells significantly decreased (37% and 42%), and a substantial increase in both early apoptotic (22% and 23%) and late apoptotic (42% and 37%) cells was observed, indicating that **3e** induces a strong apoptotic effect in T98G cells.

**Figure 2:**
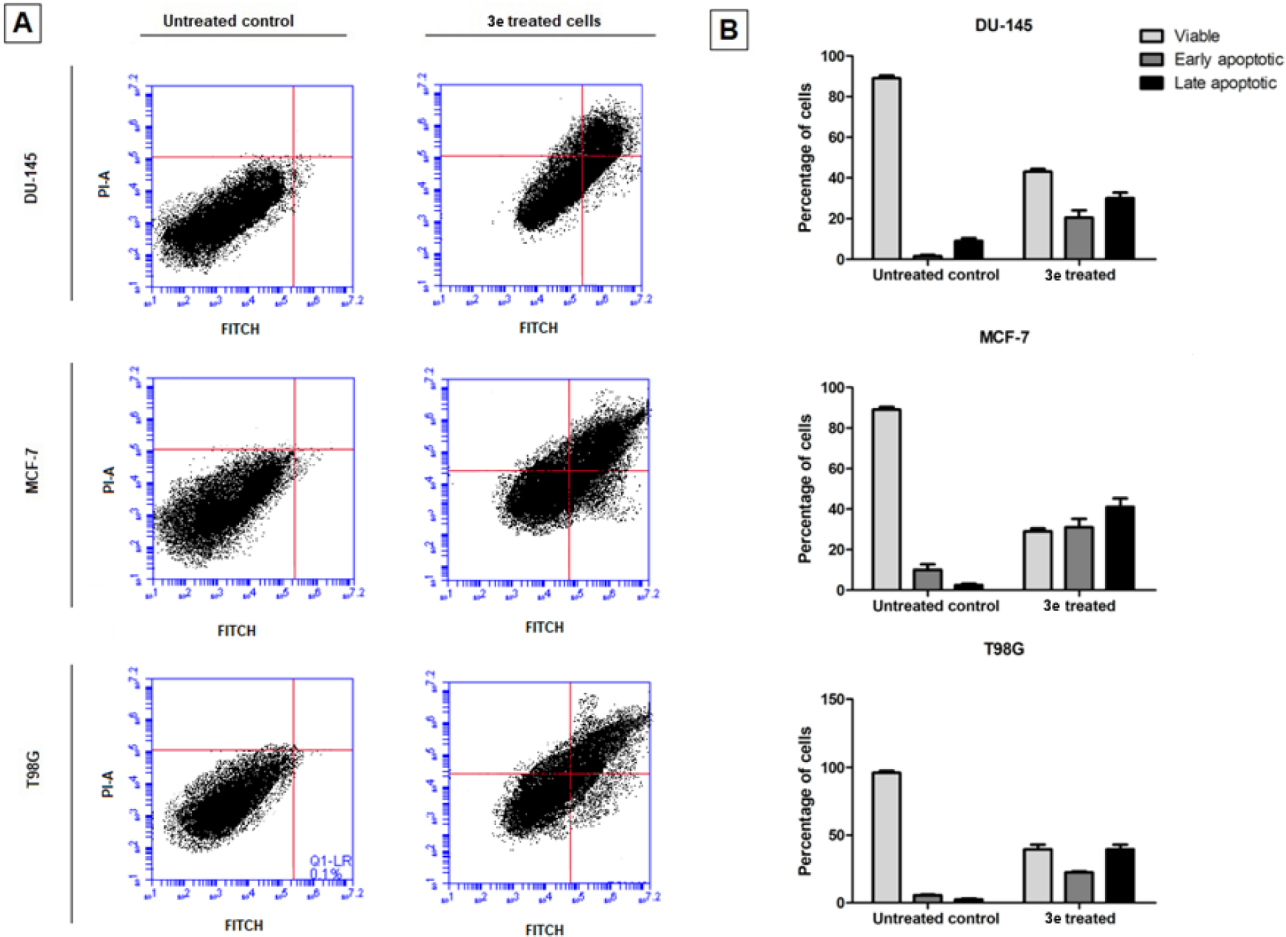
Effects of **3e** and *cis*-platin on apoptosis in MCF-7, DU-145, and T98G cells. (A) Flow cytometric analysis of apoptosis in MCF-7 (human breast cancer), DU-145 (human prostate cancer), and T98G (human glioblastoma carcinoma) cells treated with **3e** and *cis*-platin for 72 h. Cells were stained with Annexin V-FITC and PI to differentiate between viable, early apoptotic, and late apoptotic cells. Untreated control groups were included to establish baseline levels of apoptosis. The scatter plots show the distribution of cells across the four quadrants: viable (Annexin V–/PI−), early apoptotic (Annexin V+/PI−), late apoptotic (Annexin V+/PI+), and necrotic (Annexin V–/PI+). (B) Quantitative analysis of viable, early apoptotic, and late apoptotic cells in MCF-7, DU-145, and T98G cells after 72 h of treatment with **3e** and *cis*-platin. Bar graphs display the percentage of cells in each category for untreated controls, **3e**-treated, and *cis*-platin-treated groups. Both **3e** and *cis*-platin significantly increased early and late apoptotic populations compared to untreated controls (*p* < 0.05). Data represent the mean ± SEM of three independent experiments, and statistical significance was analyzed using one-way ANOVA followed by Tukey’s posthoc test (*p < 0.05 vs untreated control).

Overall, Annexin V/PI flow cytometry analysis revealed that treatment with compound **3e** at the tested concentration and exposure time resulted predominantly in apoptotic cell populations across all cancer cell lines, with minimal necrotic or nonapoptotic death observed (Figure 2). These findings indicate apoptosis as the primary mode of cell death under the experimental conditions employed in this study.

#### 2.2.4 Molecular Docking of **3e** against Key Apoptotic and Cell Cycle Regulatory Proteins

The docking results of **3e** against Bcl-2, Bcl-xL, CDK1, and Cyclin B1 proteins demonstrate strong binding affinities, as reflected by the negative docking scores, which correspond to the binding energy in kcal/mol (Table 3).

**Table 3:** Docking Scores (kcal/mol) of **3e** with the Interacting Residues at the Active Site of Bcl-2, Bcl-xL, CDK1 and Cyclin B1

**Protein Name**	**Docking Score** **(Binding Energy, kcal/mol)**	**H Bond**	**Amino Acid Residue**
Bcl-2	–28.436	-	GLY104, GLU95, GLU111, PHE63, PHE71, PHE112, ASP70, LEU96, TYR67
Bcl-xL	–28.914	–3.467	VAL141, PHE97, PHE191, ALA93, TYR101, TYR195, GLU92, ARG100, ASN136, GLY138, TRP137
CDK1	–27.769	–4.745	GLU163, GLY13, THR161, THR14, GLN132, LYS130, VAL165, THR166, ARG170, ASP128, ARG127, ALA150, PHE153, GLY154, ILE155
Cyclin B1	–36.713	–9.795	MET163, MET172, THR166, LEU137, LEU168, ASP67, ASP169, TYR60, TYR170, ARG38, ARG68, ARG135, GLN21, MET61, VAL23, VAL173, SER64, VAL63, PRO138, PRO176, PRO177, PHE175, ILE180

The docking score of **3e** with Bcl-2 was −118.863 kcal/mol, indicating a strong binding affinity (see Figure 3). However, no specific hydrogen bonds were observed. The binding pocket involved multiple hydrophobic and polar interactions with key residues such as GLY104, GLU95, GLU111, and aromatic interactions with PHE63, PHE71, and PHE112. Additionally, interactions with LEU96, TYR67, and ASP70 suggest stabilization through van der Waals forces, which might play a role in inhibiting the antiapoptotic function of Bcl-2.

**Figure 3:**
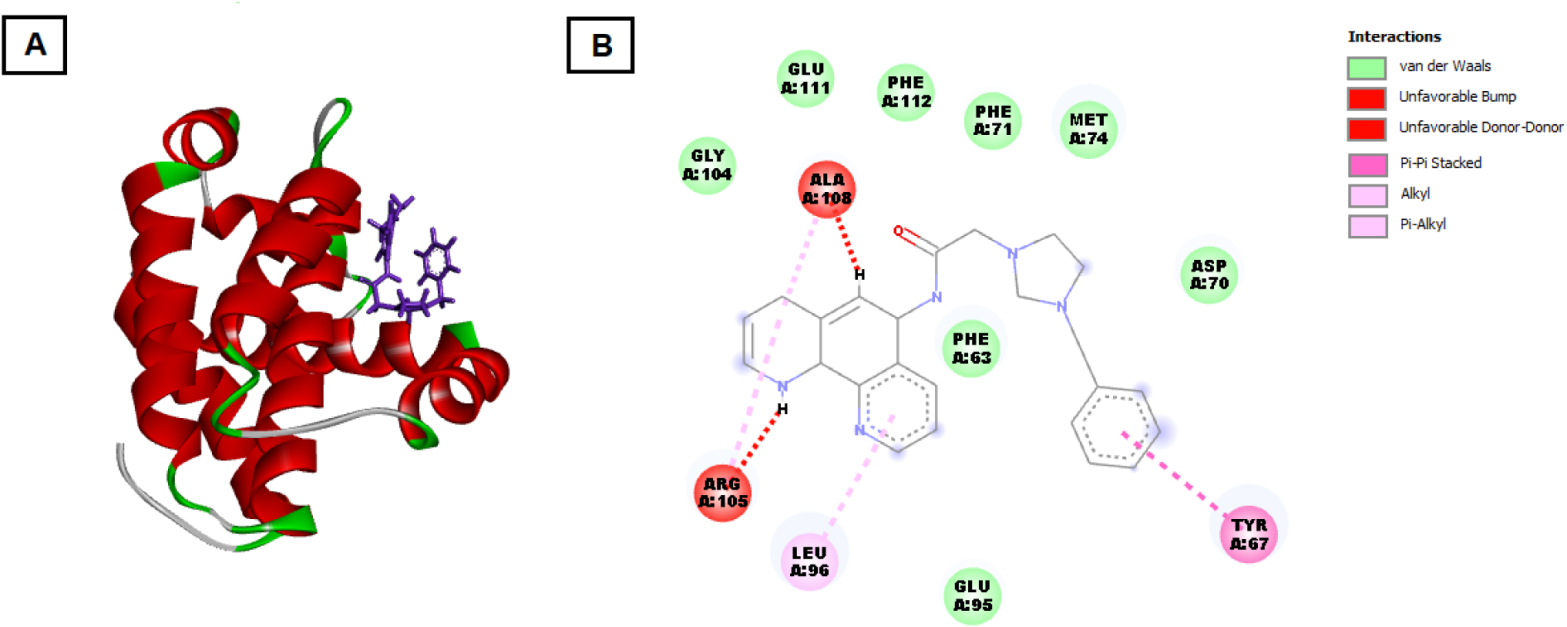
Molecular docking interaction of **3e** with the active site of Bcl-2 protein. (A) is three-dimensional structure of the Bcl-2 protein with **3e** docked into the binding pocket. The protein is represented as a ribbon model, with helices in red, loops in green, and beta strands in gray. The salt **3e** is shown in purple sticks, positioned in the active site, highlighting the spatial arrangement of the compound within the protein’s binding pocket. (B) is detailed two-dimensional interaction diagram between **3e** and key amino acids in the Bcl-2 binding site. Various types of interactions are indicated by colored lines: unfavorable donor–donor interactions are marked in red, van der Waals interactions in green, and π-alkyl interactions in pink. Specific residues that interact with **3e** are labeled and include the following: GLY104, LEU96, GLU95, ASP70, GLU111, PHE112, PHE63, PHE71, and MET74 form van der Waals contacts with the ligand. ARG105 and ALA108 show unfavorable donor–donor interactions, which may contribute to binding instability. TYR67 is involved in a π-alkyl interaction with compound **3e**, which may help stabilize the ligand in the binding site. These interactions collectively contribute to the binding affinity and specificity of **3e** for the Bcl-2 protein.

For Bcl-xL, salt **3e** showed an even higher binding affinity with a docking score of −120.863 kcal/mol (see Figure 4). A significant hydrogen bond was observed with a bond energy of −3.467 kcal/mol, involving amino acid residues like VAL141, PHE97, and PHE191. Additionally, interactions with hydrophobic residues such as ALA93 and aromatic residues like TYR101 and TYR195 indicate strong stabilization of the ligand in the active site. Hydrogen bonding with ARG100 and interactions with ASN136, GLY138, and TRP137 might contribute to the effective inhibition of this antiapoptotic protein.

**Figure 4:**
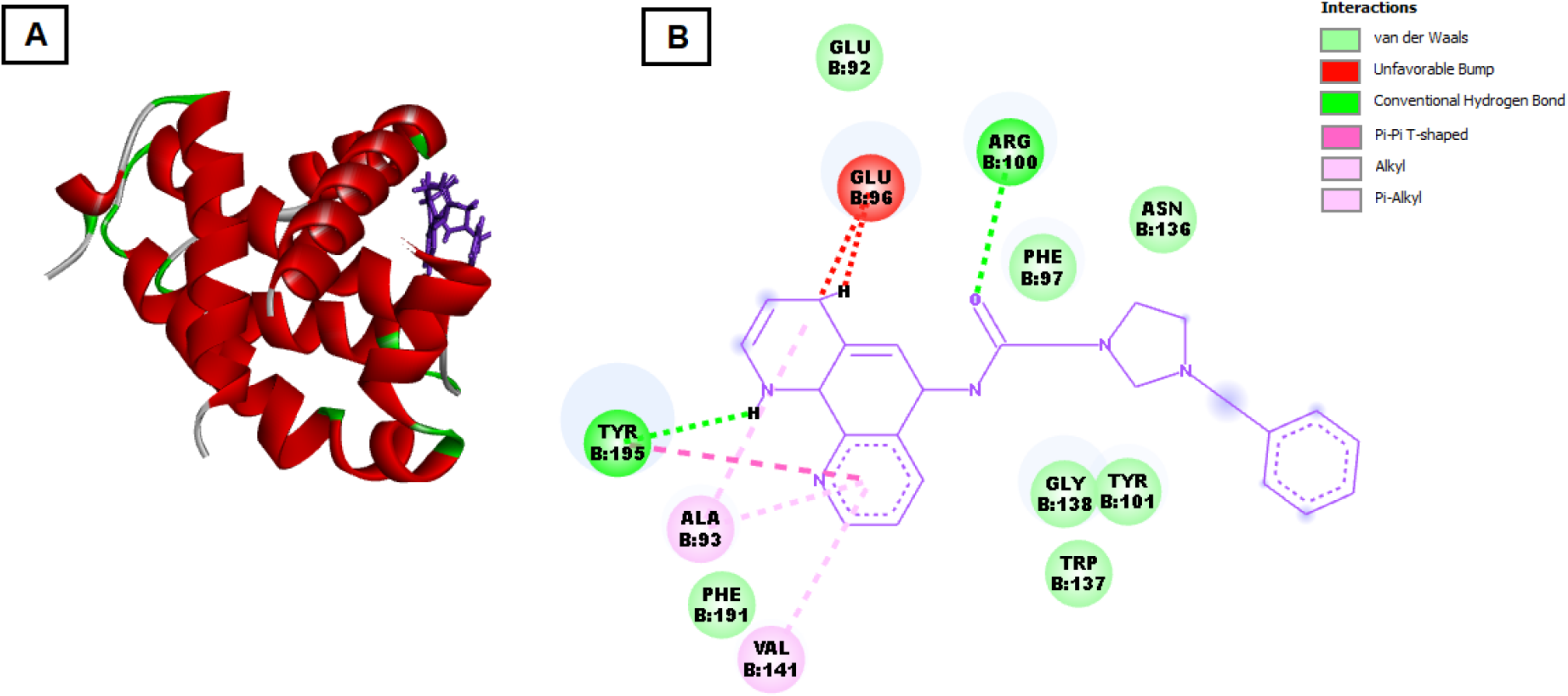
Molecular docking interaction of **3e** with the active site of Bcl-xL protein. (A) is three-dimensional structure of the Bcl-xL protein with **3e** docked in the binding pocket. The protein structure is shown in ribbon form, with helices in red, loops in green, and beta strands (if present) in gray. Compound **3e** is represented as purple sticks in the active site, illustrating its orientation within the binding pocket of Bcl-xL. (B) is two-dimensional interaction diagram between **3e** and key residues in the Bcl-xL binding pocket. The interactions between **3e** and the surrounding amino acids are represented with various colored lines indicating different interaction types: GLU92, GLU96, ARG100, ASN136, PHE97, GLY138, TYR101, TRP137, TYR195, ALA93, and PHE191 participate in van der Waals interactions, shown in green. GLU96 forms a conventional hydrogen bond with **3e**, depicted as a green dashed line. ARG100 shows an unfavorable bump interaction with **3e**, indicated in red. VAL141 and TYR195 engage in π-alkyl interactions with the compound, displayed in light pink. ALA93 and PHE191 participate in π–π T-shaped interactions, marked in magenta. These interactions provide insights into the binding affinity and specificity of **3e** for the Bcl-xL protein, highlighting crucial contacts that stabilize the ligand within the active site.

The interaction of **3e** with CDK1 resulted in a docking score of −116.075 kcal/mol, with the formation of four hydrogen bonds, indicating a substantial binding interaction (see Figure 5). The binding site of CDK1 included crucial residues such as GLU163, GLY13, THR161, THR14, GLN132, LYS130, VAL165, THR166, ARG170, ASP128, ARG127, ALA150, PHE153, GLY154, and ILE155. The presence of residues like GLU163 and ASP128, which are part of the ATP-binding pocket, suggests that **3e** may compete with ATP, potentially inhibiting CDK1 activity. Inhibition of CDK1, a key regulator of cell cycle progression, could lead to cell cycle arrest, particularly in the G2/M phase, thereby limiting cancer cell proliferation.

**Figure 5:**
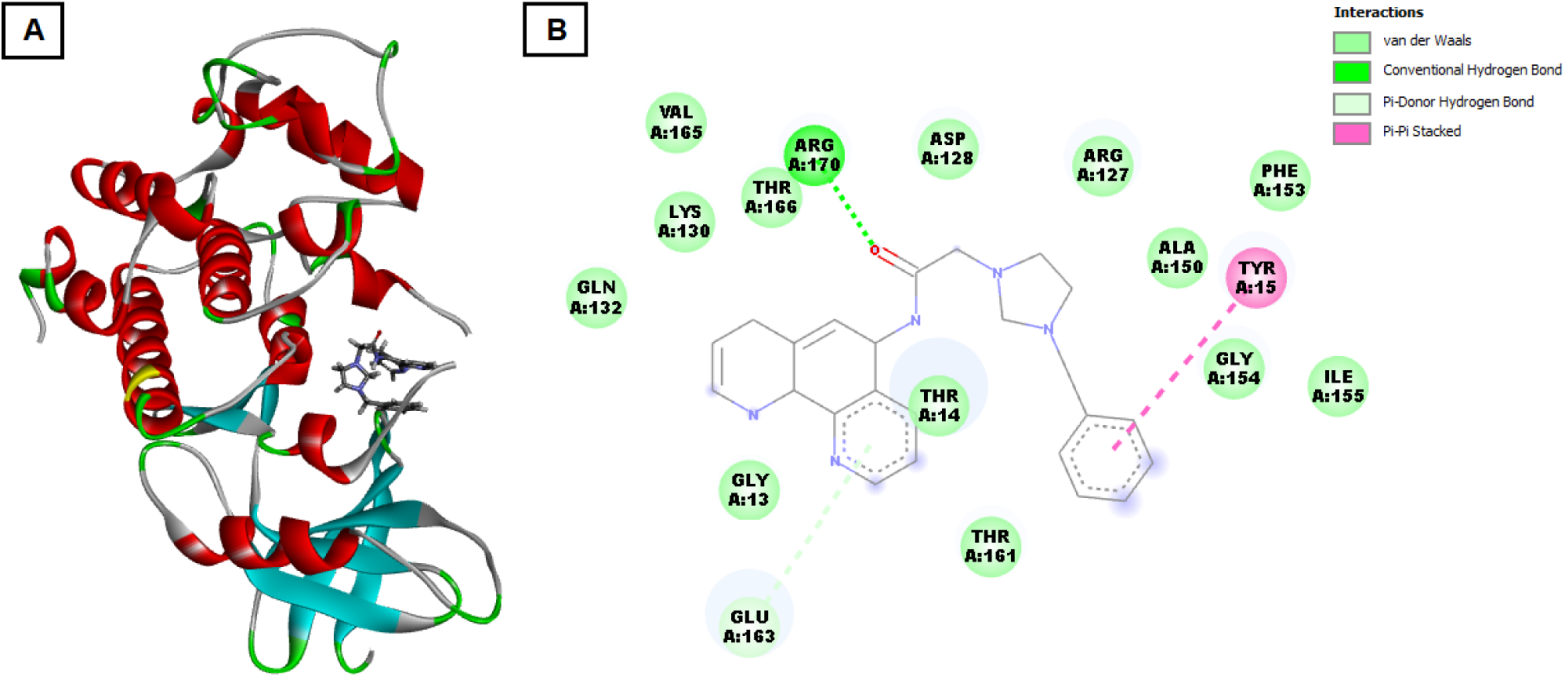
Molecular docking interaction of **3e** with the active site of CDK1 protein. (A) is three-dimensional structure of CDK1 in complex with **3e**. The CDK1 protein is displayed as a ribbon diagram, showing secondary structure elements where α-helices are depicted in red, β-sheets in cyan, and loops in gray. The salt **3e** is represented as a stick model within the CDK1 binding pocket, positioned to show its interactions with the protein. Key residues that interact with the compound are highlighted in different colors to distinguish between various types of interactions. (B) is two-dimensional interaction map of **3e** with the active site of CDK1. This schematic illustrates the specific amino acid residues involved in interactions with **3e** and the nature of these interactions. Each residue is labeled with its amino acid type, position, and chain identifier. The interactions are color-coded: green lines represent van der Waals interactions, green dashed lines denote conventional hydrogen bonds, gray lines signify carbon hydrogen bonds, purple highlights alkyl interactions, and magenta lines indicate covalent bonds. Residues involved in these interactions include LEU A:113, GLY A:115, ILE A:116, HIS A:120, and others, emphasizing the stability and specificity of compound **3e** binding within the CDK1 active site.

Among the tested proteins, salt **3e** exhibited the strongest binding affinity with Cyclin B1, with a docking score of −153.464 kcal/mol and a significant hydrogen bond energy of −9.795 kcal/mol (see Figure 6). The compound showed extensive interactions with a wide range of residues, including MET163, MET172, and THR166, indicating deep binding in the active site. Hydrophobic contacts with LEU137, LEU168, and multiple interactions with ARG residues (ARG38, ARG68, ARG135) were observed, stabilizing the complex. Additionally, aromatic and hydrophobic interactions with TYR60, TYR170, and ILE180, along with hydrogen bonds involving VAL173, SER64, and GLN21, suggest that salt **3e** could effectively disrupt the Cyclin B1-CDK1 complex, potentially inhibiting cell cycle progression in cancer cells.

**Figure 6:**
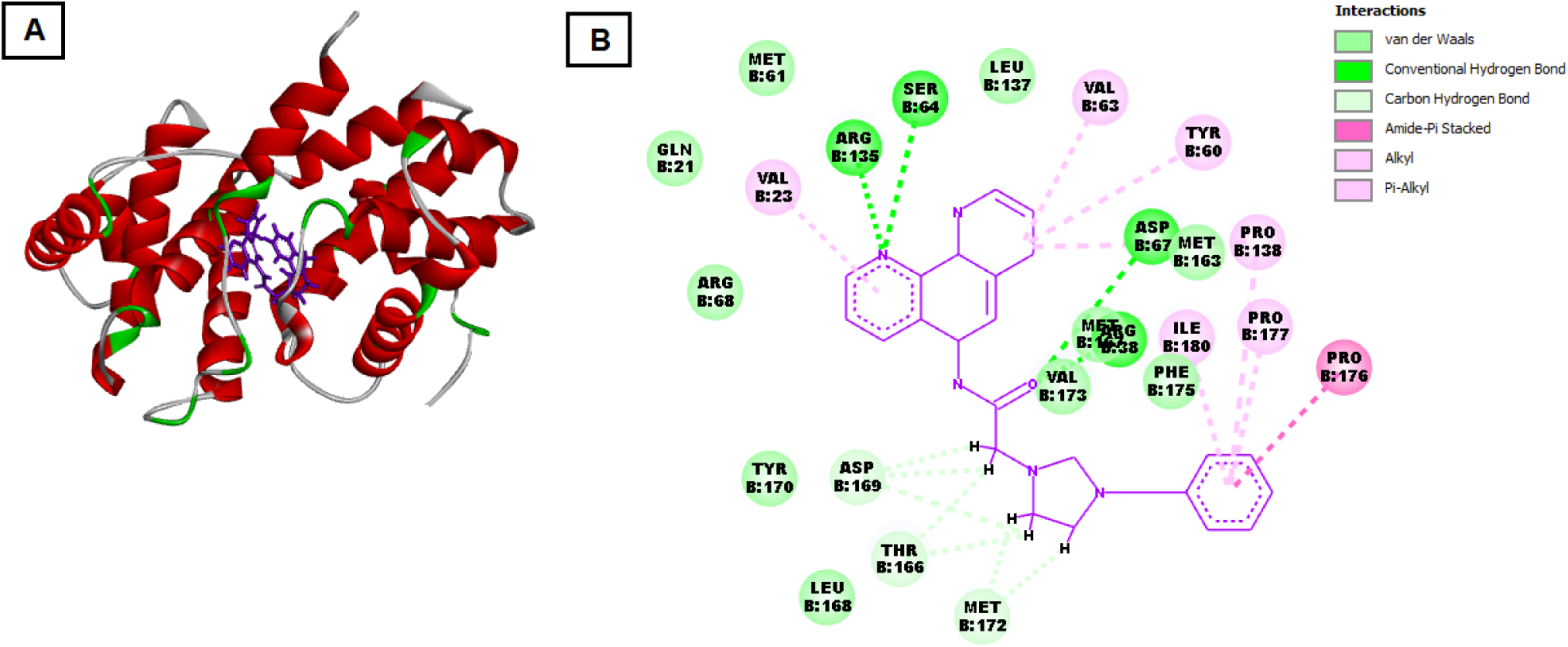
Molecular docking interaction of **3e** with the active site of Cyclin B1 protein. (A) is three-dimensional structure of Cyclin B1 in complex with **3e**. The Cyclin B1 protein is represented as a ribbon diagram, with α-helices displayed in red, β-sheets in cyan, and loop regions in gray. The salt **3e** is shown as a stick model positioned within the binding pocket of Cyclin B1. Key interacting residues are highlighted in different colors to illustrate the various types of interactions. (B) is two-dimensional interaction map of compound **3e** with the active site of Cyclin B1. This schematic shows specific amino acid residues involved in interactions with **3e** and the nature of these interactions. Each residue is labeled with its amino acid type, position, and chain identifier. The interactions are color-coded: green lines represent van der Waals interactions, green dashed lines denote conventional hydrogen bonds, gray lines signify carbon hydrogen bonds, purple dashed lines indicate alkyl and π-alkyl interactions, and additional purple lines represent amide-π stacking interactions. Notable interacting residues include MET B:61, SER B:64, ASP B:67, VAL B:63, PRO B:138, and others, highlighting the binding stability and specificity of **3e** within Cyclin B1’s active site.

The in silico docking analysis revealed that salt **3e** exhibits high binding affinities toward the tested proteins, with the most substantial interactions observed with Cyclin B1. These results suggest that **3e** may act as a multitarget inhibitor, affecting both antiapoptotic pathways (via Bcl-2 and Bcl-xL inhibition) and cell cycle regulation (via CDK1 and Cyclin B1 inhibition).

This multitarget activity could potentially enhance the compound’s efficacy against cancer cells, warranting further in vitro and in vivo validation.

Although direct fluorescence-based visualization of subcellular accumulation was not performed in the present study, multiple lines of functional and computational evidence indicate that the synthesized imidazolium-phenanthroline conjugates gain access to both nuclear and mitochondrial compartments. The pronounced DNA-binding activity, together with molecular docking results demonstrating strong interactions with nuclear cell cycle regulators, suggests functional engagement with nuclear targets. In parallel, the induction of apoptosis confirmed by Annexin V/PI flow cytometry, combined with high-affinity docking toward the antiapoptotic mitochondrial proteins BCL-2 and BCL-XL, supports interference with mitochondrial apoptosis-related pathways. Collectively, these findings support a dual nuclear–mitochondrial mode of action, characterized by functional access to intracellular targets rather than experimentally confirmed physical accumulation. Fluorescence-based subcellular localization studies will be pursued in future work to directly visualize intracellular distribution.

In this context, considerations regarding the cellular entry and membrane transport of the imidazolium-phenanthroline conjugates are warranted. Although direct uptake or membrane transport assays were not performed in the present study, the amphiphilic structural features of these compounds, characterized by the presence of a positively charged imidazolium moiety, a planar aromatic phenanthroline scaffold, and hydrophobic alkyl or benzylic substituents, are consistent with favorable electrostatic interactions with negatively charged phospholipid head groups and passive diffusion or adsorptive uptake across the cell membrane. Importantly, the observed intracellular biological effects, including DNA binding, modulation of antiapoptotic proteins, and apoptosis induction, provide functional evidence supporting effective cellular access to intracellular targets. Quantitative uptake and transporter-specific studies will be addressed in future work.

#### 2.2.5 In Silico ADME Analysis

To evaluate the drug-likeness and pharmacokinetic feasibility of the most potent compound **3e**, in silico ADME analyses were performed based on its SMILES-defined molecular structure. The calculated and predicted parameters are summarized in Table 4. Compound **3e** exhibited a calculated molecular weight of 456.97 g/mol and showed full compliance with Lipinski’s rule of five, indicating a favorable small-molecule drug-like profile. The predicted XlogP3-AA (cLogP) value of 3.42 reflects moderate lipophilicity, which supports efficient membrane permeability while maintaining an acceptable balance with aqueous solubility. The topological polar surface area (TPSA) of 74.6 Å^2^ falls within the optimal range associated with high intestinal absorption and oral bioavailability. Hydrogen bond donor and acceptor counts (HBD = 1, HBA = 6), together with a moderate number of rotatable bonds (n = 6), further support a physicochemical profile compatible with efficient molecular recognition and favorable conformational adaptability for biological interactions. Consistent with these parameters, the compound displayed a predicted bioavailability score of 0.55 and high gastrointestinal absorption, suggesting its suitability for systemic exposure following oral administration. Transport-related predictions indicated that compound **3e** is unlikely to act as a P-glycoprotein substrate, implying a reduced risk of efflux-mediated cellular resistance and favorable intracellular retention. Blood–brain barrier permeability was predicted to be low, indicating limited central nervous system penetration, which may minimize the potential for off-target neurotoxicity. Metabolic liability assessment suggested weak inhibitory potential toward CYP3A4 and a low risk of interaction with CYP2D6, supporting a favorable preliminary drug–drug interaction profile. The compound exhibited moderate predicted microsomal stability and low-to-moderate total clearance, consistent with the potential for sustained systemic exposure under physiological conditions. Toxicity-related predictions further indicated that compound **3e** is nonmutagenic in the Ames model and presents a low risk of hERG channel inhibition, suggesting a favorable early safety profile with respect to genotoxicity and cardiotoxicity. Collectively, the in silico ADME and pharmacokinetic properties of compound **3e** support its nomination as a promising lead candidate for subsequent in vivo biodistribution, pharmacokinetic, and toxicity studies, complementing its experimentally validated anticancer activity and apoptosis-inducing mechanism.

**Table 4:** In Silico ADME and Drug-Likeness Profile of Compound **3e**

**Parameter**	**Calculated/Predicted Value**	**Interpretation**
Molecular weight (g/mol)	456.97	Within small-molecule drug space (<500 g/mol)
XlogP3-AA (cLogP)	3.42	Moderate lipophilicity, favorable for membrane permeability
Topological polar surface area (TPSA, Å^2^)	74.6	Consistent with good intestinal absorption (<90 Å^2^)
Hydrogen bond donors (HBD)	1	Within Lipinski limits
Hydrogen bond acceptors (HBA)	6	Within Lipinski limits
Rotatable bonds	6	Moderate molecular flexibility
Lipinski rule violations	0	Drug-like physicochemical profile
Bioavailability score	0.55	Moderate-to-high predicted oral bioavailability
Gastrointestinal absorption	High (predicted)	Favorable permeability across intestinal epithelium
Blood–brain barrier permeability	Low (predicted)	Limited CNS exposure expected
P-glycoprotein substrate	Unlikely	Reduced efflux liability
Plasma protein binding (%)	∼90–94% (predicted)	High binding consistent with aromatic and cationic character
CYP450 inhibition liability	CYP3A4 (weak), CYP2D6 (low risk)	Low–moderate drug–drug interaction potential
Microsomal stability	Moderate (predicted)	Moderate metabolic susceptibility
Total clearance	Low–moderate (predicted)	Supports reasonable systemic exposure
Ames mutagenicity	Negative (predicted)	Low genotoxicity risk
hERG channel inhibition	Low risk (predicted)	Low cardiotoxicity liability

#### 2.2.6 Stability of the Salt **3e**

For in vitro studies involving organic compounds to yield accurate and reliable results, it is crucial to carefully evaluate solvent and concentration parameters, as well as to investigate the stability of the ligands under specific conditions. Additionally, thorough assessment of the compounds’ stability under the chosen conditions is essential to ensure the validity of the findings. The stability of an organic compound is interpreted by NMR spectroscopy to ensure reliability in the appropriate solvent environment. Therefore, stock solution was prepared in DMSO to study the biological properties of imidazolium salts. One of the key reasons for selecting DMSO as a stock solution is its effectiveness in investigating the biological properties of insoluble drugs. Additionally, DMSO facilitates the replacement of halogen ions bound to the ligand with solvent molecules, which can influence the behavior and reactivity of the ligands. We determined the time-dependent stability of **3e** in a 100% DMSO-*d*_6_ solvent system via ^1^H NMR spectroscopy (see Figure S47). Ligand **3e** was found to remain stable in solution for 6 days. It is an extremely useful method, especially in applications in biochemical analyses or reactions. The salt **3e** was found to remain stable in solution for 6 days and retained its structural integrity, resisting degradation or hydrolysis. Additionally, we investigated the time-dependent stability of **3e** in the solvent system D_2_O/DMSO-*d*_6_ (20:80) via ^1^H NMR spectroscopy (see Figure S48). The NH proton of salt **3e**, which was seen at 11.48 ppm in the instantaneous ^1^H NMR spectrum (see Figure S47), disappeared in the D_2_O/DMSO-*d*_6_ (20:80) solvent system (see Figure S48). The disappearance of the NH proton is a typical observation that can be attributed to the deuterium exchange of the NH proton in D_2_O.

#### 2.2.7 Interaction with FS–DNA

Ultraviolet–visible spectroscopy (UV–vis) method is used to determine the exact binding mode of compounds with FS-DNA. The experiments containing 0.1 mM FS-DNA were performed in 20 mM Tris-HCl/NaCl solution (pH: 7.0). The increase in absorbance with increasing concentrations of **3e** (0–13 μM) suggests that **3e** effectively interacts with FS-DNA, which may indicate intercalation or another binding mechanism. FS-DNA concentration showed maximum absorbance at λ_max_ = 260 nm. The results of the measurement of the maximum absorbance at λ_max_ = 260 nm of the nucleic base chromophores of FS-DNA with a small molecule cause intercalation with a hypochromic effect with a significant shift in the absorption spectra.^30^ The intrinsic binding constant K_*b*_ of the salt **3e** was 1.66 × 10^6^ M^–1^. The K_*b*_ value suggested strong binding of the salt **3e** to FS-DNA (see Figure S49).

#### 2.2.8 Interaction with BSA

In order to study the interaction of the salt **3e** with proteins, bovine serum albumin (BSA) is used as the preferred model protein because it is similar to human serum albumin. Ultraviolet–visible spectroscopy (UV–vis) is used to study how a compound interacts and binds with a protein such as bovine serum albumin (BSA). In the UV range, proteins (aromatic amino acids tryptophan and tyrosine) show maximum absorption at approximately 280 nm (known as λ_*max*_).^31^ The experiments containing 1 μM BSA were performed in PBS buffer at a pH of 7.4. A constant concentration of salt **3e** (15 μM) was prepared in pure water and titrated with varying concentrations of BSA (0–13 μM). The *K*_b_ of **3e** was 23.77 × 10^6^ M^–1^ and is included in the Supporting Information (see Figure S50). As the concentration of the ligand increases, changes in absorption values indicate that the interaction becomes stronger.

#### 2.2.9 Mitochondrial Integrity Following **3e** Treatment

Mitochondrial morphology and integrity were evaluated using Invitrogen MitoTracker dye in cancer cell lines following 72 h exposure to compound **3e** at IC_50_ concentrations (Figure 7). In untreated control groups, DU-145, MCF-7, and T98G cells exhibited strong and homogeneous MitoTracker fluorescence, indicative of intact mitochondrial membrane potential and well-organized mitochondrial networks. The fluorescence signal was diffusely distributed throughout the cytoplasm, consistent with metabolically active cells maintaining functional mitochondria.

In contrast, treatment with compound **3e** resulted in a marked reduction in MitoTracker fluorescence intensity across all three cell lines, suggesting a significant loss of mitochondrial membrane potential. In DU-145 cells, the signal was substantially diminished, with only faint and fragmented fluorescence detectable, indicating pronounced mitochondrial depolarization. Similarly, MCF-7 cells displayed a clear decrease in fluorescence intensity accompanied by disrupted and punctate staining patterns, reflecting mitochondrial fragmentation and dysfunction. The effect was also evident in T98G cells, where mitochondrial staining was severely attenuated, and the remaining signal appeared weak and discontinuous. The observed decrease in mitochondrial staining intensity across all cell lines suggests that compound **3e** exerts its cytotoxic activity, at least in part, through mitochondria-mediated mechanisms, likely involving disruption of mitochondrial membrane potential. Overall, these findings demonstrate that 3e treatment leads to significant mitochondrial dysfunction in DU-145, MCF-7, and T98G cells after 72 h, supporting the hypothesis that mitochondrial impairment contributes to its anticancer activity.

**Figure 7:**
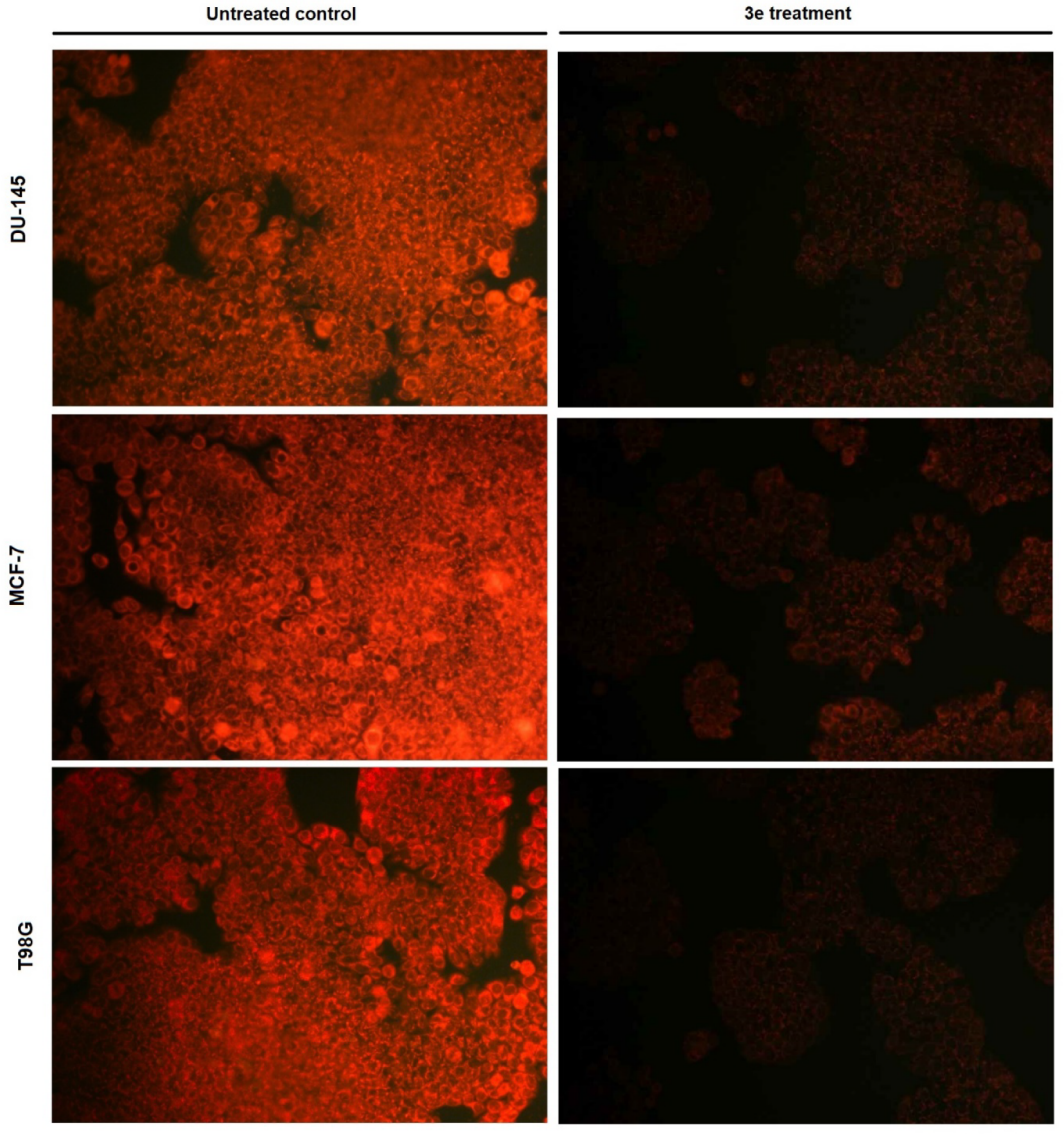
Mitochondrial alterations induced by compound **3e.** DU-145, MCF-7, and T98G cells were treated with compound **3e** (IC_50_, 72 h) and stained with Invitrogen MitoTracker dye. Treated cells showed reduced fluorescence intensity and disrupted mitochondrial staining compared to controls, indicating mitochondrial dysfunction.

## 3 Conclusions

We report the synthesis and characterization of novel 1,10-phenanthroline unfused imidazolium salts **(3a–h)** with different aliphatic or benzylic group and their effects on various cancer cell lines and evaluated their anticancer and DNA-binding properties. The cytotoxicity of **3a–h** was successfully determined in vitro against DU-145 (prostate cancer cells), MCF-7 (breast cancer cells), T98G (glioblastoma cells) and nontumorigenic HEK-293 (human embryonic kidney cells). The synthesized salts **(3a–h)** showed significantly improved anticancer activity. Among the synthesized compounds, **3e** exhibited the highest DNA binding affinity (K_*b*_ = 1.66 ± 0.1 × 10^6^ M^–1^) and potent cytotoxicity against MCF-7 breast cancer cells (IC_50_ = 0.03 ± 0.01 μM). Molecular docking studies confirmed its strong interaction with Bcl-2, Bcl-xL, CDK1, and Cyclin B1, suggesting a dual mechanism of action involving DNA intercalation and apoptotic protein modulation. These results highlight the potential of 1,10-phenanthroline-unfused imidazolium salts as selective anticancer agents that target both DNA and key regulatory proteins, making them promising candidates for further preclinical evaluation. Ongoing and future studies will focus on comprehensive in vivo biodistribution and pharmacokinetic profiling to assess tumor targeting and organ-level distribution, as well as acute and dose-escalation toxicity studies in murine models to establish the safety profile of the lead compound. In parallel, metal-complexed derivatives and structural optimization strategies will be explored to further enhance bioavailability and selectivity.

## 4 Experimental Section

### 4.1 Chemistry

#### 4.1.1 Materials and Reagents

All reagents and solvents used were purchased from commercial sources: UPARC, Merck, Sigma-Aldrich, Riedel-de Haën, and Alfa Aesar. The glassware was heated under vacuum to eliminate oxygen and moisture and then filled with argon. The compounds 5-nitro-1,10-phenanthroline (**1)**, 1,10-phenanthroline-5-amine (**2)**, 2-chloro-*N*-(1,10-phenanthroline-5-yl)­acetamide (**3)** were synthesized using a previously reported method as described in refs 32,33 . Elemental analysis data were used to confirm the successful synthesis of the desired ligands and compare with the mass spectrometry result. ^1^H NMR and ^13^C NMR spectra were obtained using Varian AS 400 Mercury instrument. ^1^H NMR spectra were collected by using DMSO-*d*_6_ as the solvent. Infrared spectral results were recorded using the PerkinElmer 100 series using ATR (Attenuated Total Reflectance), typically covering the range 4000–400 cm^–1^. Melting point data were determined using a Gallenkamp Electrothermal Melting Point apparatus without correction. Chromatographic separation was performed with an HPLC Agilent 1260 Infinity series (Agilent Technologies, Santa Clara, CA, USA) instrument with a Poroshell 120 EC-C18 (3.0 × 50 mm, 2.7 μm particle size) column. The mobile phase system was created as 0.1% formic acid (A) and acetonitrile (B) in water gradient elution: 0–0.5 min, 10% B; 0.5–5 min, 70% B; 5–7 min, 95% B; 7–10 min, 95% B; 10–15 min, 10% B. The column oven was exposed to 35 °C. The injected sample volume was 10 μL and the flow rate used was set at 0.5 mL/min. MS analysis was performed on an Agilent 6550 iFunnel high-resolution Accurate Mass Q-TOF/MS modified with an Agilent Dual Jet Stream electrospray ionization (Dual AJS ESI) interface operating at positive ion: drying gas flow, 14.0 L/min; nebulizer pressure, 35 psi; gas drying temperature, 290 °C; sheath gas temperature, 400 °C; sheath gas flow, nitrogen, 12 L/min. MS/MS spectra were collected with collision energies of 10 eV. The scan range was set from *m*/*z* 50 to 1000. Acquisition was controlled by Agilent MassHunter Acquisition Software Ver. A.09.00 and data were analyzed with MassHunter Qualitative Software Ver. B.07.00. Chromatographic analysis was performed using an Agilent 1260 Infinity II series high-performance liquid chromatography instrument equipped with a diode array detector (DAD) (Agilent Technologies, Santa Clara, CA, USA). Agilent Poroshell 120 EC-C18 HPLC column (5 μm, 4.6 × 150 mm, 225 nm) was used for the separation of analytes from matrices. The mobile phase was a mixture of water and acetonitrile (25:75, v/v). The column oven was kept at 25 °C, the injection volume was determined as 20 μL, and the flow rate was set at 0.5 mL/min.

#### 4.1.2 Synthesis of the 5-Nitro-1,10-phenanthroline **(1)**

The 1,10-phenanthroline monohydrate (10 g, 55.5 mmol) is dissolved in 50 mL of concentrated H_2_SO_4_ and 15 mL of fuming HNO_3_ is added with stirring at 160 °C. The reaction mixture is refluxed for 4 h at a temperature not exceeding 170 °C. Then, after the reaction is cooled, it is poured into ice water and the pH is adjusted to 3 by adding 10 N NaOH solution to the mixture to obtain a yellow solid precipitate. The precipitate is filtered and washed with plenty of water until colorless. The final product 5-nitro-1,10-phenanthroline is dried under vacuum. Yield: 77%, 9.6 g, m.p.: 195 °C. ^1^H NMR 400 MHz, DMSO-*d*_6_: δ 9.25 (d, *J* = 3.6 Hz, 1H, Ar–H), 9.21 (d, *J* = 4.4 Hz, 1H, Ar–H), 8.99 (s, 1H, Ar–H), 8.85 (d, *J* = 8.4 Hz, 1H, Ar–H), 8.74 (d, *J* = 8.0 Hz, 1H, Ar–H), 7.92 (m, 2H, Ar–H). ^13^C NMR (100 MHz, DMSO-*d*_6_): δ 153.7, 151.5, 147.0, 145.7, 144.1, 138.8, 132.3, 126.3, 125.8, 125.8, 124.8, 120.6. IR, υ_max_ (cm^–1^): 3080, 1642, 1687, 1518, 1589, 1506, 1145.

#### 4.1.3 Synthesis of the 1,10-Phenanthrolin-5-amine **(2)**

The 5-nitro-1,10-phenanthroline (5 g, 22.2 mmol) and 10% Pd/C (0.675 g, 6.3 mmol) are dissolved in 50 mL of 95% ethanol solution. The reaction mixture was purged with argon and added hydrazine monohydrate (5 mL, 97.2 mmol) dropwise over 15 min. It is heated for 12H so that the reaction temperature does not exceed 70 °C. At the end of the period, the mixture is filtered hot and the Pd/C catalyst is removed from the media. The yellow filtrate is concentrated to 10 mL and precipitated with diethyl ether. The precipitate is incubated at 4 °C overnight. The precipitate is dried under vacuum to give the final yellow solid. Yield: 80%, (3.46 g), m.p.: 245 °C. ^1^H NMR 400 MHz, DMSO-*d*_6_: δ 9.03 (dd, *J* = 4.0 Hz, 1H, Ar–H), 8.66 (m, 2H, Ar–H), 8.03 (dd, *J* = 8.0 Hz, 1H, Ar–H), 7.71 (dd, *J* = 4.0 Hz, 1H, Ar–H), 7.48 (dd, *J* = 4 Hz, 1H, Ar–H), 6.86 (s, 1H, Ar–H), 6.13 (s, 2H, NH_2_). ^13^C NMR (100 MHz, DMSO-*d*_6_): δ 102.26, 122.30, 122.50, 123.63, 131.03, 131.29, 133.15, 141.04, 143.08, 145.26, 146.66, 149.6. IR, υ_max_ (cm^–1^): 3416, 3322, 3059, 1637, 1611, 1302.

#### 4.1.4 Synthesis of the 2-Chloro-*N*-(1,10-phenanthrolin-5-yl)­acetamide **(3)**

The 1,10-phenanthrolin-5-amine (1 g, 5.12 mmol) and triethylamine (0.85 mL, 6.14 mmol) are dissolved in 200 mL of dichloromethane under an argon atmosphere. 2-Chloroacetyl chloride (0.49 mL, 6.14 mmol) is slowly added to the solution in an ice bath. The reaction mixture is stirred at room temperature for 20 h. Ten mL of distilled water is added to the mixture and dichloromethane is completely distilled under 45 °C. The mixture is stirred at room temperature for 1H. The resulting solid is filtered and washed with plenty of distilled water. A beige solid is obtained. Yield: 84%, 1.17 g, m.p.: 230 °C. ^1^H NMR 400 MHz, DMSO-*d*_6_: δ 10.54 (s, 1H, N*H*), 9.12 (m, 1H, Ar–H), 9.04 (m, 1H, Ar–H), 8.59 (d, *J* = 8.0 Hz, 1H, Ar–H), 8.47 (d, *J* = 8.0 Hz, 1H, Ar–H), 8.17 (s, 1H, Ar–H), 7.82 (m, 1H, Ar–H), 7.74 (m, 1H, Ar–H), 4.51 (d, *J* = 4.0 Hz, 2H, CH_2_). ^13^C NMR (100 MHz, DMSO-*d*_6_): δ 43.80, 121.24, 123.38, 124.07, 125.05, 128.36, 131.49, 131.99, 136.44, 144.44, 146.21, 150.02, 150.40, 166.55. IR, υ_max_ (cm^–1^): 3455, 1681, 1535, 1421, 904, 886, 739, 653.

#### 4.1.5 Synthesis of the 1,10-Phenanthroline Unfused Imidazolium Salts **(3a–h)**

2-Chloro-*N*-(1,10-phenanthrolin-5-yl)­acetamide **(3)** (1 equiv) is taken up in a vacuum gas Schlenk. An excess of *N-*alkylimidazole derivatives are added on top of the solid. The reaction is boiled overnight at 120 °C under reflux. The cooled solution is precipitated with diethyl ether. The final products **(3a–h)** is filtered and dried. Yield **(3a–h)**: 94, 65, 49, 55, 82, 65, 52 and 56%, respectively). mp **(3a–h)**: 150, 134, 130, 87, 178, 245, 315, and 210 °C, respectively.

**3a:** ^1^H NMR 400 MHz, DMSO-*d*_6_: δ 11.36 (s, 1H, NH), 9.31 (s, 1H, N–CH–N), 9.11 (m, 1H, Ar–H), 9.02 (m, 1H, Ar–H), 8.90 (d, *J* = 8.0 Hz, 1H, Ar–H), 8.42 (d, *J* = 8.0 Hz, 1H, Ar–H), 8.15 (s, 1H, Ar–H), 7.87 (s, 1H, N–CH), 7.80 (m, 1H, Ar–H), 7.75 (s, 1H, N–CH), 7.72 (m, 1H, Ar–H), 5.58 (s, 2H, CH_2_), 3.91 (s, 3 H, N–CH_3_). ^13^C NMR (100 MHz, DMSO-*d*_6_): δ 166.90, 150.46, 150.01, 145.16, 144.25, 138.40, 136.41, 132.64, 131.50, 128.31, 124.92, 124.38, 124.13, 123.55, 123.33, 120.68, 51.79, 36.32. IR, υ_max_ (cm^–1^): 3442, 3153, 3109, 2865, 1681, 1535, 1192, 904, 739. Anal. HRMS (ESI) *m*/*z*: [M + H]^+^ calcd for C_18_H_16_N_5_OCl, 354.1116; found 354.1968: C, 60.93; H, 4.83; N, 19.74; O, 4.51%; Found: C, 60.93; H, 4.84; N, 19.80; O, 4.50%.

**3b:** ^1^H NMR 400 MHz, DMSO-*d*_6_: δ 11.39 (s, 1H, N*H*), 9.39 (s, 1H, N–CH–N), 9.14 (d, *J* = 3.6 Hz, 1H, Ar–H), 9.05 (d, *J* = 3.6 Hz, 1H, Ar–H), 8.97 (d, *J* = 4.4 Hz, 1H, Ar–H), 8.50 (d, *J* = 8.0 Hz, 1H, Ar–H), 8.21 (s, 1H, Ar–H), 7.90 (s, 1H, Ar–H), 7.86 (t, *J* = 6.0 Hz, 2H, N–CH), 7.78 (m, 1H, Ar–H), 5.59 (s, 2H, CH_2_), 4.25 (t, *J* = 7.0 Hz, 2H, butyl–CH_2_), 1.78 (m, 2H, butyl–CH_2_), 1.25 (m, 2H, butyl–CH_2_), 0.88 (t, *J* = 7.2 Hz, 3 H, butyl–CH_3_). ^13^C NMR (100 MHz, DMSO-*d*_6_): δ 165.95, 150.34, 149.54, 145.43, 143.35, 137.99, 137.10, 133.07, 131,73, 128.47, 124.96, 124.56, 124.30, 123.54, 122.32, 120.38, 51.92, 49.07, 31.81, 19.16, 13.71. IR, υ_max_ (cm^–1^): 3431, 2913, 2848, 1693, 1469, 1129, 917, 862. Anal. HRMS (ESI) *m*/*z*: [M + H]^+^ calcd for C_21_H_22_N_5_OCl, 396,1586; found 396.1404: C, 63.55; H, 5.84; N, 17.65; O, 4.03%; Found: C, 63.56; H, 5.82; N, 17.66; O, 4.05%.

**3c:** ^1^H NMR 400 MHz, DMSO-*d*_6_: δ 11.41 (s, 1H, NH), 9.38 (s, 1H, N–CH–N), 9.15 (d, *J* = 3.2 Hz, 1H, Ar–H), 9.05 (d, *J* = 6.8 Hz, 1H, Ar–H), 9.0 (d, *J* = 8.4 Hz, 1H, Ar–H), 8.53 (d, *J* = 8.0 Hz, 1H, Ar–H), 8.22 (s, 1H, Ar–H), 7.89 (s, 1H, N–CH), 7.87 (m, 1H, Ar–H), 7.85 (s, 1H, N–CH), 7.80 (m, 1H, Ar–H), 5.59 (s, 2H, CH_2_), 4.23 (t, *J* = 7.2 Hz, 2H, butyl–CH_2_), 1.79 (m, 2H, butyl–CH_2_), 1.22 (m, 10 H, butyl–CH_2_), 0.80 (t, *J* = 6.8 Hz, 3 H, butyl–CH_3_). ^13^C NMR (100 MHz, DMSO-*d*_6_): δ 166.95, 150.29, 149.34, 145.11, 142.95, 137.97, 137.39, 133.26, 131.84, 128.55, 124.98, 124.56, 124.38, 123.64, 122.32, 120.24, 51.94, 49.35, 31.56, 29.84, 28.90, 28.73, 25.87, 22.47, 14.35. IR, υ_max_ (cm^–1^): 3420, 3112, 2848, 1692, 1468, 1169, 912. Anal. HRMS (ESI) *m*/*z*: [M + H]^+^ calcd for C_25_H_30_N_5_OCl, 452.2212; found 452.4309: C, 66.28; H, 6.90; N, 15.46; O, 3.53%; Found: C, 66.30; H, 6.89; N, 15.44; O, 3.54%.

**3d:** ^1^H NMR 400 MHz, DMSO-*d*_6_: δ 11.48 (s, 1H, NH), 9.41 (s, 1H, N–CH–N), 9.15 (d, *J* = 3.6 Hz, 1H, Ar–H), 9.04 (m, 2H, Ar–H), 8.53 (d, *J* = 8.4 Hz, 1H, Ar–H), 8.23 (s, 1H, Ar–H), 7.91 (s, 1H, N–CH), 7.89 (d, *J* = 4.8 Hz, 1H, Ar–H), 7.86 (s, 1H, N–CH), 7.80 (m, 1H, Ar–H), 5.61 (s, 2H, CH_2_), 4.23 (t, *J* = 6.8 Hz, 2H, butyl–CH_2_), 1.78 (m, 2H, butyl–CH_2_), 1.19 (m, 18 H, butyl–CH_2_), 0.79 (t, *J* = 7.2 Hz, 3 H, butyl–CH_3_). ^13^C NMR (100 MHz, DMSO-*d*_6_): δ 166.96, 150.27, 149.26, 144.96, 142.77, 137.97, 137.55, 133.33, 131,89, 128.59, 124.99, 124.56, 124.43, 123.69, 122.31, 120.19, 51.94, 49.35, 31.69, 29.85, 29.45, 29.41, 29.35, 29.27, 29.12, 28.79, 25.88, 22.50, 14.37. IR, υ_max_ (cm^–1^): 3379, 3142, 2922, 2852, 1697, 1550, 1237, 1169, 739. Anal. HRMS (ESI) *m*/*z*: [M + H]^+^ calcd for C_29_H_38_N_5_OCl, 508.2838; found 508.2678: C, 68.42; H, 7.72; Cl, 6.96; N, 13.76; O, 3.14%; Found: C, 68.42; H, 7.74; N, 13.75; O, 3.13%.

**3e:** ^1^H NMR 400 MHz, DMSO-*d*_6_: δ 11.37 (s, 1H, NH), 9.49 (s, 1H, N–CH–N), 9.14 (d, *J* = 3.6 Hz, 1H, Ar–H), 9.05 (d, *J* = 3.2 Hz, 1H, Ar–H), 8.96 (d, *J* = 8.0 Hz, 1H, Ar–H), 8.51 (d, *J* = 7.2 Hz, 1H, Ar–H), 8.20 (s, 1H, Ar–H), 7.91 (s, 1H, N–CH), 7.88 (s, 1H, N–CH), 7.85 (m, 1H, Ar–H), 7.78 (m, 1H, Ar–H), 7.39 (m, 5 H, Ar–H), 5.60 (s, 2H, CH_2_), 5.53 (s, 2H, N–CH_2_). ^13^C NMR (100 MHz, DMSO-*d*_6_): δ 165.93, 150.32, 149.50, 145.35, 143.26, 138.18, 137.16, 135.34, 133.09, 131.73, 129.43, 129.17, 128.71, 128.48, 124.96, 124.92, 124.31, 123.55, 122.45, 120.39, 52.36, 52.06. IR, υ_max_ (cm^–1^): 3398, 2949, 1677, 1542, 1161, 738, 701, 651. Anal. HRMS (ESI) *m*/*z*: [M + H]^+^ calcd for C_24_H_20_N_5_OCl, 430.1429; found 430.1489: C, 66.90; H, 4.91; N, 16.25; O, 3.71%; Found: C, 66.91; H, 4.90; N, 16.24; O, 3.72%.

**3f:** ^1^H NMR 400 MHz, DMSO-*d*_6_: δ 11.12 (s, 1H, NH), 9.12 (d, *J* = 2.8 Hz, 1H, Ar–H), 9.03 (m, 1H, Ar–H), 9.0 (s, 1H, N–CH–N), 8.82 (d, *J* = 8.4 Hz, 1H, Ar–H), 8.44 (m, 1H, Ar–H), 8.14 (s, 1H, Ar–H), 7.87 (s, 1H, N–CH), 7.81 (m, 1H, Ar–H), 7.73 (m, 1H, Ar–H), 7.67 (s, 1H, N–CH), 6.94 (s, 2H, Ar–H), 5.49 (s, 2H, CH_2_), 5.45 (s, 2H, N–CH_2_), 2.24 (d, *J* = 14.8 Hz, 9 H, Ar–CH_3_). ^13^C NMR (100 MHz, DMSO-*d*_6_): δ 165.93, 150.41, 149.88, 146.08, 144.10, 139.02, 138.47, 137.60, 136.51, 132.59, 131.51, 129.81, 128.35, 127.25, 124.87, 124.79, 124.12, 123.30, 122.29, 120.52, 51.95, 47.51, 21.04, 19.69. IR, υ_max_ (cm^–1^): 3358, 2944, 1675, 1548, 1420, 1164, 737, 631. Anal. HRMS (ESI) *m*/*z*: [M + H]^+^ calcd for C_27_H_26_N_5_OCl, 472.1889; found 472.3068: C, 68.56; H, 5.75; N, 14.81; O, 3.38%; Found: C, 68.55; H, 5.77; N, 14.82; O, 3.38%.

**3g:** ^1^H NMR 400 MHz, DMSO-*d*_6_: δ 11.11 (s, 1H, NH), 9.85 (s, 1H, N–CH–N), 9.15 (m, 1H, Ar–H), 9.04 (m, 1H, Ar–H), 8.87 (m, 1H, Ar–H), 8.43 (m, 1H, Ar–H), 8.18 (s, 1H, Ar–H), 8.16 (m, 1H, Ar–H), 8.05 (m, 1H, Ar–H), 7.87 (m, 1H, Ar–H), 7.73 (m, 3 H, Ar–H), 5.84 (s, 2H, CH_2_), 4.17 (s, 3 H, N–CH_3_). ^13^C NMR (100 MHz, DMSO-*d*_6_): δ 165.56, 150.54, 149.94, 144.37, 136.67, 132.44, 132.18, 131.94, 131.41, 128.37, 127.27, 127.0, 124.23, 123.47, 120.59, 114.19, 114.10, 49.51, 33.86. IR, υ_max_ (cm^–1^): 3136, 2979, 2896, 1690, 1553, 1181, 886, 756, 712. Anal. HRMS (ESI) *m*/*z*: [M + H]^+^ calcd for C_22_H_18_N_5_OCl, 404.1273; found 404.4222: C, 65.26; H, 4.73; N, 17.30; O, 3.95%; Found: C, 65.24; H, 4.74; N, 17.31; O, 3.93%.

**3h:** ^1^H NMR 400 MHz, DMSO-*d*_6_: δ 11.74 (s, 1H, NH), 10.37 (s, 1H, N–CH–N), 9.37 (s, 1H, Ar–H), 9.14 (m, 3 H, Ar–H), 8.68 (d, *J* = 7.6 Hz, 1H, Ar–H), 8.28 (s, 1H, Ar–H), 7.93 (m, 2H, Ar–H), 5.76 (s, 2H, CH_2_), 4.16 (s, 3 H, N–CH_3_). ^13^C NMR (100 MHz, DMSO-*d*_6_): δ 166.39, 150.11, 148.43, 145.20, 144.39, 143.27, 139.20, 134.39, 132.30, 128.87, 125.22, 124.91, 124.29, 119.97, 50.44, 20.92. IR, υ_max_ (cm^–1^): 3451, 3408, 3205, 3039, 2931, 1698, 1552, 1231, 1176, 736. Anal. HRMS (ESI) *m*/*z*: [M + H]^+^ calcd for C_17_H_15_N_6_OCl, 355.1069; found 355.2042: C, 57.39; H, 4.53; N, 23.62; O, 4.50%; Found: C, 57.40; H, 4.56; N, 23.60; O, 4.51%.

### 4.2 Pharmacological/Biological Assays

#### 4.2.1 Cell Culture and Cytotoxicity Evaluation

In this study, we utilized both cancer cell lines (DU-145 prostate, MCF-7 breast and T98G glioblastoma) and noncancerous HEK-293 cells. The Interlab Cell Line Collection (Italy) provided the cancer cell lines, while the nontumorigenic HEK-293 cells were sourced from the Health Protection Agency (UK). All cell lines were cultured in RPMI medium (Sigma) supplemented with 10% heat-inactivated fetal bovine serum, 1% penicillin, and 1% l-glutamine. Incubation was carried out at 37 °C in a humidified atmosphere with 5% CO_2_.

To evaluate the cytotoxicity of the synthesized salts, which were dissolved in dimethyl sulfoxide (DMSO), the XTT assay (2,3-Bis­(2-methoxy-4-nitro-5-sulfophenyl)-2*H*-tetrazolium-5-carboxanilide) was conducted. Cells were seeded in 96-well plates at a density of 10^4^ cells per well and exposed to varying concentrations of salts **3a–h** (1–250 μM) for 24, 48, and 72 h. Following incubation, 100 μL of XTT solution was added to each well, and the plates were further incubated for 4 h at 37 °C. Absorbance was measured at 570 nm using a Tecan microplate reader. IC_50_ values, indicating the concentration required to inhibit 50% of cell proliferation, were determined using Biosoft CalcuSyn 2.1 software.

To evaluate the selectivity of the salts for cancer cells over normal cells (HEK-293), the selectivity index (SI) was determined using the formula:

$$\mathrm{Selectivity}\;\mathrm{Index}\;\mathrm{(SI)}={\mathrm{IC}}_{50}\;\mathrm{for}\;\mathrm{healthy}\;{\mathrm{cells/IC}}_{50}\;\mathrm{for}\;\mathrm{cancer}\;\mathrm{cells}$$

An SI greater than 1 indicates greater toxicity toward cancer cells, while an SI equal to 1 suggests similar toxicity toward cancerous and normal cells. If the SI is less than 1, the compound is more toxic to normal cells, which is undesirable in potential therapies. Based on literature criteria, an SI value of 2 or greater signifies meaningful selectivity, suggesting that the compound preferentially targets cancer cells over normal cells.^34^

#### 4.2.2 Assessment of Cell Cycle Phases via Flow Cytometry

To analyze the distribution of cells across different cell cycle phases, DNA fragments stained with propidium iodide (PI) were examined using the Cell Cycle Phase Determination Kit (Cayman Chemical, USA). Cells were seeded in six-well plates at a density of 1 × 10^6^ cells per well in 2 mL of culture medium and incubated at 37 °C in a CO_2_ incubator for 72 h. Following incubation, cells were treated with the IC_50_ concentration of **3e** for an additional 72 h. After treatment, cells were collected by centrifugation and washed twice with cold phosphate-buffered saline (PBS). The cell pellets were then fixed and permeabilized by adding 1 mL of fixative and incubating for 2 h. After a second centrifugation, the fixative was removed, and the cells were resuspended in 0.5 mL of staining solution containing 200 μL of DNase-free RNase (Sigma-Aldrich Co) and PI. The suspension was incubated in the dark at room temperature for 30 min. Finally, cell cycle phase distribution was analyzed using an Accuri C6 flow cytometer (BD Biosciences, USA).

#### 4.2.3 Apoptosis Detection

Annexin V, a protein with high affinity for phosphatidylserine (PS), is widely used for apoptosis detection. In healthy cells, PS is confined to the inner leaflet of the plasma membrane; however, during apoptosis, it relocates to the outer leaflet, serving as a crucial marker of apoptotic cells.^35^ Annexin V specifically binds to phosphatidylserine (PS) on the surface of apoptotic cells, while propidium iodide (PI), a secondary dye, is used to distinguish between apoptotic and viable cells. This allows for the identification of live cells (Annexin V–, PI−) and apoptotic cells (Annexin V+, PI+). For this study, the FITC Annexin V Apoptosis Detection Kit I (BD Pharmingen) was utilized. Cancer cells were seeded at a density of 1 × 10^6^ cells per well in 6-well plates and treated with the IC_50_ concentration of **3e** for 72 h. Following treatment, cells were washed with cold PBS, resuspended in 1 mL of 1× Binding Buffer, and stained with 5 μL of Annexin V FITC and 5 μL of PI. The samples were vortexed and incubated in the dark at room temperature (25 °C) for 15 min. After incubation, 400 μL of 1× Binding Buffer was added, and apoptosis was assessed using a BD Accuri C6 Flow Cytometer.

#### 4.2.4 Molecular Docking Studies

Molecular docking studies for the salt **3e** were performed to investigate its interactions with key proteins involved in apoptosis inhibition and cell cycle arrest at the G2/M phase. AutoDock Vina version 1.1.2 was utilized for the docking simulations. The X-ray crystallographic structures of the target proteins were downloaded in PDB format from the RCSB Protein Data Bank (https://www.rcsb.org/). These proteins included Bcl-2 (PDB ID: 2W3L), Bcl-xL (PDB ID: 2YXJ), CDK1 (PDB ID: 6GU6), and Cyclin B1 (PDB ID: 2B9R).

Protein preparation was carried out using the Protein Preparation Wizard, which assigned bond orders, added hydrogen atoms, processed metal ions, and removed water molecules. The proteins were then energy-minimized with a root-mean-square deviation (RMSD) of 0.30 Å to optimize them for docking. The 3D structures of the ligands were generated using Maestro 8.5 software from the Schrödinger suite, while Open Babel was employed to generate the 3D structures of the synthesized compounds. The docking grid box parameters were adjusted to ensure an RMSD below 2 Å, with the grid center coordinates for EGFRWT set at X = 21.41, Y = 3.62, and Z = 21.94, and grid dimensions set to 60 Å × 60 Å × 60 Å. These parameters were applied to all protein targets following calibration. The docking results provided various conformations, which were further analyzed using Discovery Studio to explore the secondary structures. *Cis*-platin (CP) was used as a reference compound.

The chemical structure of **3e** was optimized using Gaussian 09W and saved in SDF format, which was then imported into the Maestro graphical user interface (GUI). Ligand preparation was performed with the default parameters in the LigPrep module, using the OPLS 2005 force field at physiological pH. After preparation, all ligand conformations were docked into the receptor grid with a 20 Å radius. Co-crystallized ligands were used to identify the active binding site, and redocking these ligands resulted in RMSD values below 2 Å, confirming the reliability and accuracy of the docking protocol.

#### 4.2.5 In Silico ADME and Pharmacokinetic Analysis

The pharmacokinetic and drug-likeness properties of the most potent compound **3e** were evaluated using using the ADMET 2.0 tool. The molecular structure of compound **3e** was defined by its SMILES notation, and all calculations were performed on the cationic form of the molecule, excluding the counterion, in accordance with standard ADME modeling practices. Physicochemical descriptors, including molecular weight, XlogP3-AA (cLogP), topological polar surface area (TPSA), hydrogen bond donors (HBD), hydrogen bond acceptors (HBA), and the number of rotatable bonds, were calculated using QSAR-based descriptor models. Drug-likeness was assessed according to Lipinski’s rule of five and an estimated oral bioavailability score. Pharmacokinetic properties, including gastrointestinal absorption, blood–brain barrier permeability, and P-glycoprotein substrate likelihood, were predicted using validated classification models trained on reference data sets of approved small-molecule drugs. Metabolic liability was evaluated through prediction of cytochrome P450 isoform inhibition (CYP3A4 and CYP2D6), microsomal stability, and total clearance. All predicted parameters were used to generate an integrated ADME and pharmacokinetic profile for compound **3e**, which was subsequently correlated with its experimentally observed cytotoxicity and apoptosis-inducing activity.

#### 4.2.6 DNA Binding

DNA binding experiments of ligand **3e** were evaluated using UV–vis spectroscopy to 0.1 mM Fish Sperm DNA (FS-DNA) in Tris-HCl buffer (20 mM Tris-HCl/NaCl, pH 7.0). The Benesi–Hildebrand equation was used to analyze the binding of ligand to a receptor and the intrinsic binding constant (K_*b*_) based on spectroscopic measurements.

The general form of the Benesi–Hildebrand equation is

$$1/\left(A-A_{0}\right)=1/\left\{K_{b}\left(A_{\max}-A_{0}\right)\left[Q\right]\right\}+1/\left[A_{max}-A_{0}\right]$$

Where:

- A_0_ is the absorbance or the observed signal at a given concentration of ligand (A),
- *A*_max_ is the maximum absorbance (when the receptor is fully bound by the ligand),
- A is the concentration of the free ligand,
- [Q]: concentration of ligand available for binding,
- K_*b*_ is the intrinsic binding constant, which you are looking to calculate; 1/ [A – A_0_] versus 1/[Q].

#### 4.2.7 Bovine Serum Albumin (BSA) Binding Assay

Protein-molecule interaction was investigated by BSA binding test using Ultraviolet–visible spectroscopy (UV–vis). For this purpose, 1 μM BSA solution was prepared in 0.01 M phosphate buffered saline (pH 7.4). The fixed BSA concentration was titrated with varying concentrations (0–13 μM) of the **3e** molecule. The Benesi–Hildebrand equation was used to analyze the binding of ligand to a receptor and the intrinsic binding constant (K_*b*_) based on spectroscopic measurements.

#### 4.2.8 Lipophilicity

This passage discusses two methods for predicting the log*P* (partition coefficient) of chemical compounds, focusing on their accuracy and methodologies. First, the ALOGPS computational model uses artificial intelligence with data from 12,908 compounds originating from the PHYSPROP database. Developed 64 neural networks, with 50% of the data set randomly selected for training. It was stated that the developed artificial intelligence had a root-mean-square error (RMSE) of 0.35 and a mean error(s) margin of 0.26. This indicates a high level of precision in log*P* predictions.

#### 4.2.9 Statistical Analysis

Data analysis was carried out using GraphPad Prism 5.0 software. To evaluate statistical differences, a one-way ANOVA was initially performed, followed by Tukey’s posthoc test for multiple comparisons. A significance level of *p* < 0.05 was used to determine statistical relevance. Results are expressed as the mean ± standard deviation (SD).

#### 4.2.10 Mitochondrial Staining of Cells Using MitoTracker Dye

Mitochondrial staining in adherent cells was performed using a commercial mitochondria dye (Invitrogen MitoTracker, M7512) in accordance with the manufacturer’s instructions. Cells were cultured under appropriate conditions until reaching 80% confluency and subjected to IC_50_ value of 3e for 72 h. The MitoTracker stock solution was prepared in DMSO and diluted to a final working concentration of 200 nM in phenol red-free, serum-free medium. After removal of the culture medium, the staining solution was added to the cells, followed by incubation at 37 °C in the dark for 30 min. Subsequently, the staining solution was removed and replaced with fresh prewarmed medium. Stained cells were then visualized using a fluorescence microscope (Olympus) at 579/599 nm, and mitochondrial localization was assessed.

## Supplementary Material



## Notes

^1^H and ^13^C NMR spectra of **1**, **2**, **3**, **3a–h**; FTIR spectra of **1, 2, 3, 3a–h**; HRMS analysis of **3a–h**; HPLC analysis of **3e**; Cell viability results of the **3a–h**; ^1^H NMR stability of **3e** in DMSO-*d*_6_ over 6 days; ^1^H NMR stability of **3e** in 20% D_2_O/DMSO-*d*_6_ over 6 days; FS-DNA structure analysis of **3e**; UV–vis spectra of BSA with **3e** (PDF)
