# Supplementary material for: Synthesis and Characterization of 1,10-Phenanthroline-Linked Imidazolium Salts: Investigation of Their Cytotoxicity Properties
Source: ACS Omega. 2026 Apr 28;11(18):26736–50. doi: 10.1021/acsomega.5c13398 (PMC13177221; doi:10.1021/acsomega.5c13398)
Supplement: Supplementary file 1 [file ao5c13398_si_001.pdf]

Supporting Information

Synthesis and characterization of 1,10-phenanthroline-linked imidazolium salts: investigation of their cytotoxicity properties

Aslıhan Karaer Tunçay,[1] Suleyman Ilhan,[2] Harika Atmaca[2] and Hayati Türkmen,[1]*

[1]Department of Chemistry, Faculty of Science, Ege University, Bornova, 35100 Izmir, Türkiye

[2]Department of Biology, Faculty of Engineering and Natural Sciences, Manisa Celal Bayar University, Manisa, Türkiye

Contents

^{1}H and ^{13}C NMR spectra of **1**, **2**, **3**, **3a–h**

FTIR spectra of **1**, **2**, **3**, **3a–h**

HRMS analysis of **3a–h**

HPLC analysis of **3e**

Cell viability results of the **3a–h**

^{1}H NMR stability of **3e** in DMSO-d_6 over 6 days.

^{1}H NMR stability of **3e** in 20 % D_2O/DMSO-d_6 over 6 days.

FS-DNA structure analysis of **3e**

UV-Vis spectra of BSA with **3e**

^{1}H-NMR spectra of 1

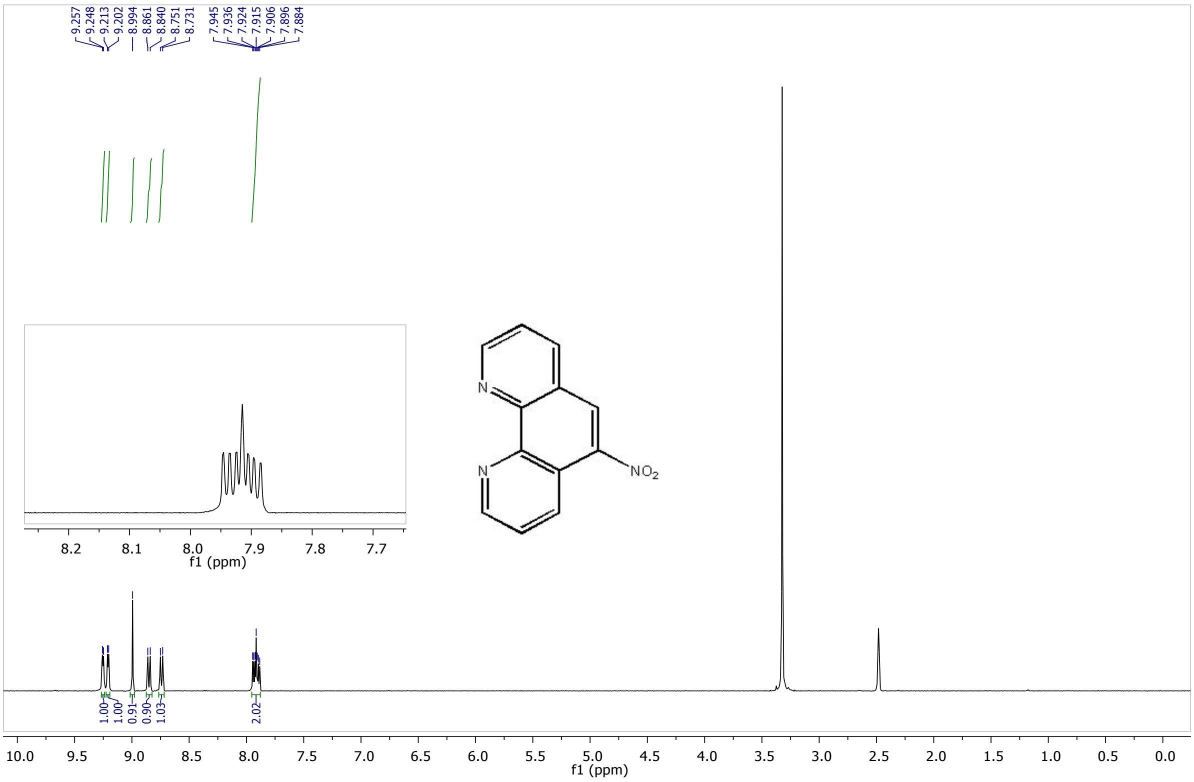

Figure S1. ^{1}H-NMR spectrum of **1** (DMSO-d_6).

^{13}C-NMR spectra of 1

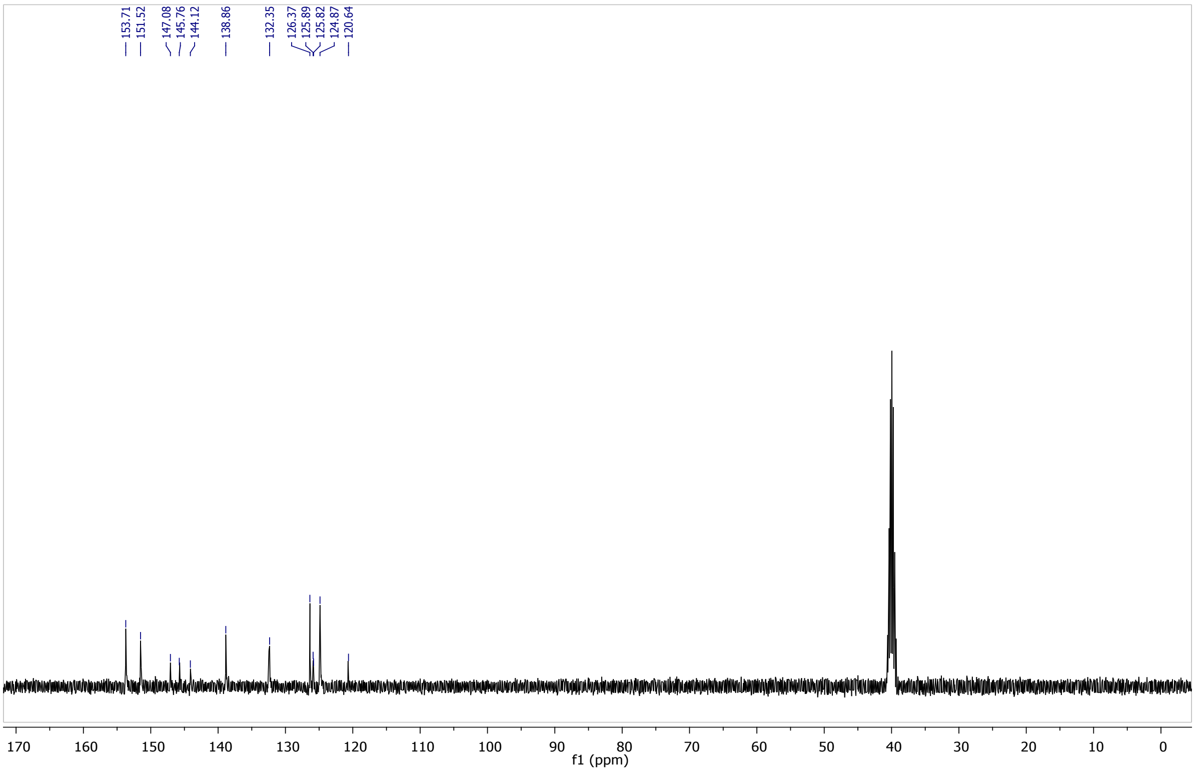

Figure S2. ^{13}C-NMR spectrum of **1** (DMSO-d_6).

^{1}H-NMR spectra of 2

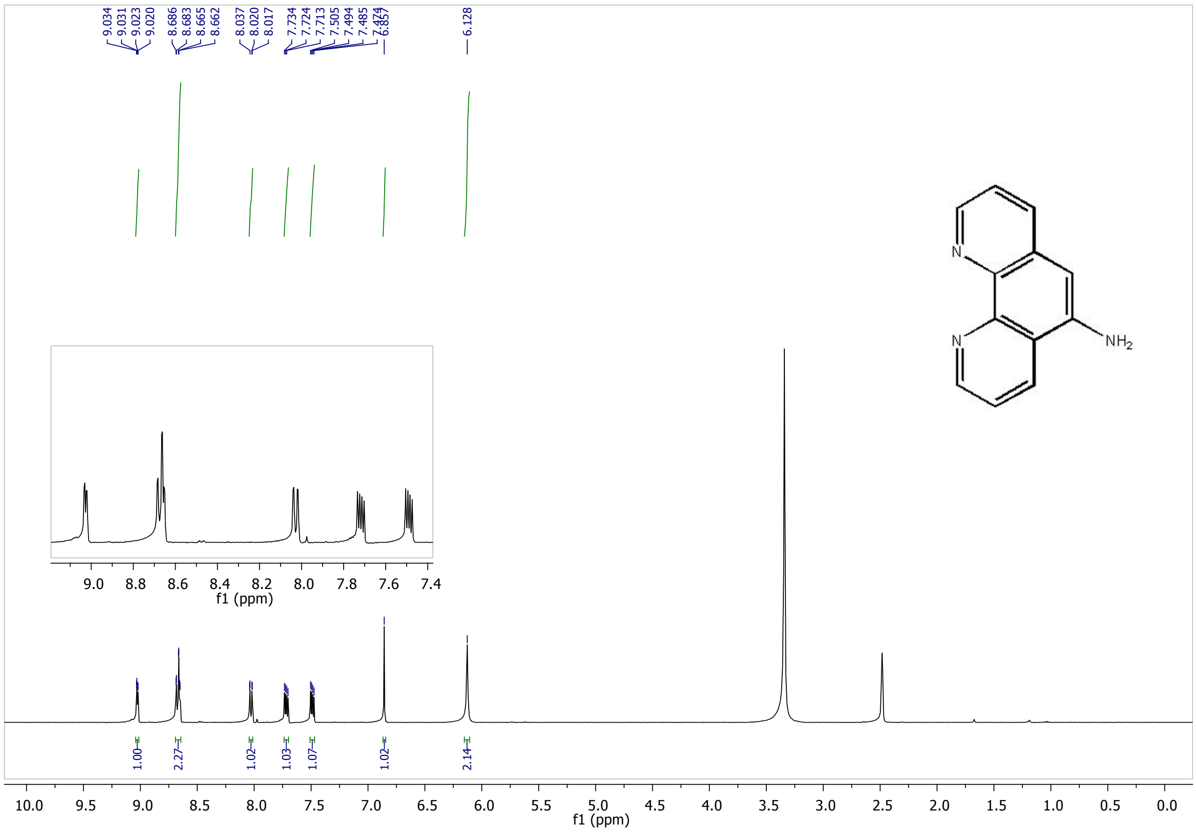

Figure S3. ^{1}H-NMR spectrum of **2** (DMSO-d_6).

^{13}C-NMR spectra of 2

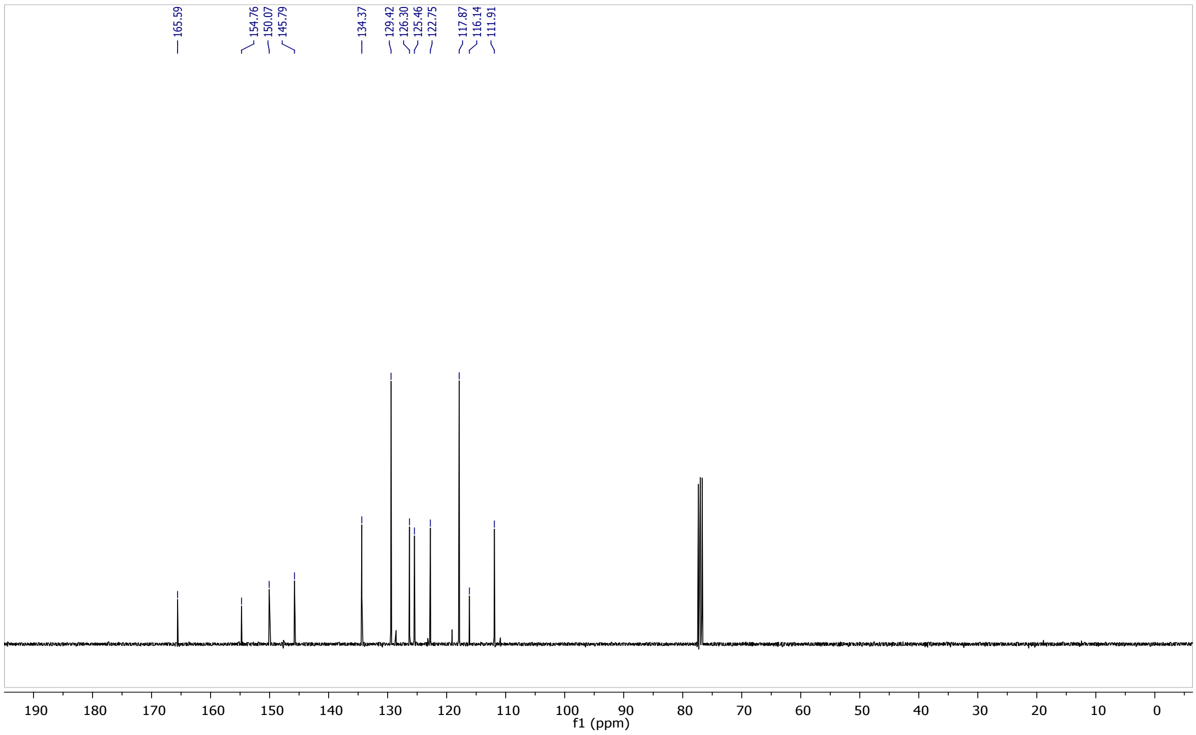

Figure S4. ^{13}C-NMR spectrum of **2** (DMSO-d_6).

^{1}H-NMR spectra of 3

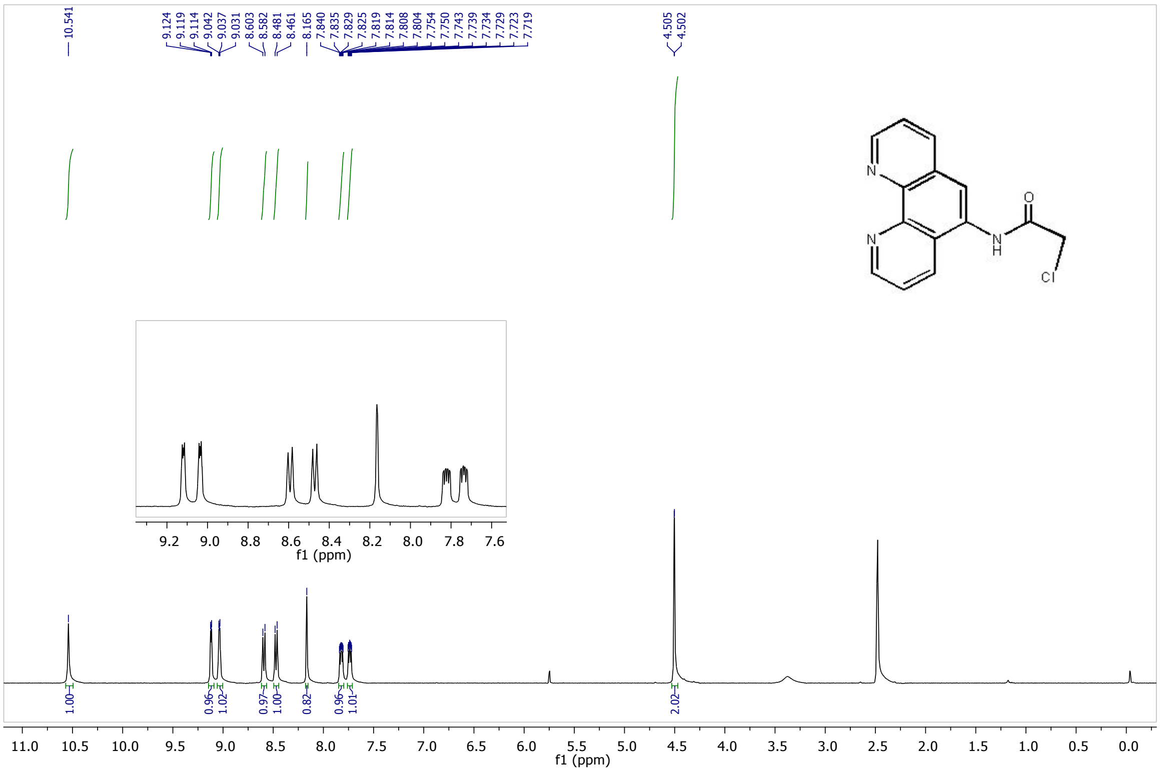

Figure S5. ^{1}H-NMR spectrum of **3** (DMSO-d_6).

^{13}C-NMR spectra of 3

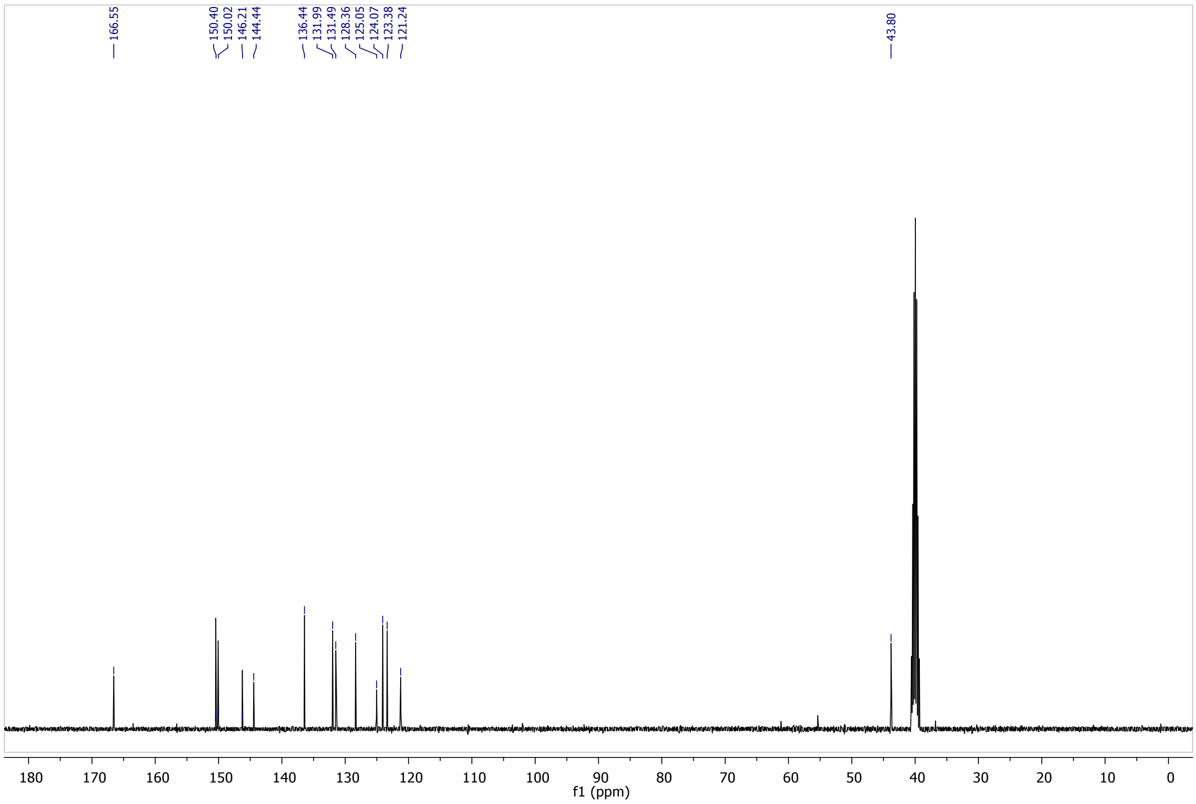

Figure S6. ^{13}C-NMR spectrum of **3** (DMSO-d_6).

^{1}H-NMR spectra of 3a

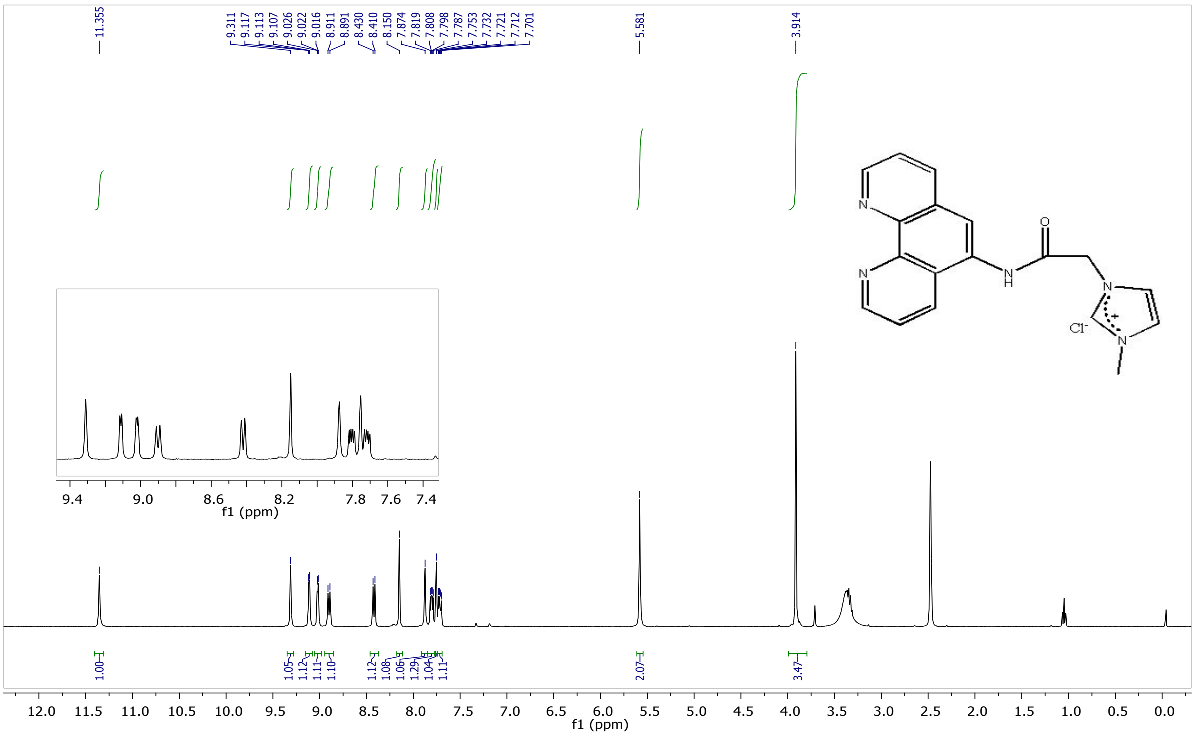

Figure S7. ^{1}H-NMR spectrum of **3a** (DMSO-d_6).

^{13}C-NMR spectra of 3a

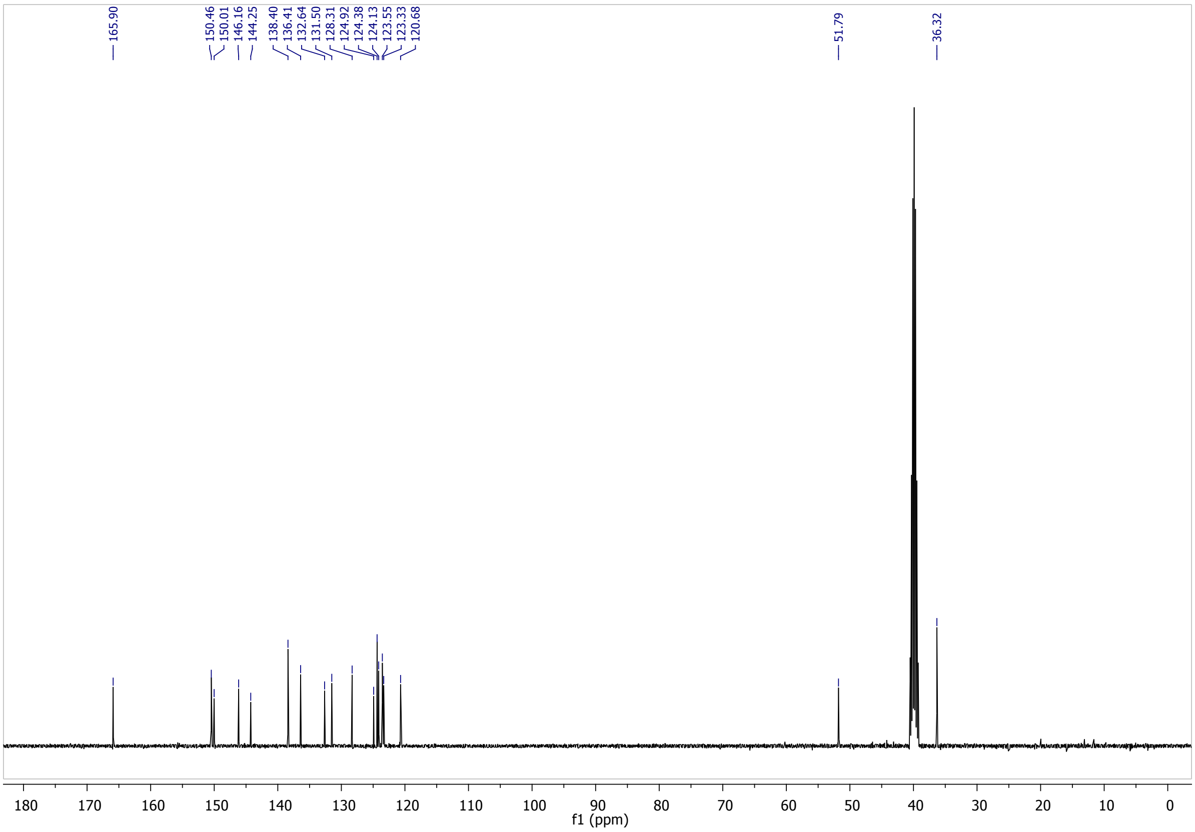

Figure S8. ^{13}C-NMR spectrum of **3a** (DMSO-d_6).

^{1}H-NMR spectra of 3b

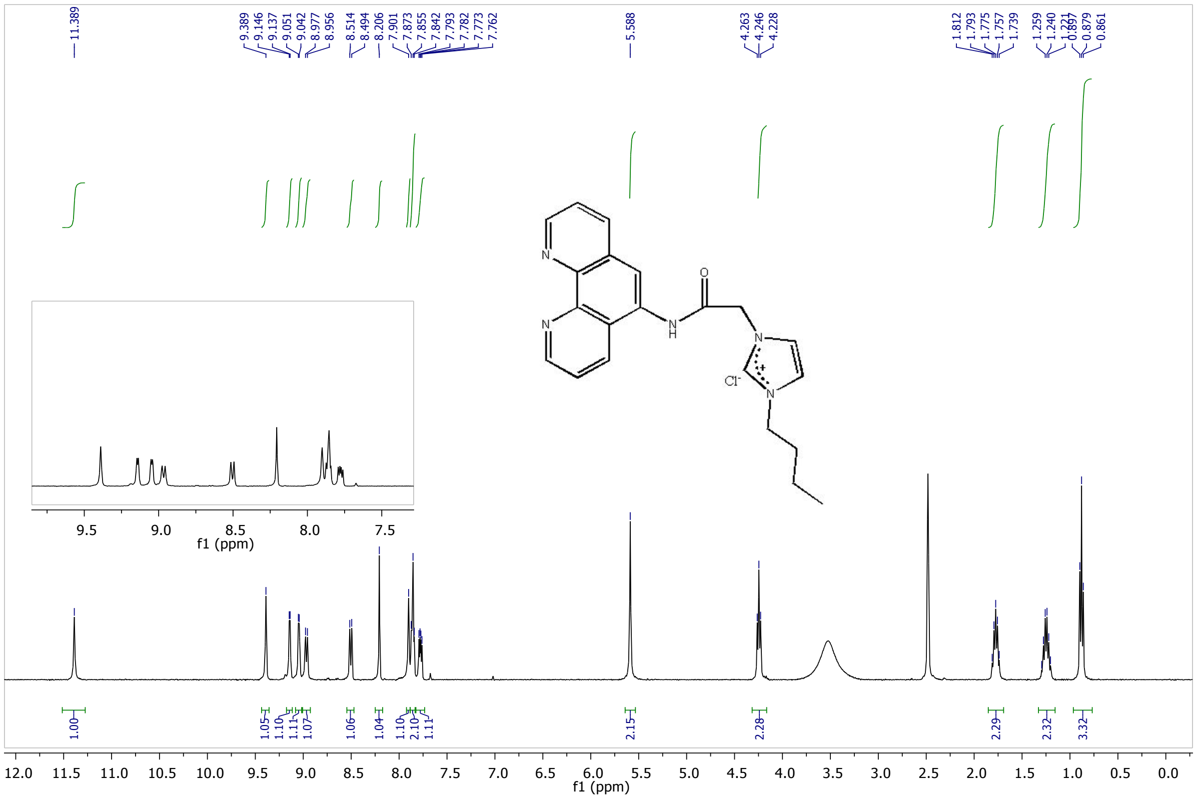

Figure S9. ^{1}H-NMR spectrum of **3b** (DMSO-d_6).

^{13}C-NMR spectra of 3b

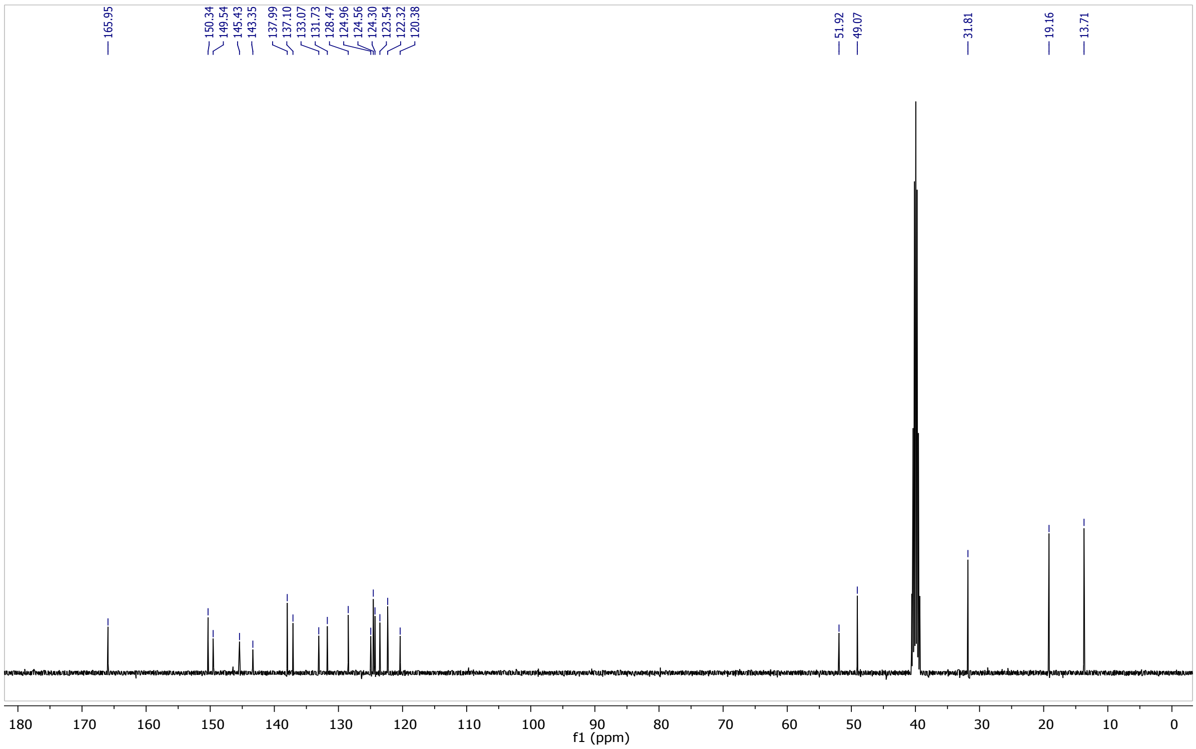

Figure S10. ^{13}C-NMR spectra of **3b** (DMSO-d_6).

^{1}H-NMR spectra of 3c

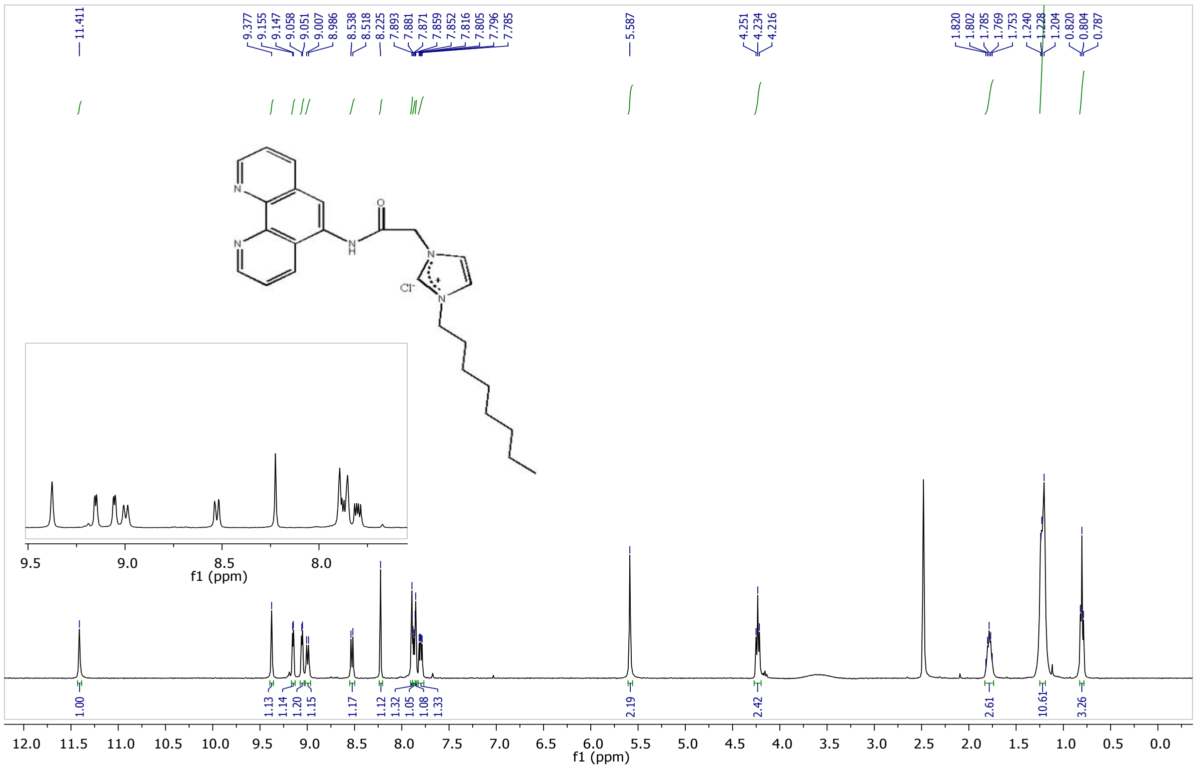

Figure S11. ^{1}H-NMR spectrum of **3c** (DMSO-d_6).

^{13}C-NMR spectra of 3c

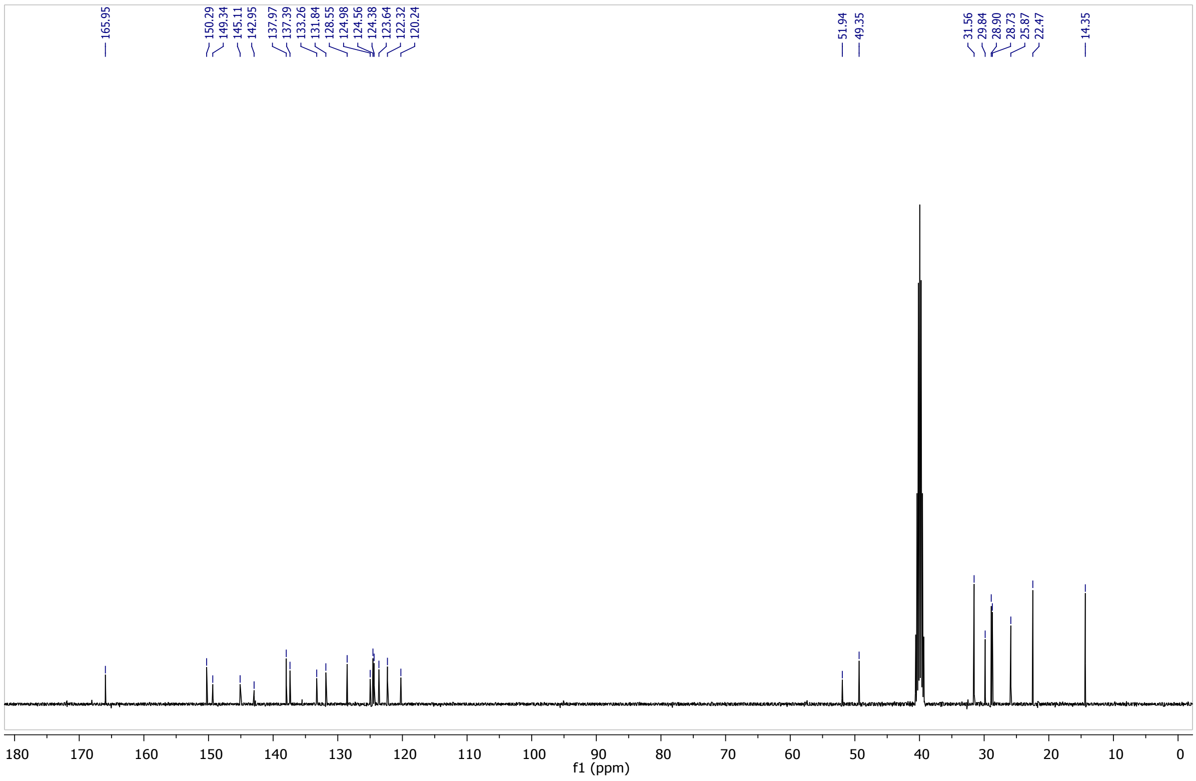

Figure S12. ^{13}C-NMR spectra of **3c** (DMSO-d_6).

^{1}H-NMR spectra of 3d

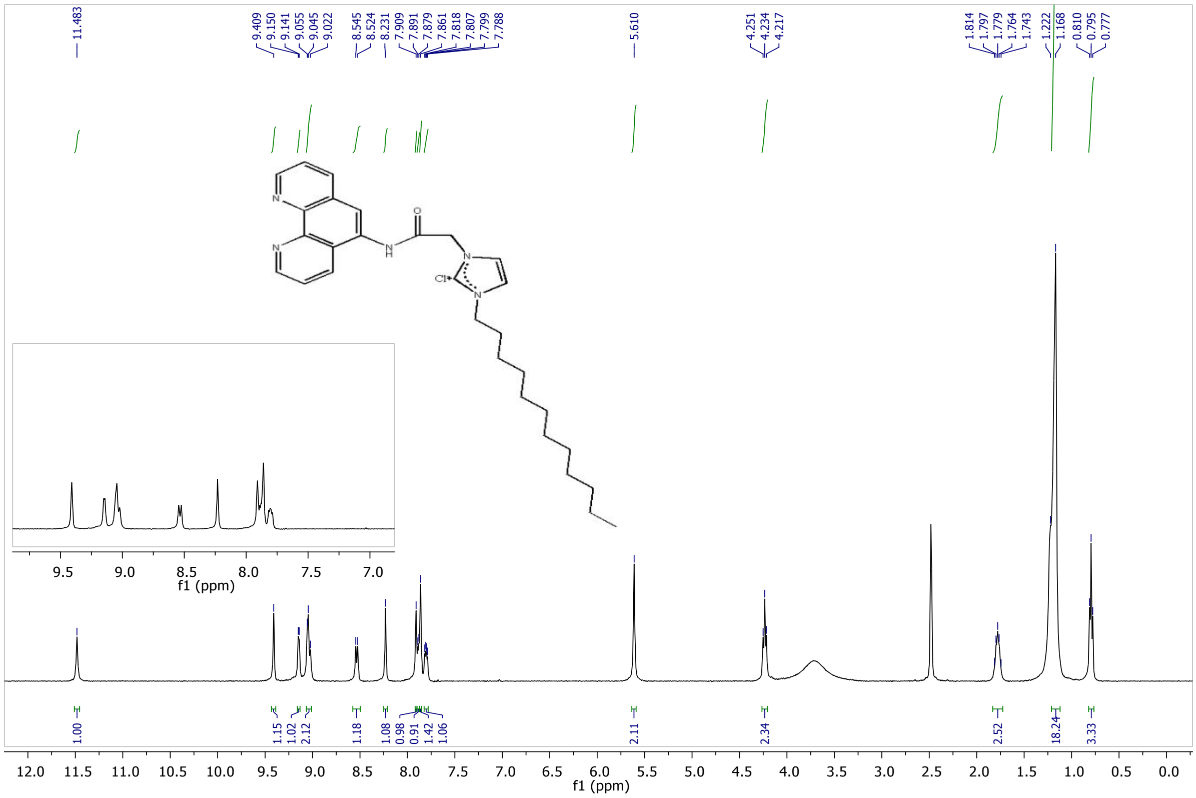

Figure S13. ^{1}H-NMR spectrum of **3d** (DMSO-d_6).

^{13}C-NMR spectra of 3d

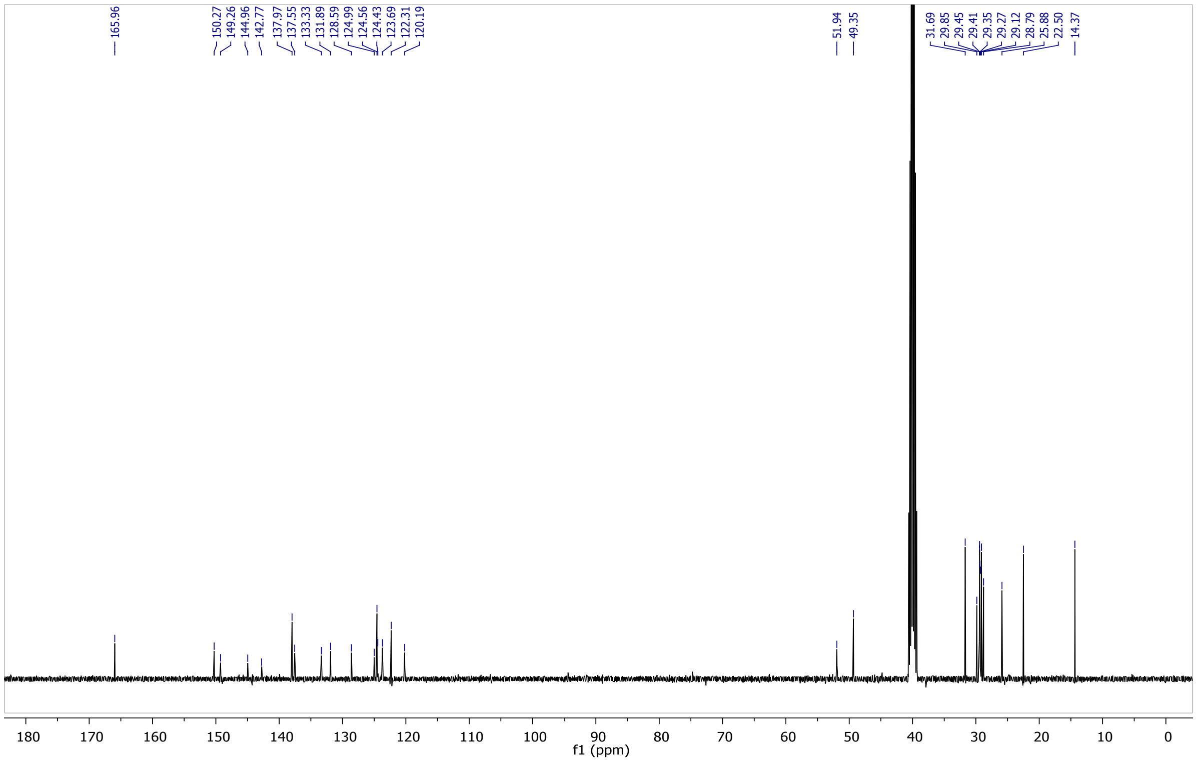

Figure S14. ^{13}C-NMR spectrum of **3d** (DMSO-d_6).

^{1}H-NMR spectra of 3e

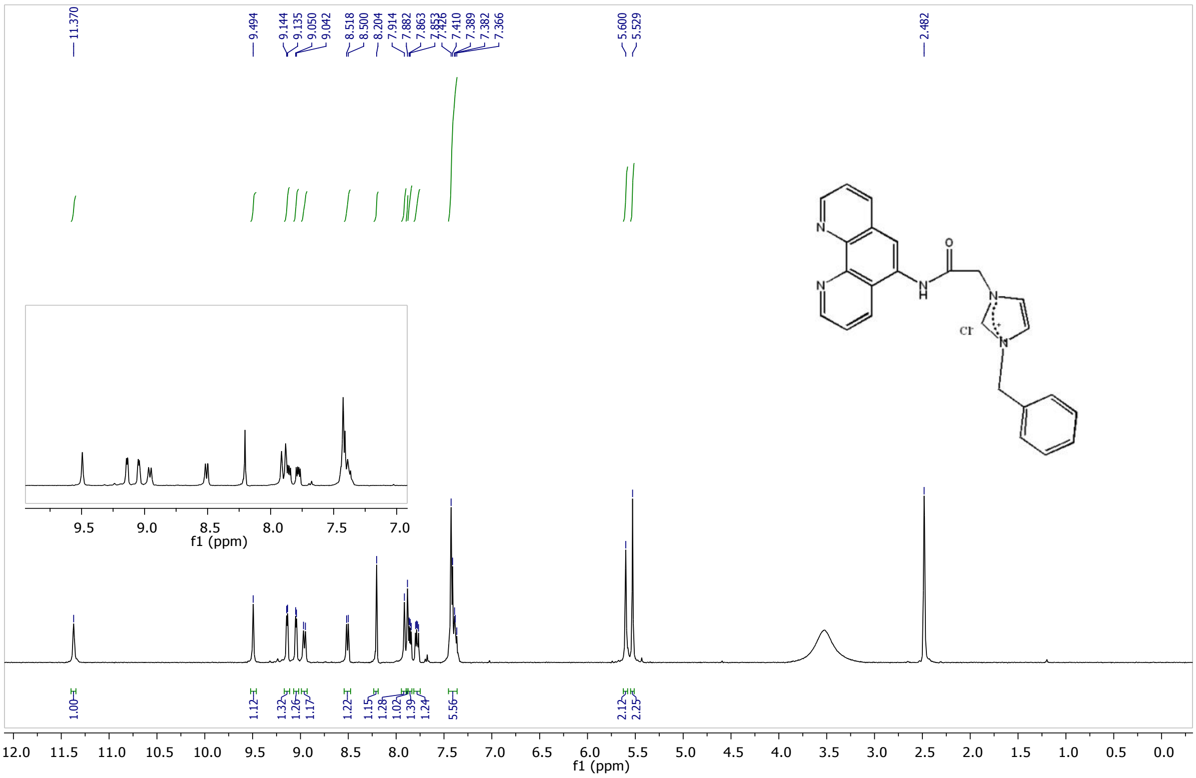

Figure S15. ^{1}H-NMR spectrum of **3e** (DMSO-d_6).

^{13}C-NMR spectra of 3e

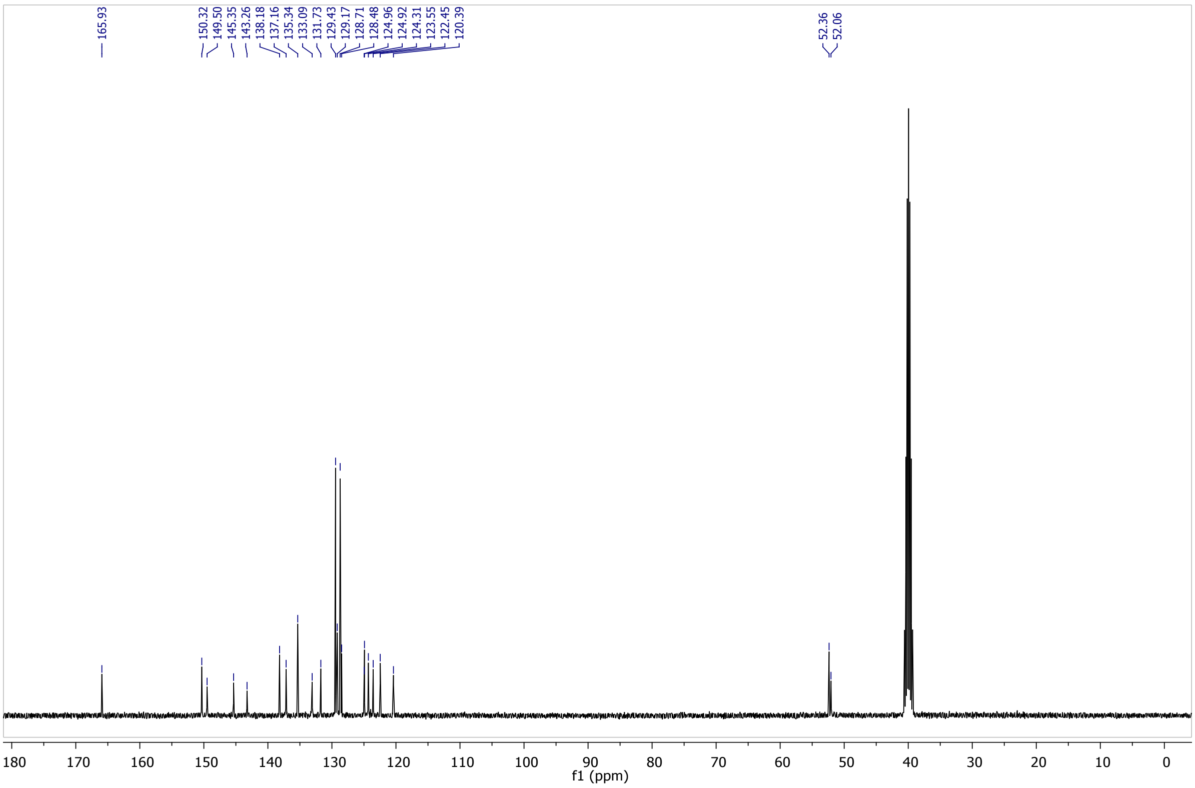

Figure S16. ^{13}C-NMR spectrum of **3e** (DMSO-d_6).

^{1}H-NMR spectra of 3f

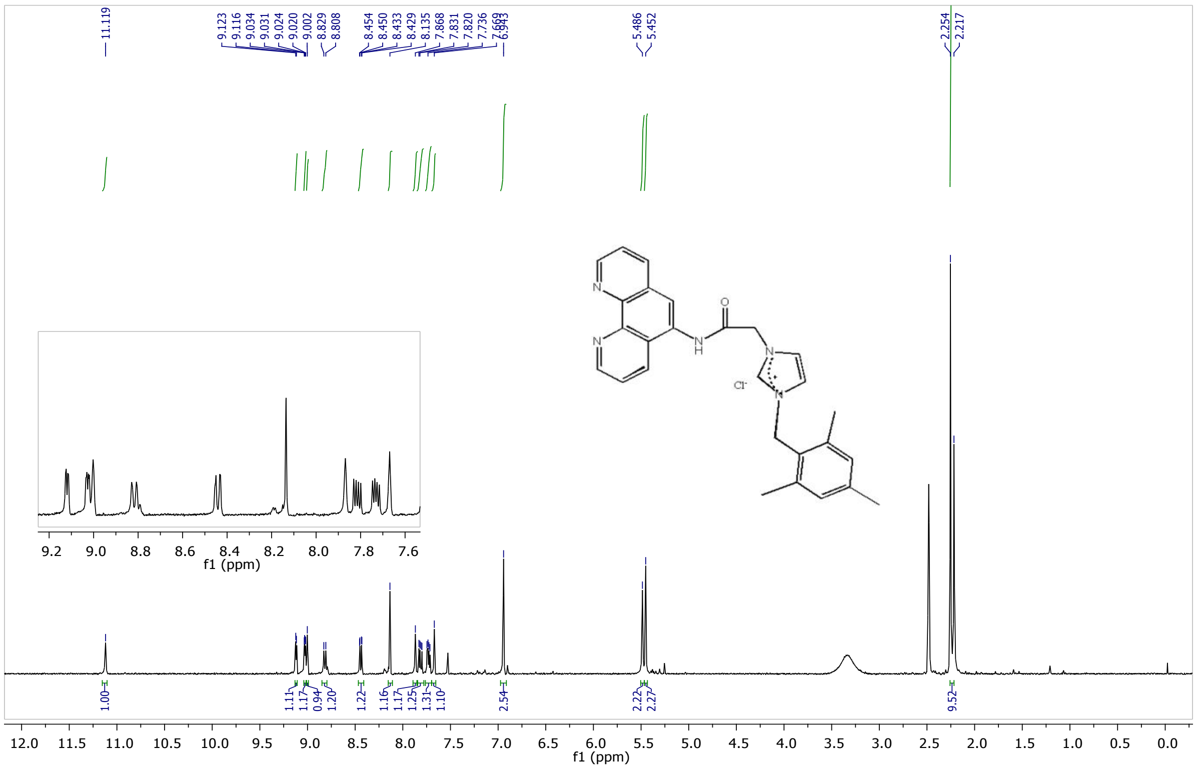

Figure S17. ^{1}H-NMR spectrum of **3f** (DMSO-d_6).

^{13}C-NMR spectra of 3f

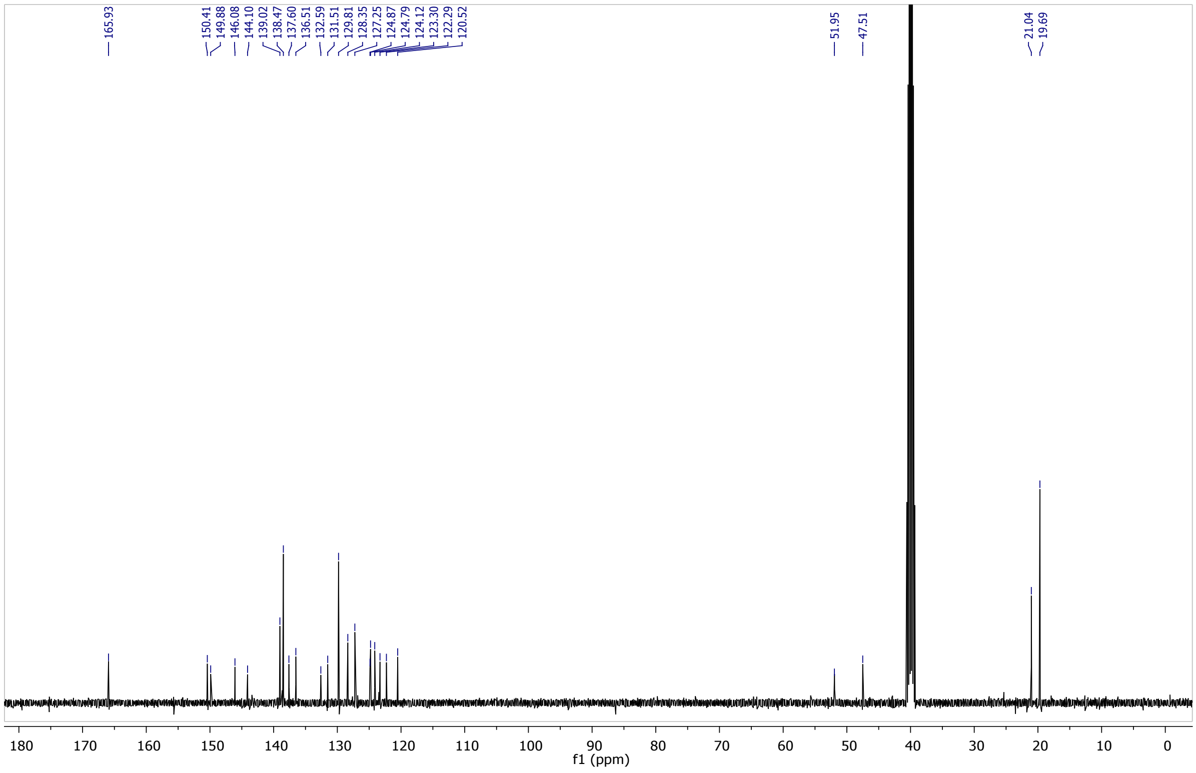

Figure S18. ^{13}C-NMR spectrum of **3f** (DMSO-d_6).

^{1}H-NMR spectra of 3g

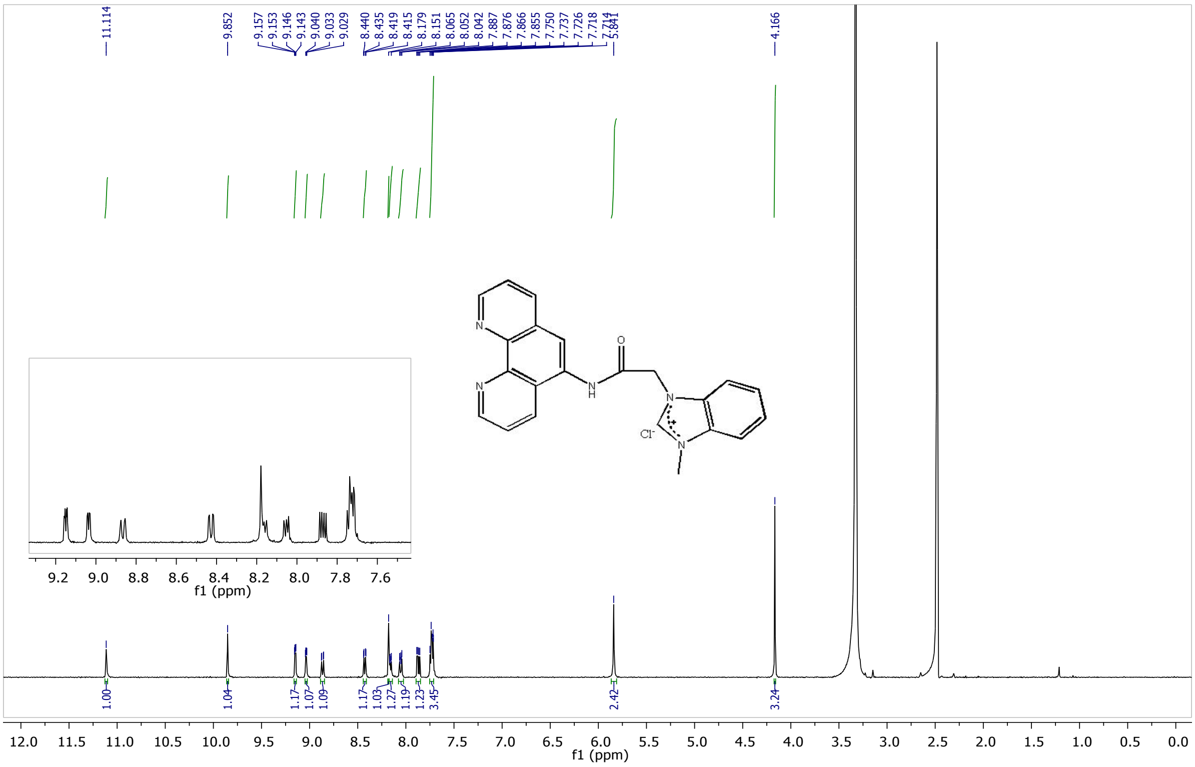

Figure S19. ^{1}H-NMR spectrum of **3g** (DMSO-d_6).

^{13}C-NMR spectra of 3g

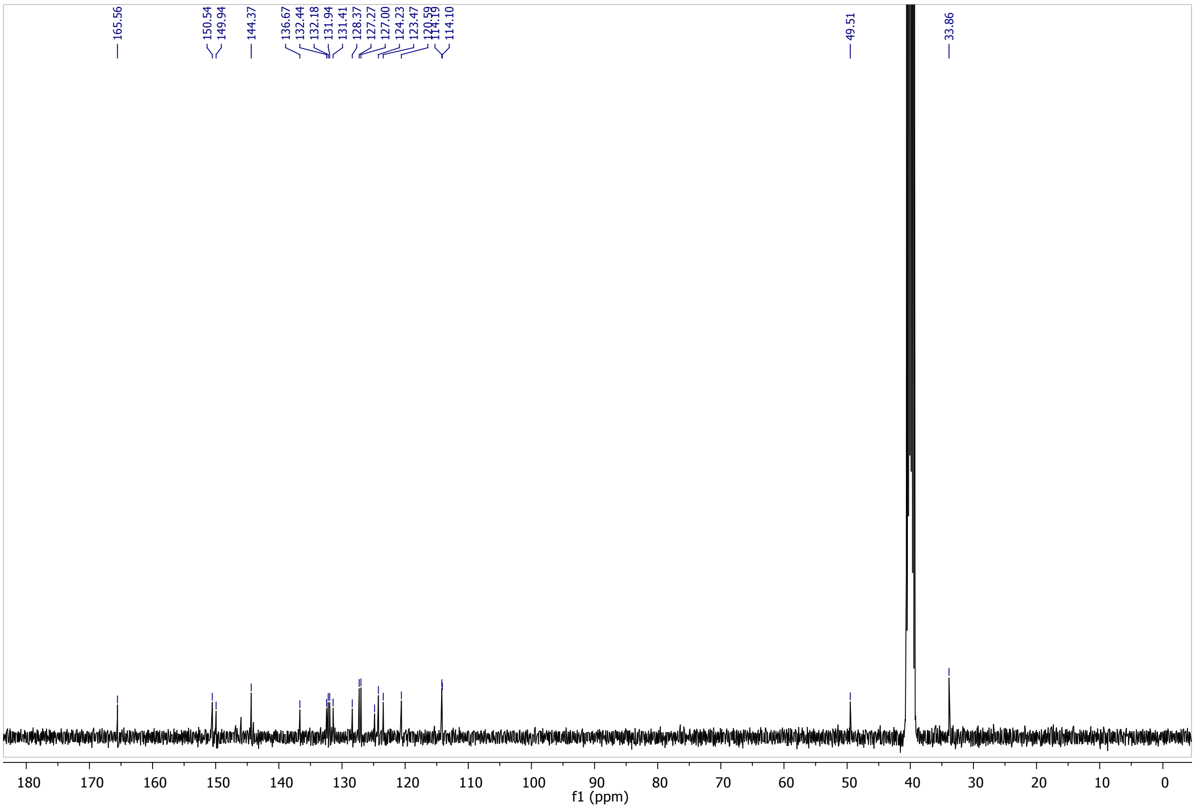

Figure S20. ^{13}C-NMR spectrum of **3g** (DMSO-d_6).

^{1}H-NMR spectra of 3h

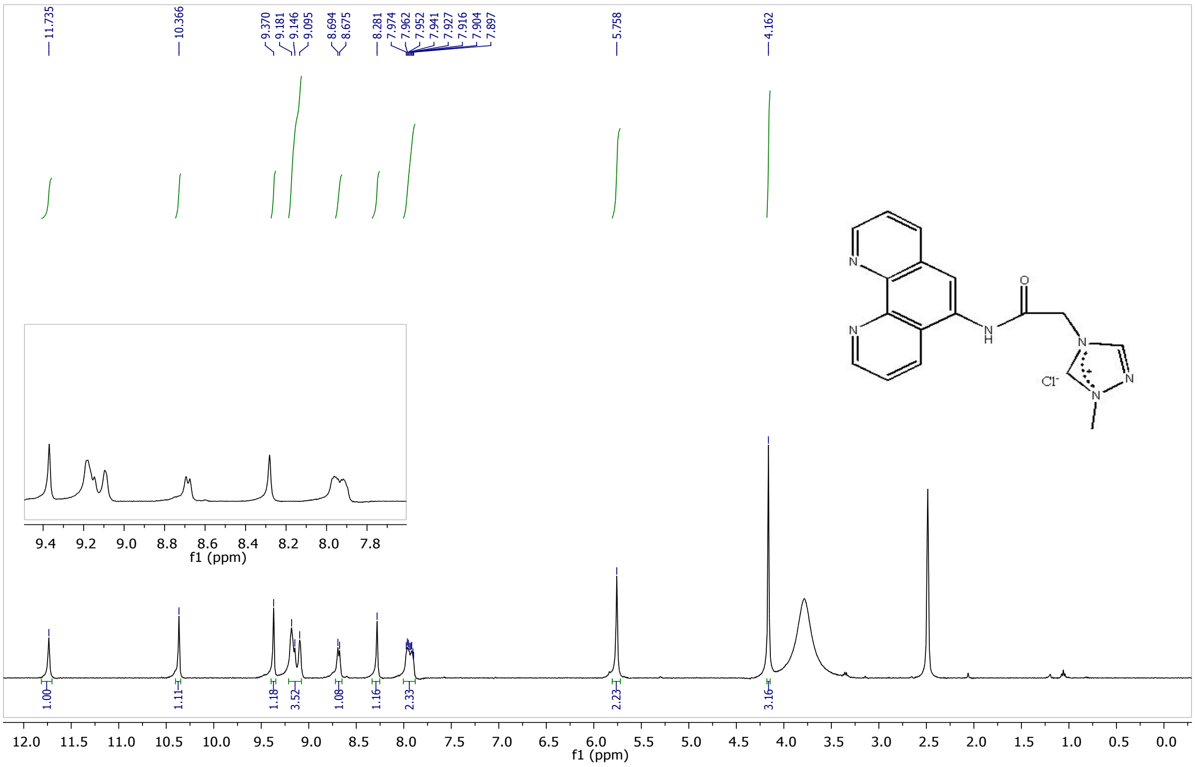

Figure S21. ^{1}H-NMR spectrum of **3h** (DMSO-d_6).

^{13}C-NMR spectra of 3h

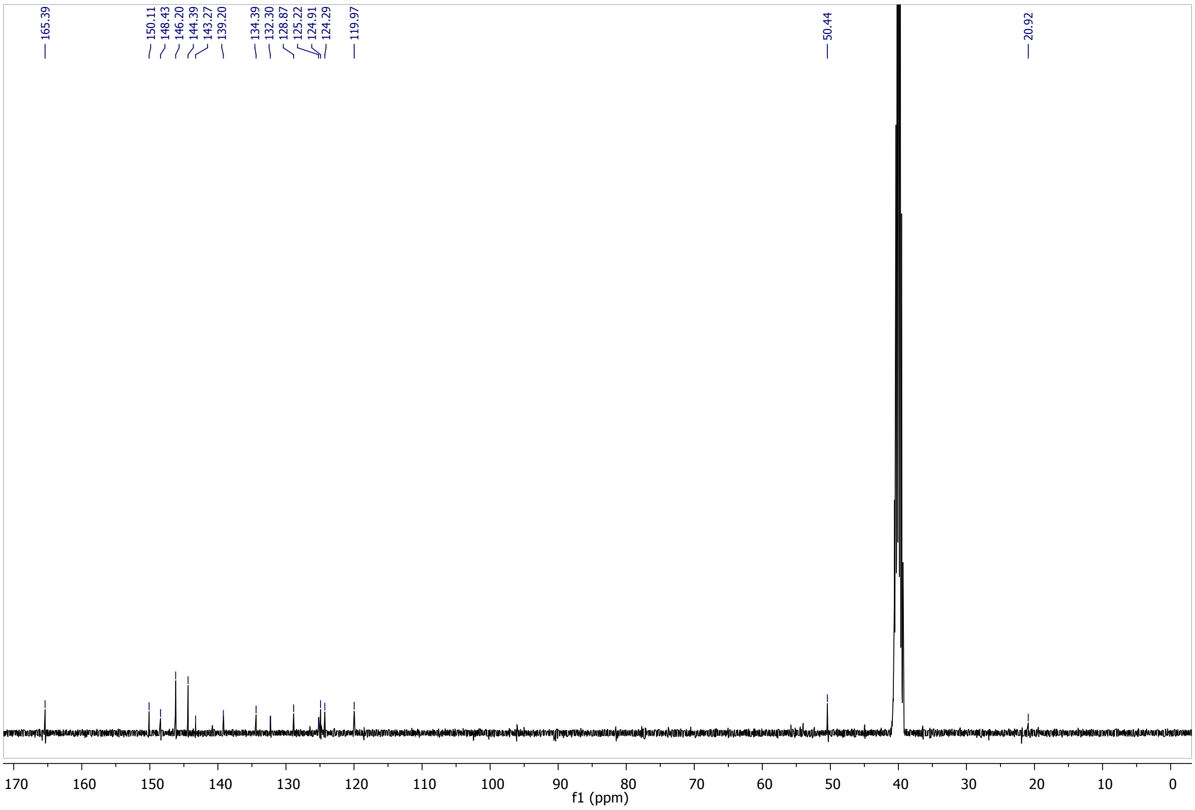

Figure S22. ^{13}C-NMR spectrum of **3h** (DMSO-d_6).

FTIR spectra of 1

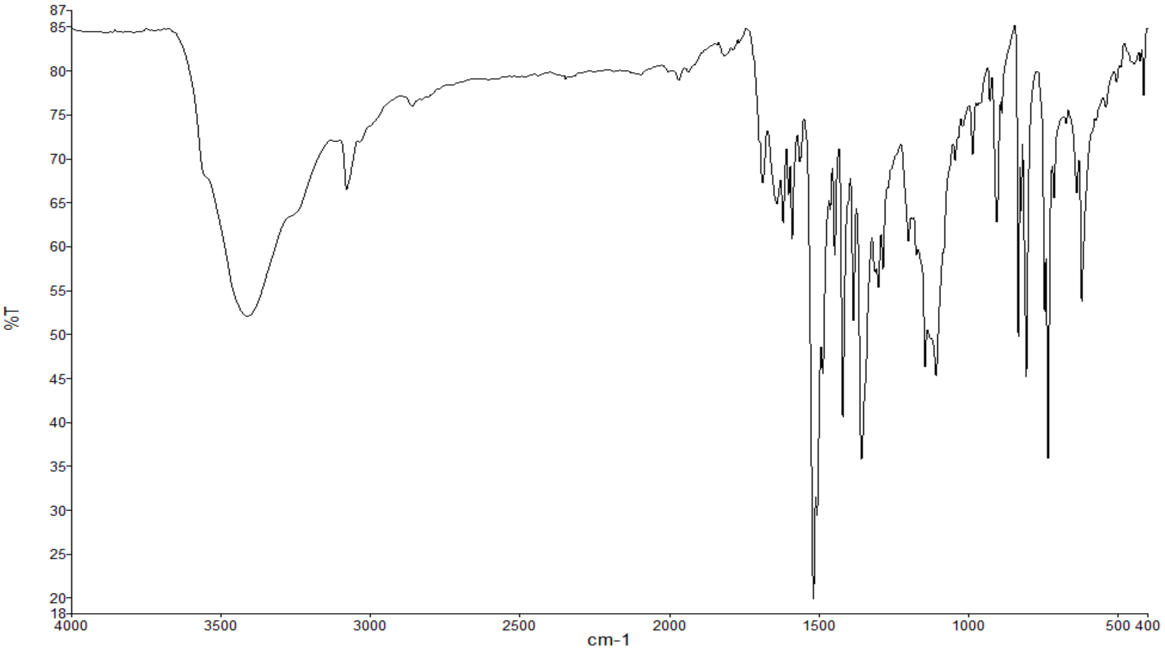

Figure S23. IR spectrum of **1**.

FTIR spectra of 2

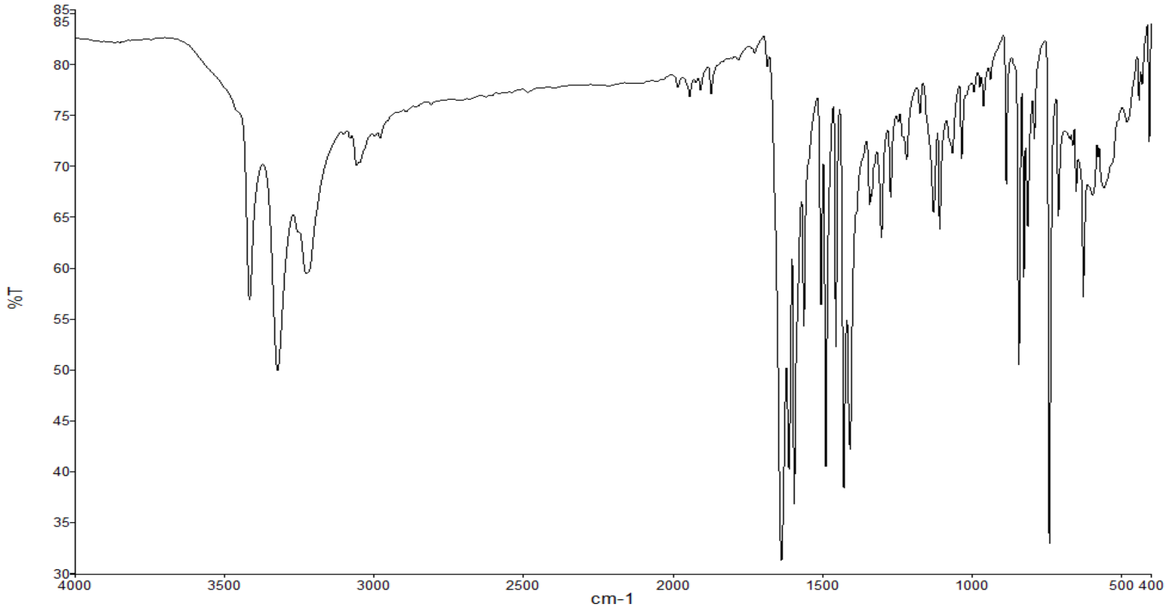

Figure S24. IR spectrum of **2**.

FTIR spectra of 3

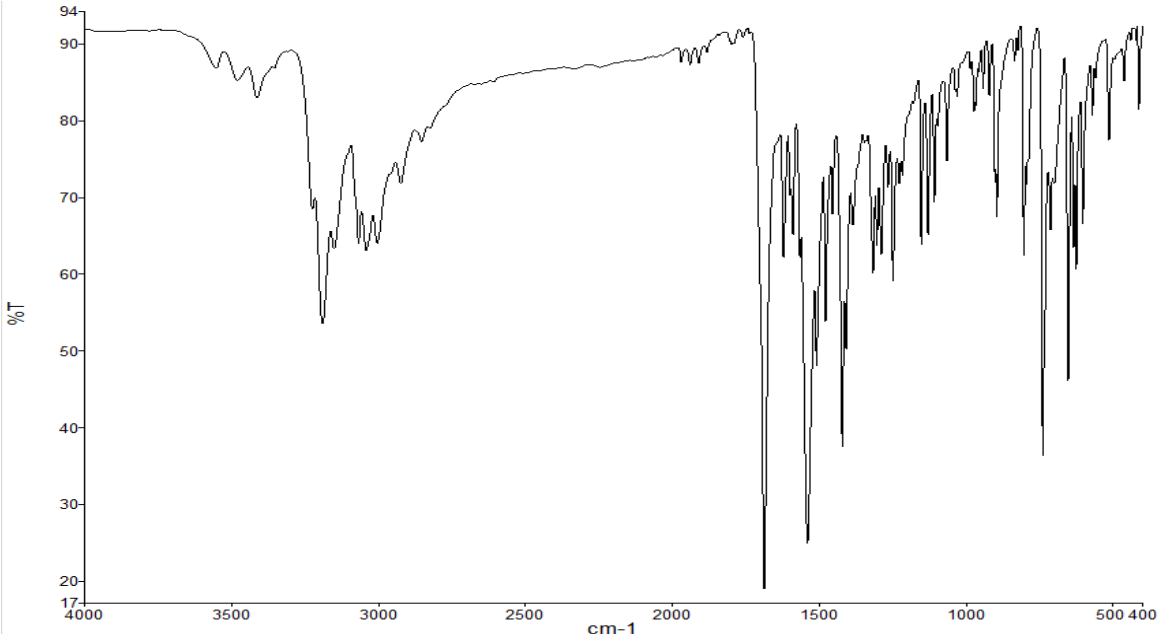

Figure S25. IR spectrum of **3**.

FTIR spectra of 3a

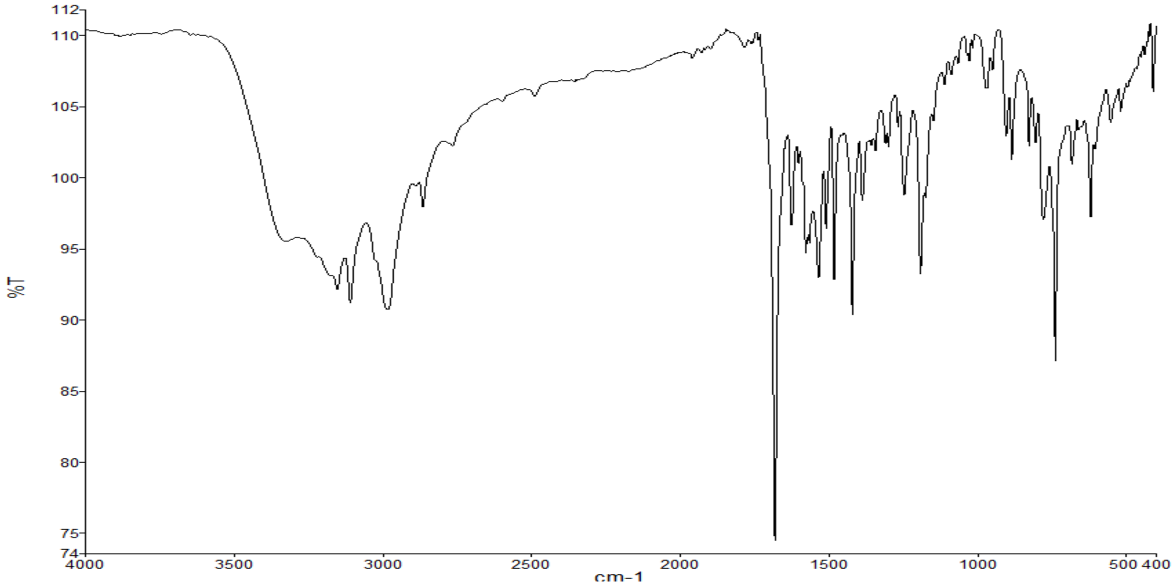

Figure S26. IR spectrum of **3a**.

FTIR spectra of 3b

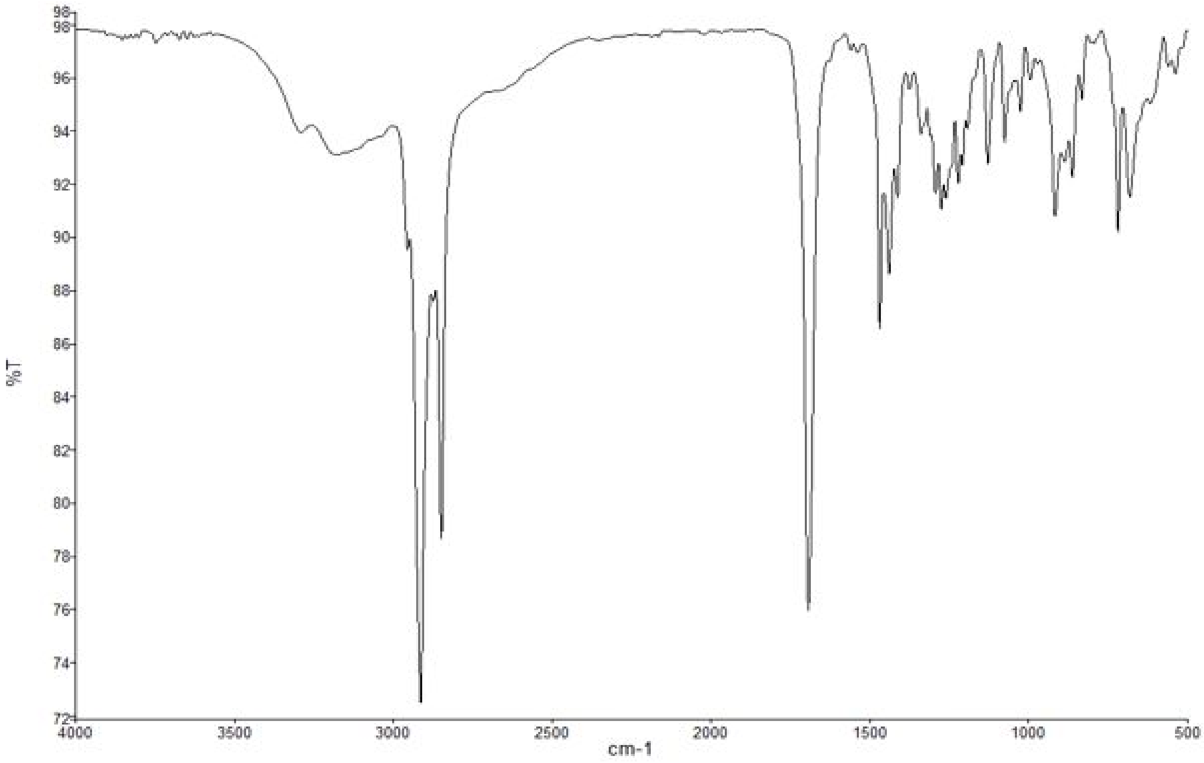

Figure S27. IR spectrum of **3b**.

FTIR spectra of 3c

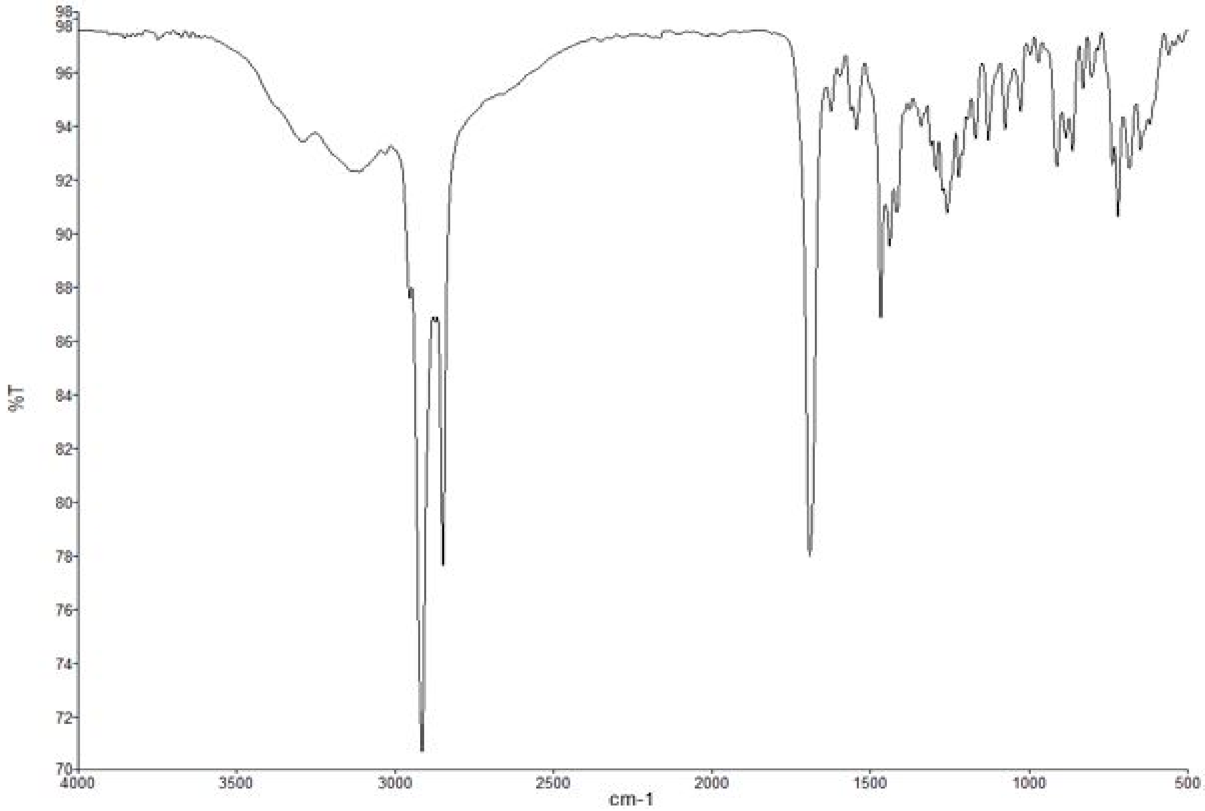

Figure S28. IR spectrum of **3c**.

FTIR spectra of 3d

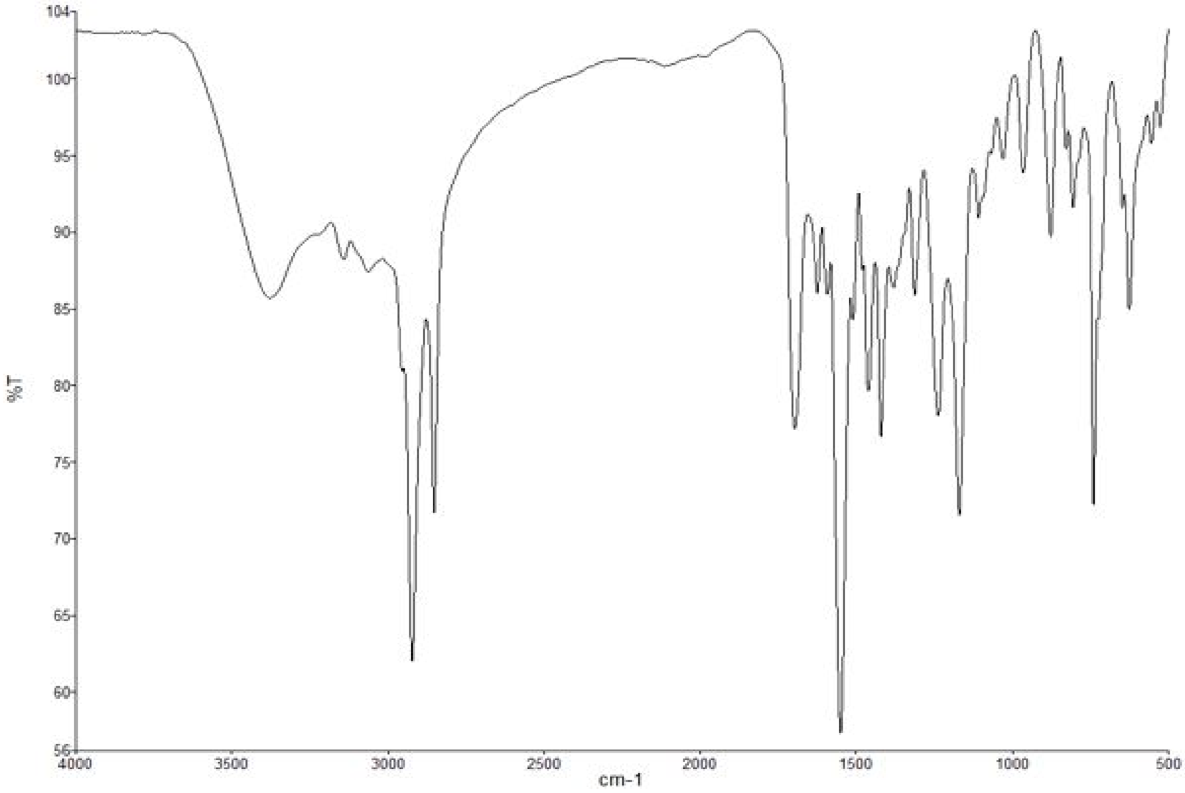

Figure S29. IR spectrum of **3d**.

FTIR spectra of 3e

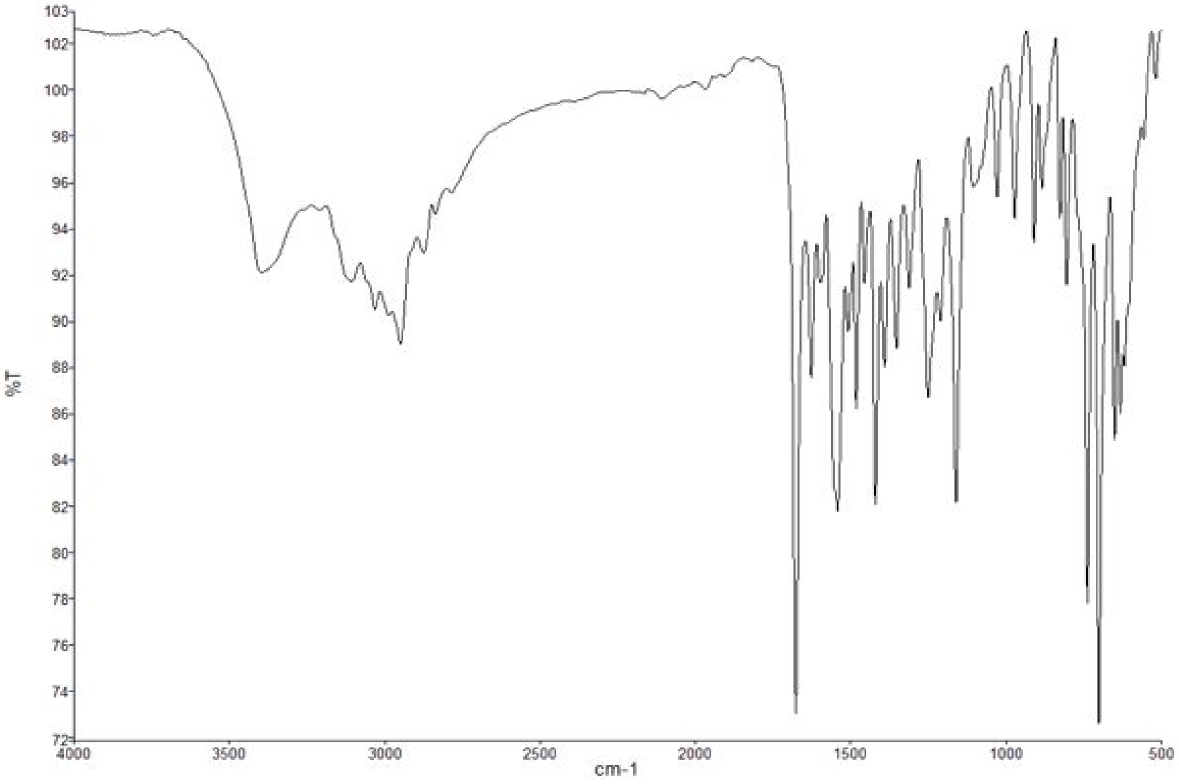

Figure S30. IR spectrum of **3e**.

FTIR spectra of 3f

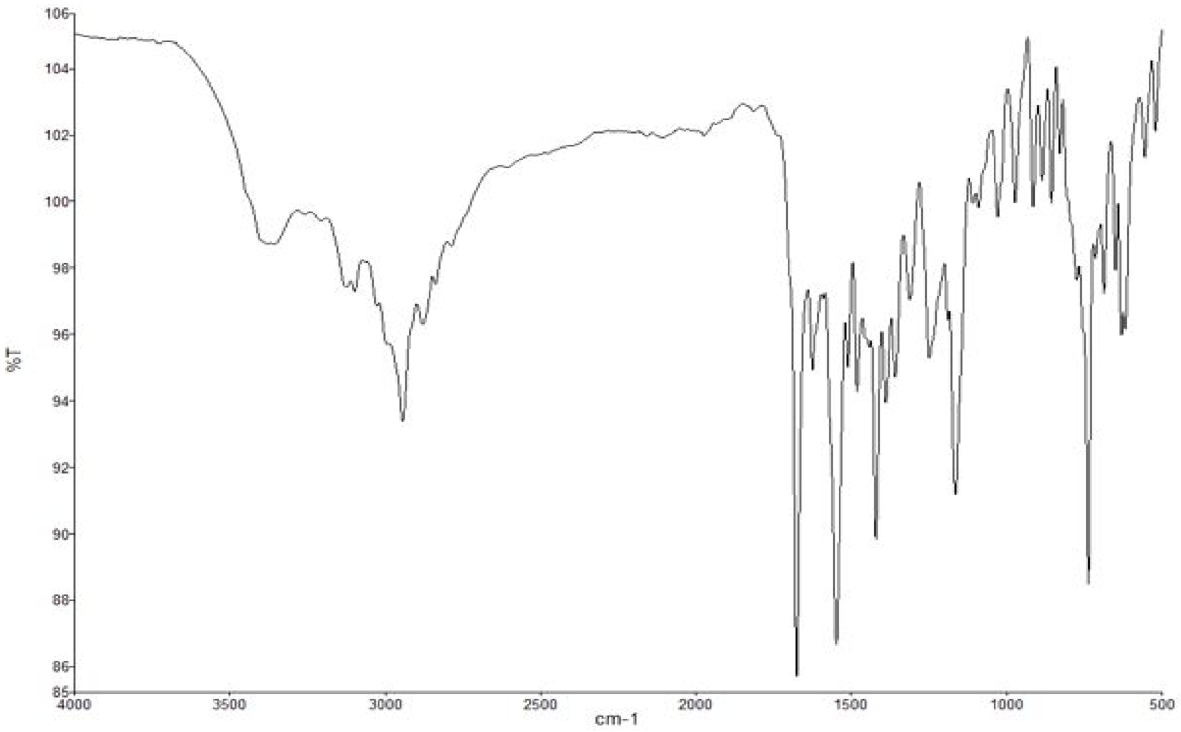

Figure S31. IR spectrum of **3f**.

FTIR spectra of 3g

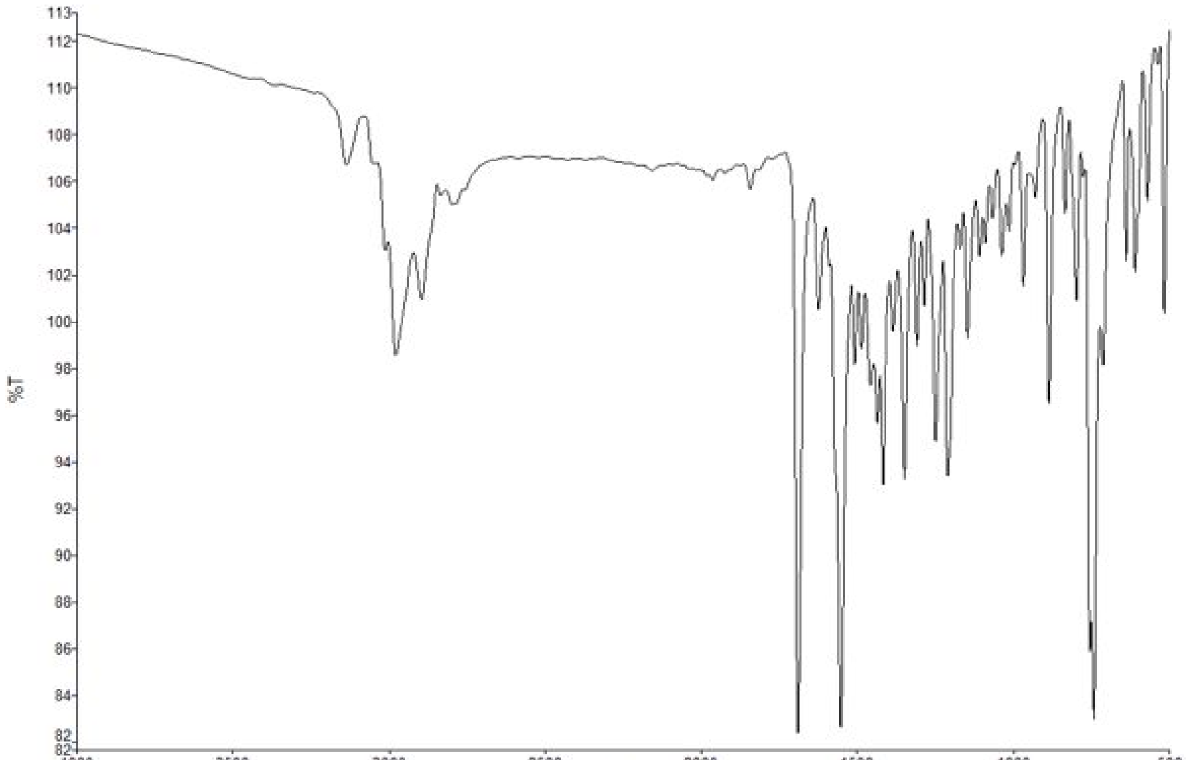

Figure S32. IR spectrum of **3g**.

FTIR spectra of 3h

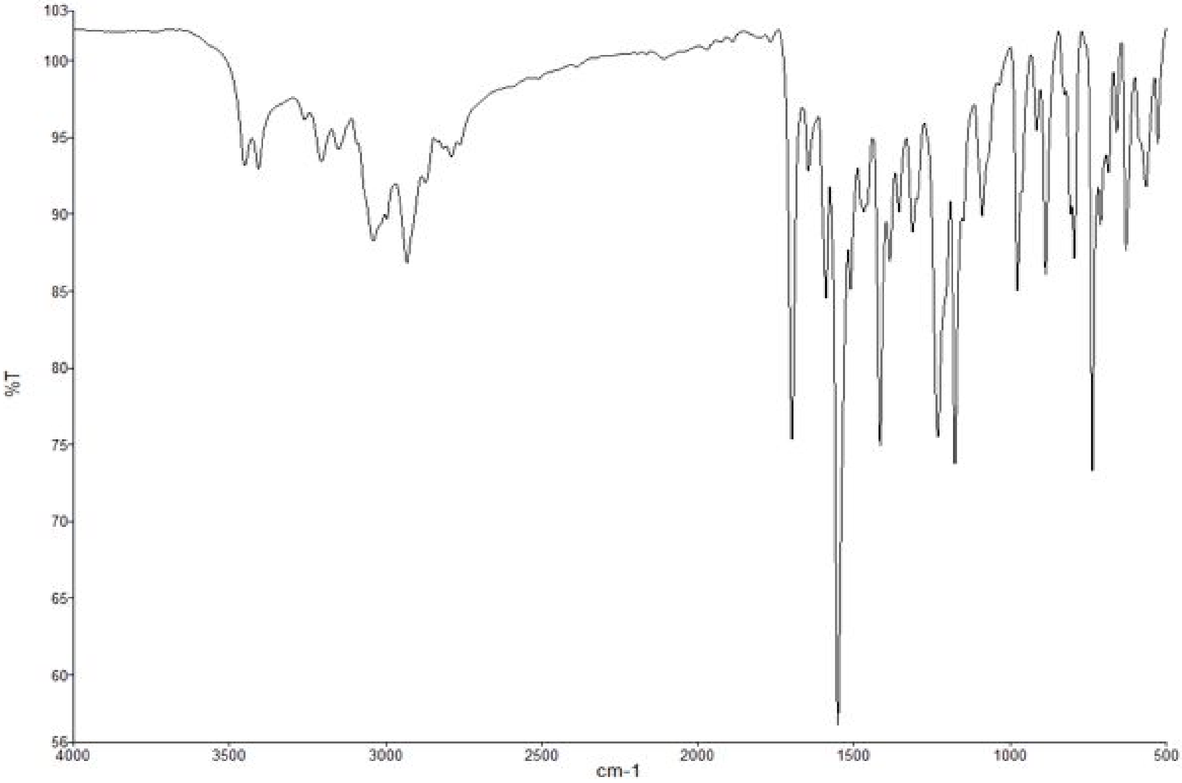

Figure S33. IR spectrum of **3h**.

HRMS analysis of 3a

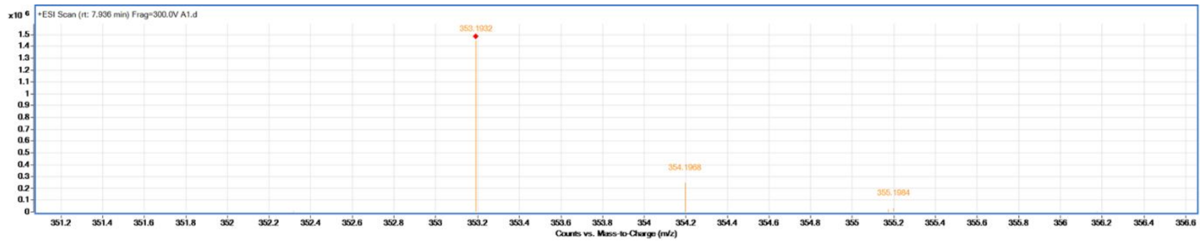

Figure S34. HRMS analysis report of compound **3a**

HRMS analysis of 3b

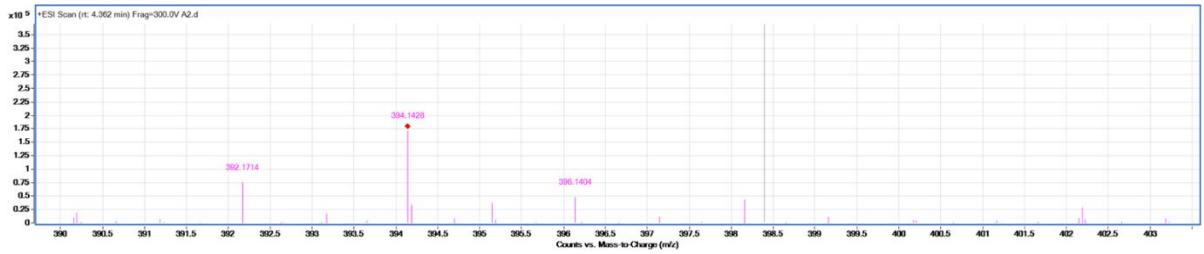

Figure S35. HRMS analysis report of compound **3b**

HRMS analysis of 3c

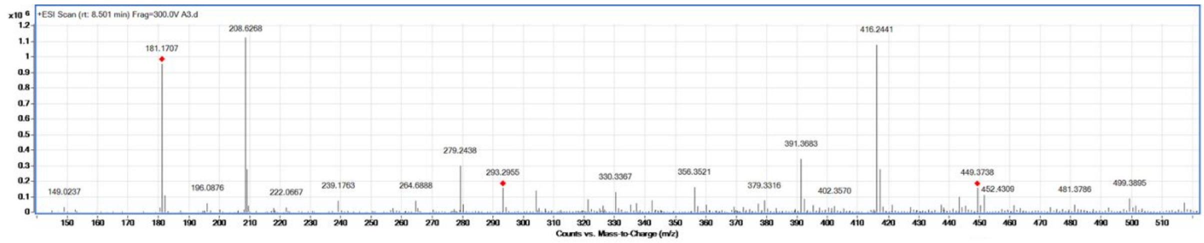

Figure S36. HRMS analysis report of compound **3c**

HRMS analysis of 3d

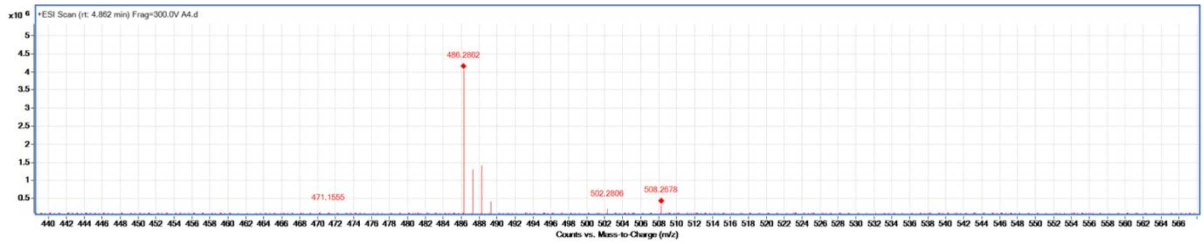

Figure S37. HRMS analysis report of compound **3d**

HRMS analysis of 3e

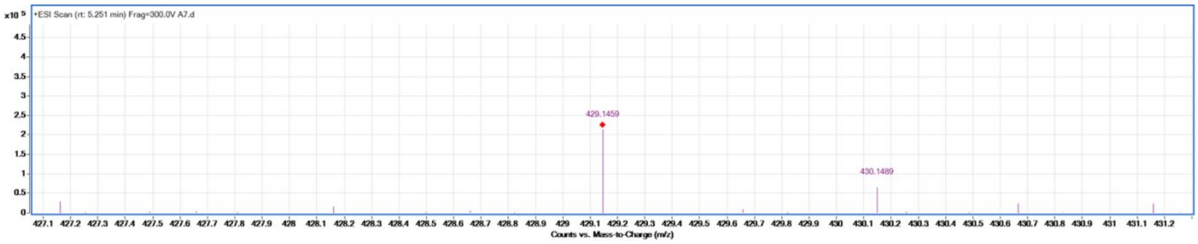

Figure S38. HRMS analysis report of compound **3e**

HRMS analysis of 3f

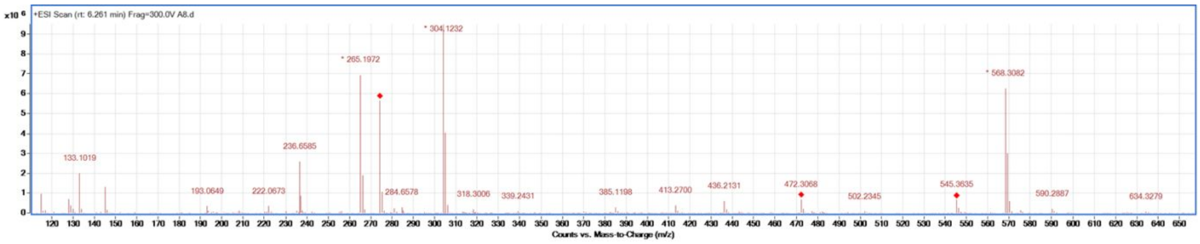

Figure S39. HRMS analysis report of compound **3f**

HRMS analysis of 3g

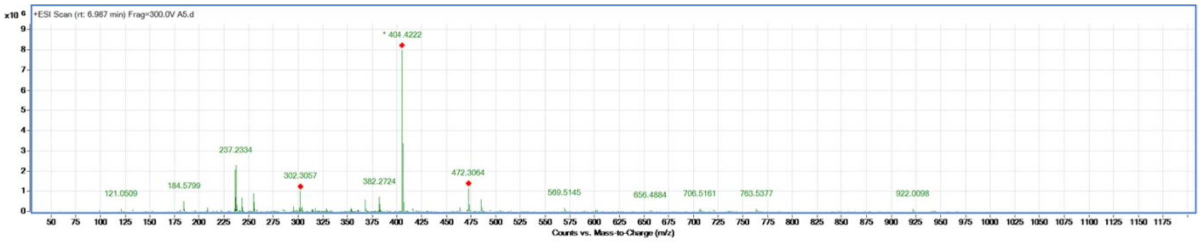

Figure S40. HRMS analysis report of compound **3g**

HRMS analysis of 3h

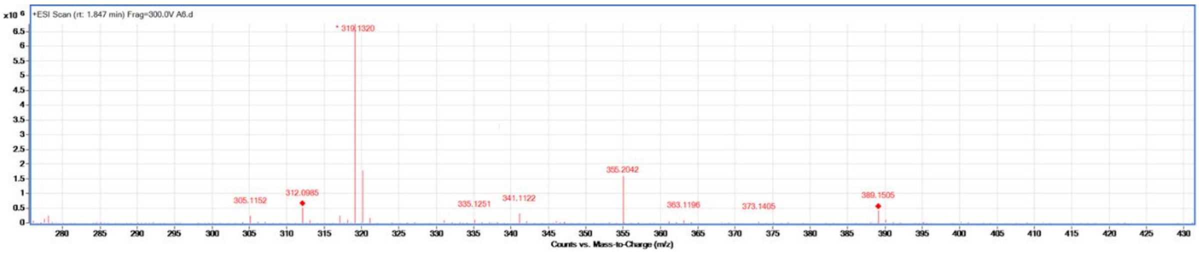

Figure S41. HRMS analysis report of compound **3h**

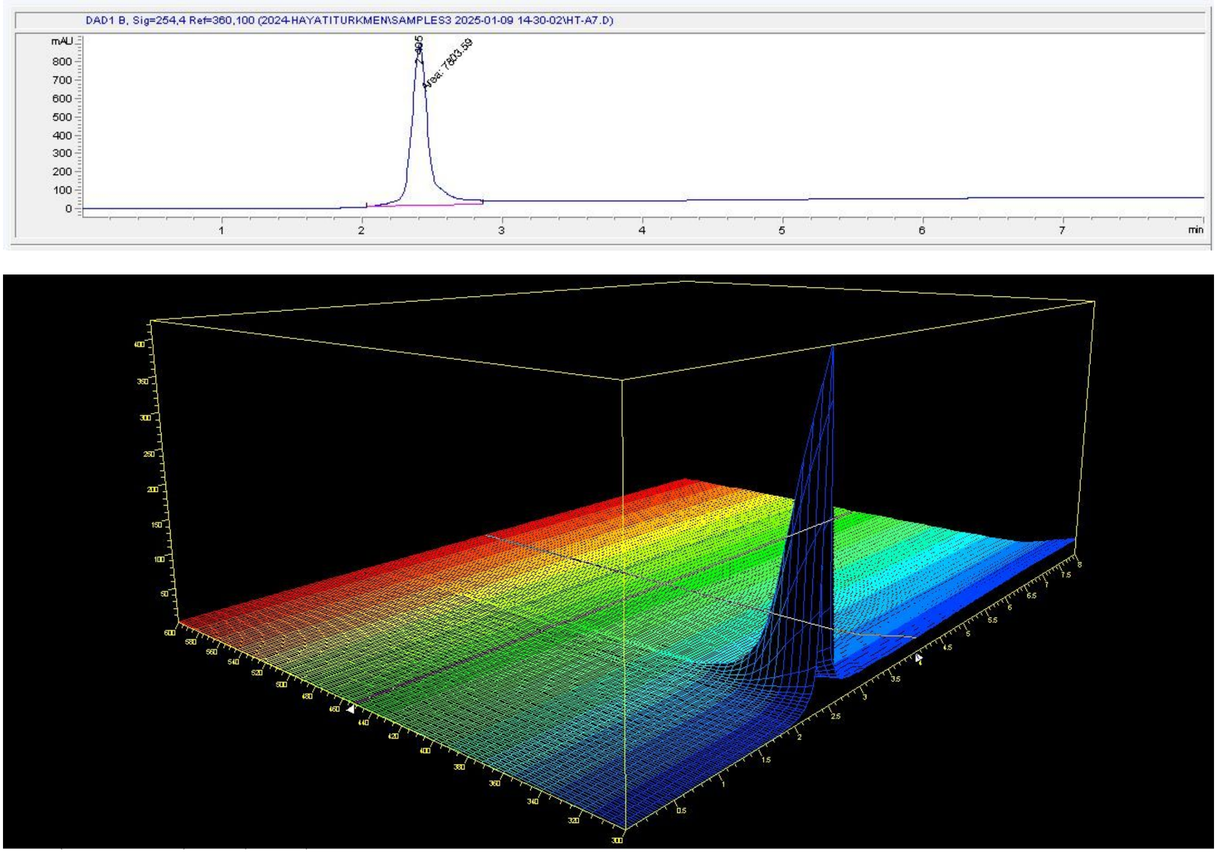

Figure S42. HPLC analysis report of compound **3e**

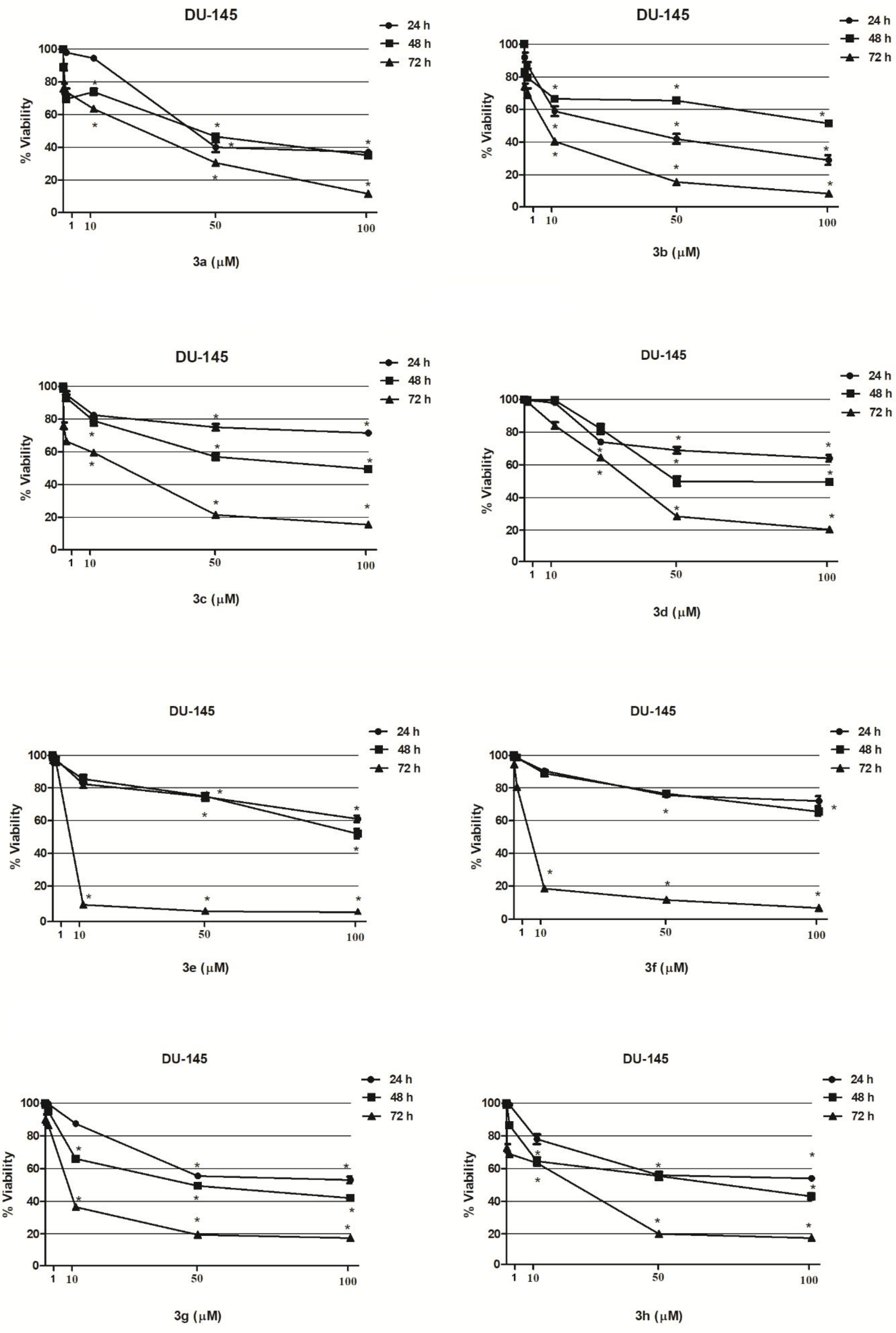

Figure S43. XTT results of the compound **3a–h** effect for DU-145 cells.

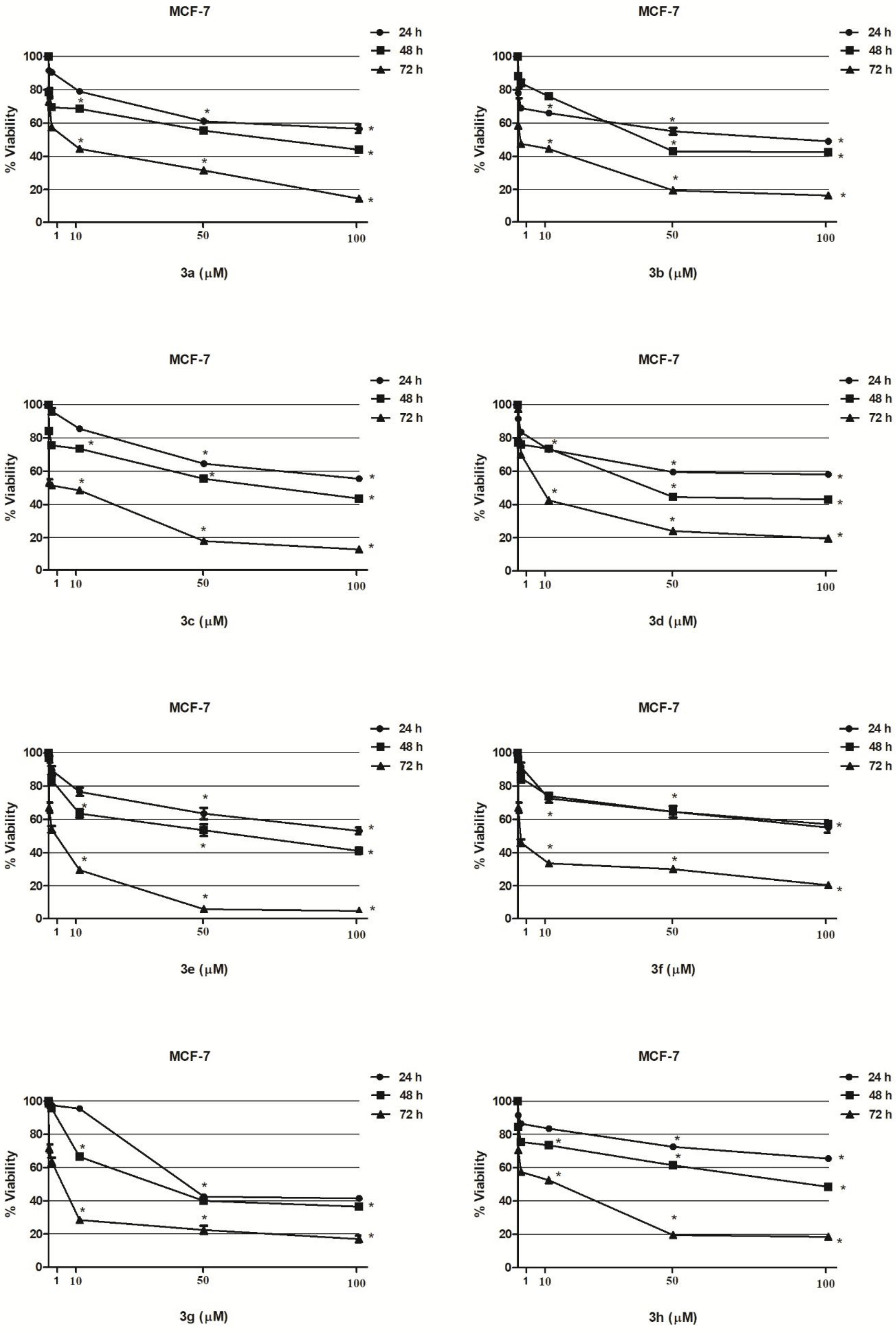

Figure S44. XTT results of the compound **3a–h** effect for MCF-7 cells.

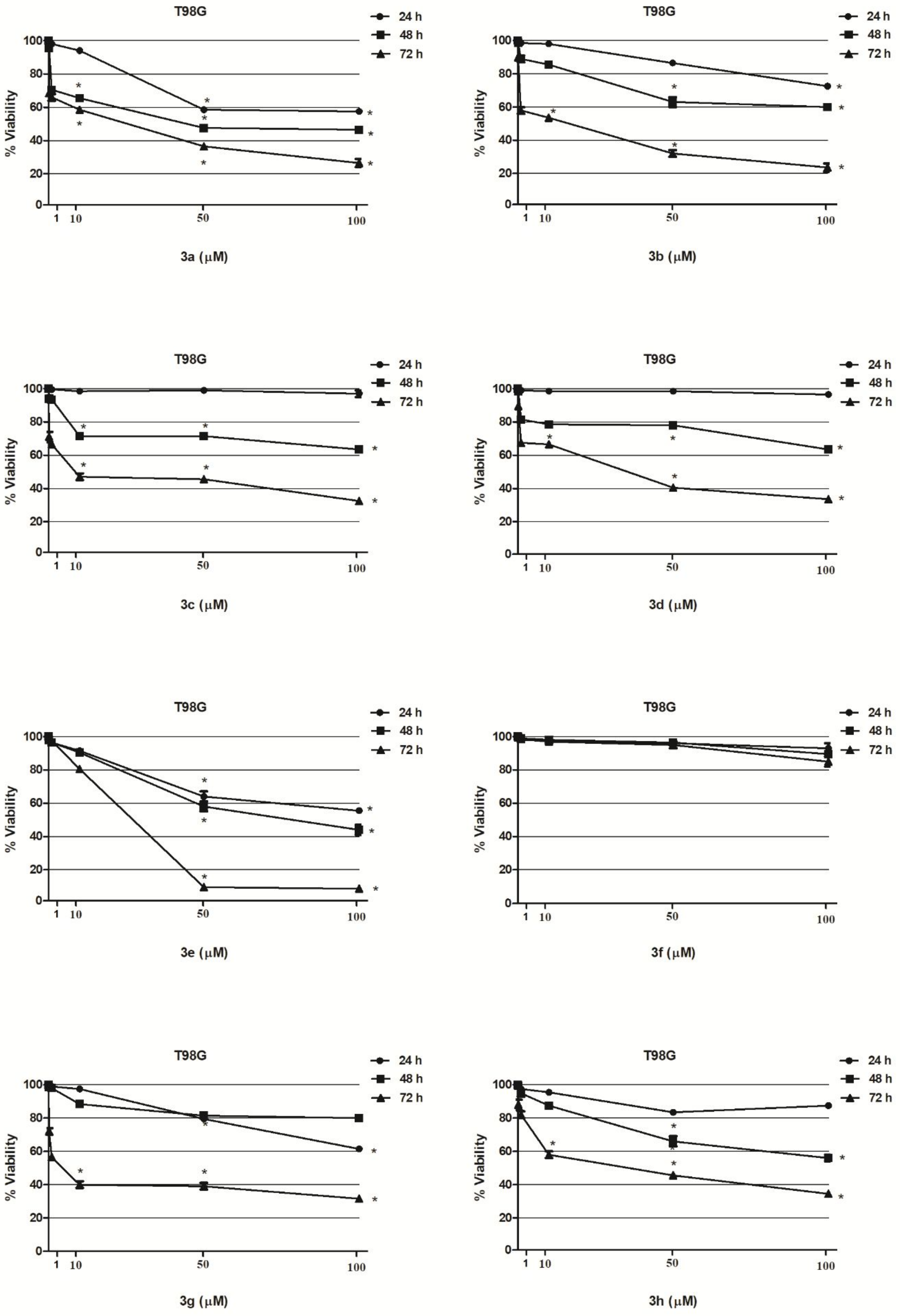

Figure S45. XTT results of the compound **3a–h** effect for T98G cells.

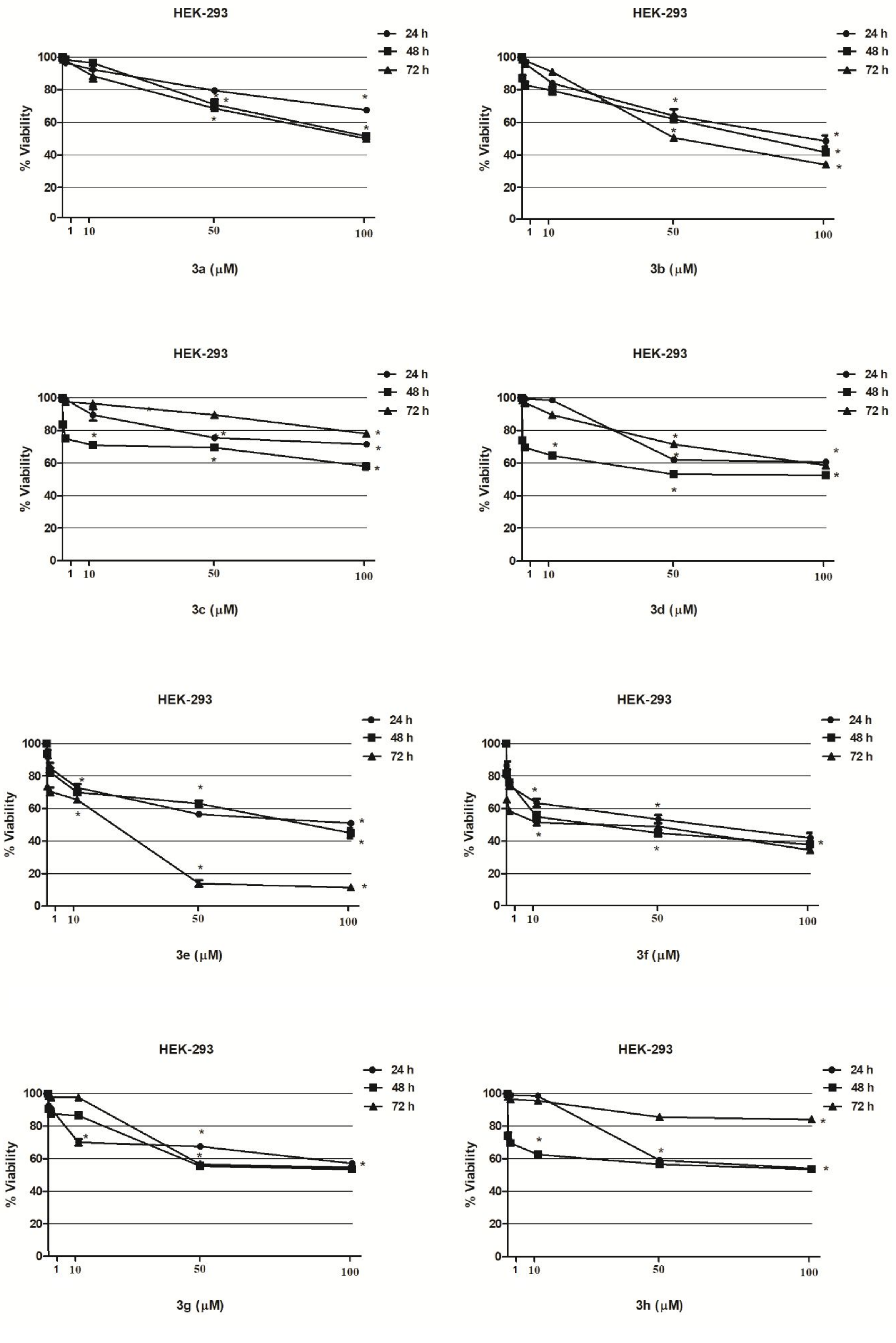

Figure S46. XTT results of the compound **3a–h** effect for HEK-293 cells.

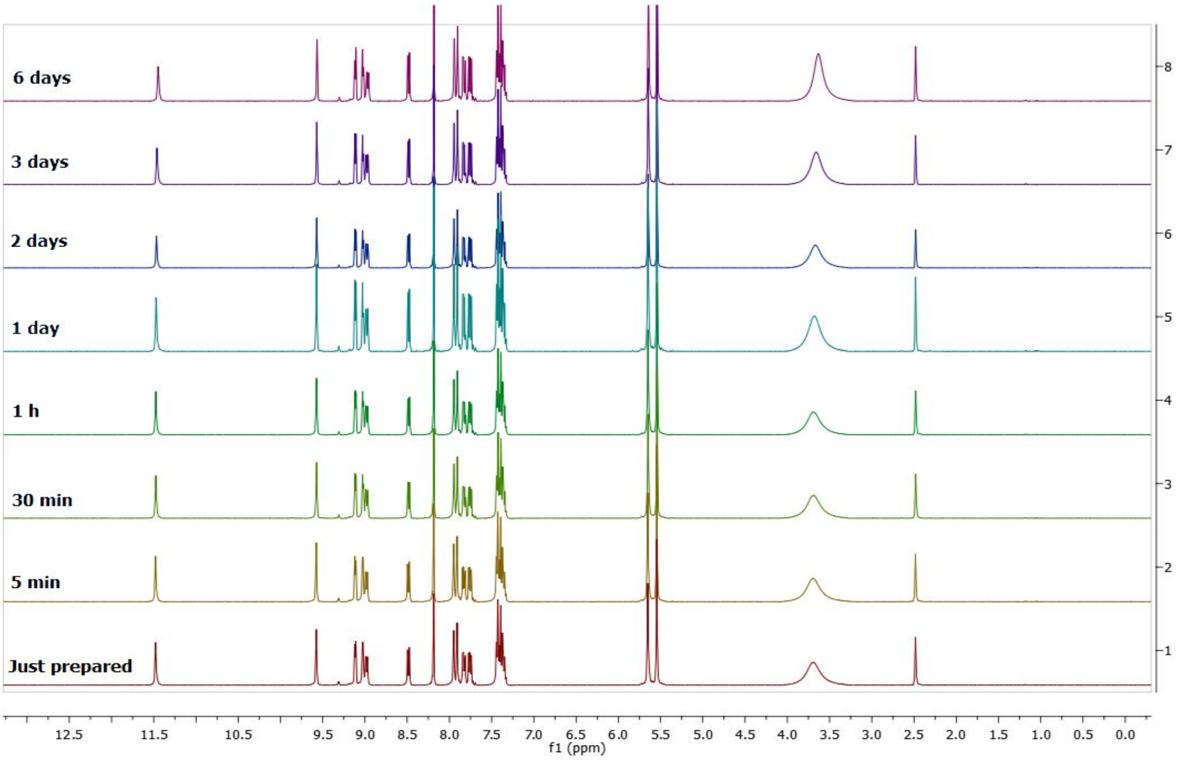

Figure S47. The stability of **3e** was tracked via ^{1}H NMR spectroscopy in DMSO-d_6 for over 6 days.

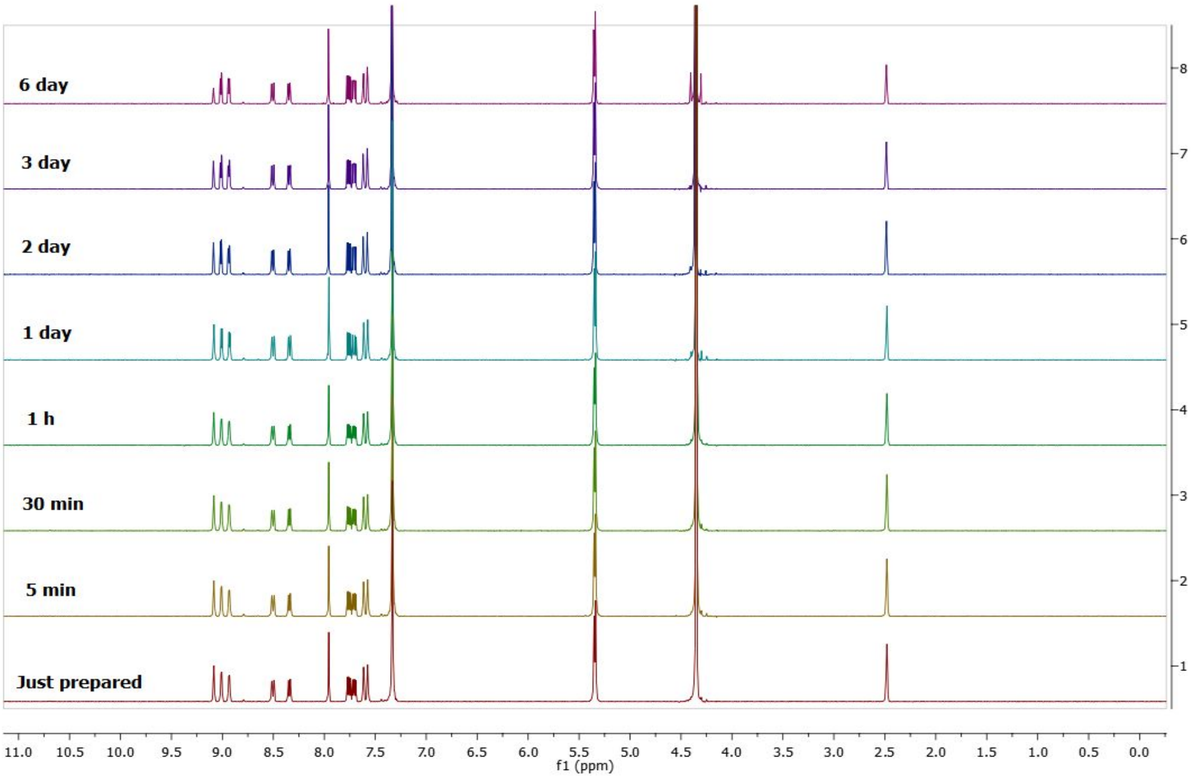

Figure S48. The stability of **3e** was tracked via ^{1}H NMR spectroscopy in 20 % D_2O/DMSO-d_6 for over 6 days.

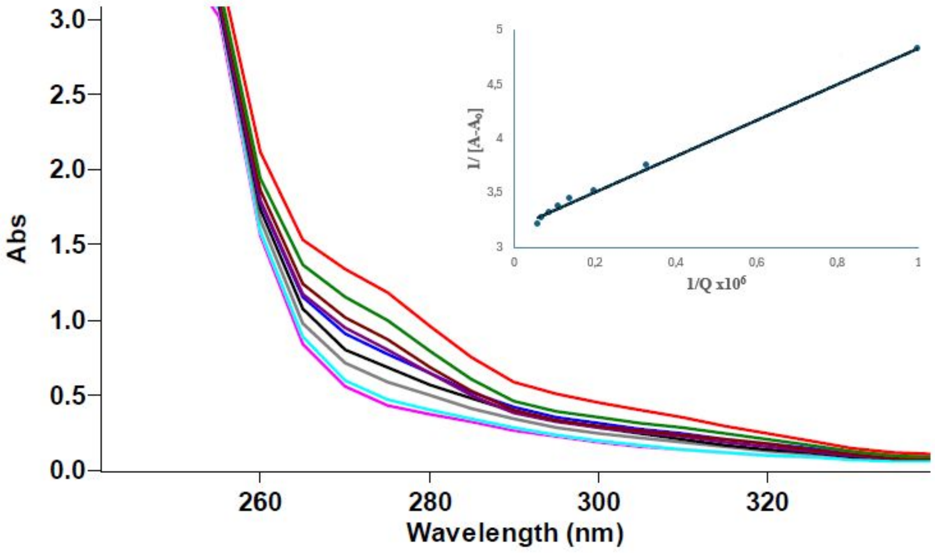

Figure S49. UV-Vis spectra of FS-DNA with different concentrations of **3e** K_b was calculated by the ratio of intercept and slope of plot between $1/(A_0\text{-}A)$ and $1/[Q]$.

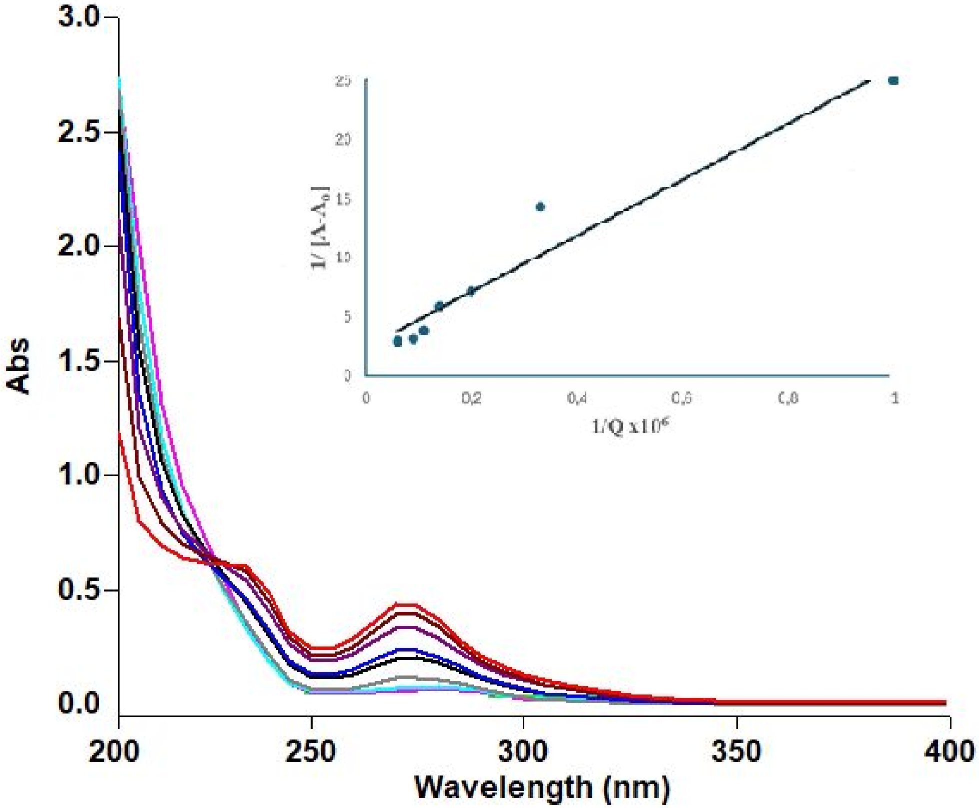

Figure S50. UV-Vis spectra of BSA with different concentrations of **3e** K_b was calculated by the ratio of intercept and slope of plot between $1/(A_0\text{-}A)$ and $1/[Q]$.
